# Supplementary material for: Interaction with AK2A links AIFM1 to cellular energy metabolism
Source: Mol Cell. Author manuscript; Available in PMC 2025 Jul 30. (PMC7617965; doi:10.1016/j.molcel.2025.05.036)
Supplement: Document S1. [file EMS207342-supplement-Document_S1_.pdf]

Molecular Cell, Volume *85*

Supplemental information

Interaction with AK2A links AIFM1 to cellular energy metabolism

Robin Alexander Rothemann, Egor Pavlenko, Mrityunjoy Mondal, Sarah Gerlich, Pavel Grobushkin, Sebastian Mostert, Julia Racho, Konstantin Weiss, Dylan Stobbe, Katharina Stillger, Kim Lapacz, Silja Lucia Salscheider, Carmelina Petrungaro, Dan Ehninger, Thi Hoang Duong Nguyen, Jörn Dengjel, Ines Neundorf, Daniele Bano, Simon Poepsel, and Jan Riemer

SUPPLEMENTAL INFORMATION

Interaction with AK2A links AIFM1 to cellular energy metabolism

Robin Alexander Rothemann[1#], Egor Pavlenko[2#], Mrityunjoy Mondal[3], Sarah Gerlich[1], Pavel Grobushkin[1], Sebastian Mostert[1], Julia Racho[1], Konstantin Weiss[1], Dylan Stobbe[1], Katharina Stillger[4], Kim Lapacz[1], Silja Lucia Salscheider[1], Carmelina Petrungaro[1], Dan Ehninger[3], Thi Hoang Duong Nguyen[5], Jörn Dengjel[6], Ines Neundorf[4], Daniele Bano[3], Simon Poepsel[2,7,#,*], Jan Riemer[1,7,#,*,$]

1, Redox Metabolism Group, Institute for Biochemistry, University of Cologne, D-50674 Cologne, Germany

2, Center for Molecular Medicine Cologne (CMMC), Faculty of Medicine and University Hospital, University of Cologne, D-50931 Cologne, Germany.

3, German Center for Neurodegenerative Diseases (DZNE), D-53127 Bonn, Germany.

4, Peptide Biochemistry Group, Institute for Biochemistry, University of Cologne, D-50674 Cologne, Germany

5, MRC Laboratory of Molecular Biology, Cambridge, CB2 0QH, UK

6, Department of Biology, University of Fribourg, CH-1700 Fribourg, Switzerland

7, Cologne Excellence Cluster on Cellular Stress Responses in Aging-Associated Diseases (CECAD), University of Cologne, D-50931 Cologne, Germany.

#, equal contribution

§, lead contact: J.R., Jan Riemer

* address correspondence to

J.R.: jan.riemer@uni-koeln.de, +49-221-470-7306, ORCID 0000-0002-7574-8457,

S.P.: spoepsel@uni-koeln.de, +49-221-478-96987, ORCID 0000-0002-8304-4062

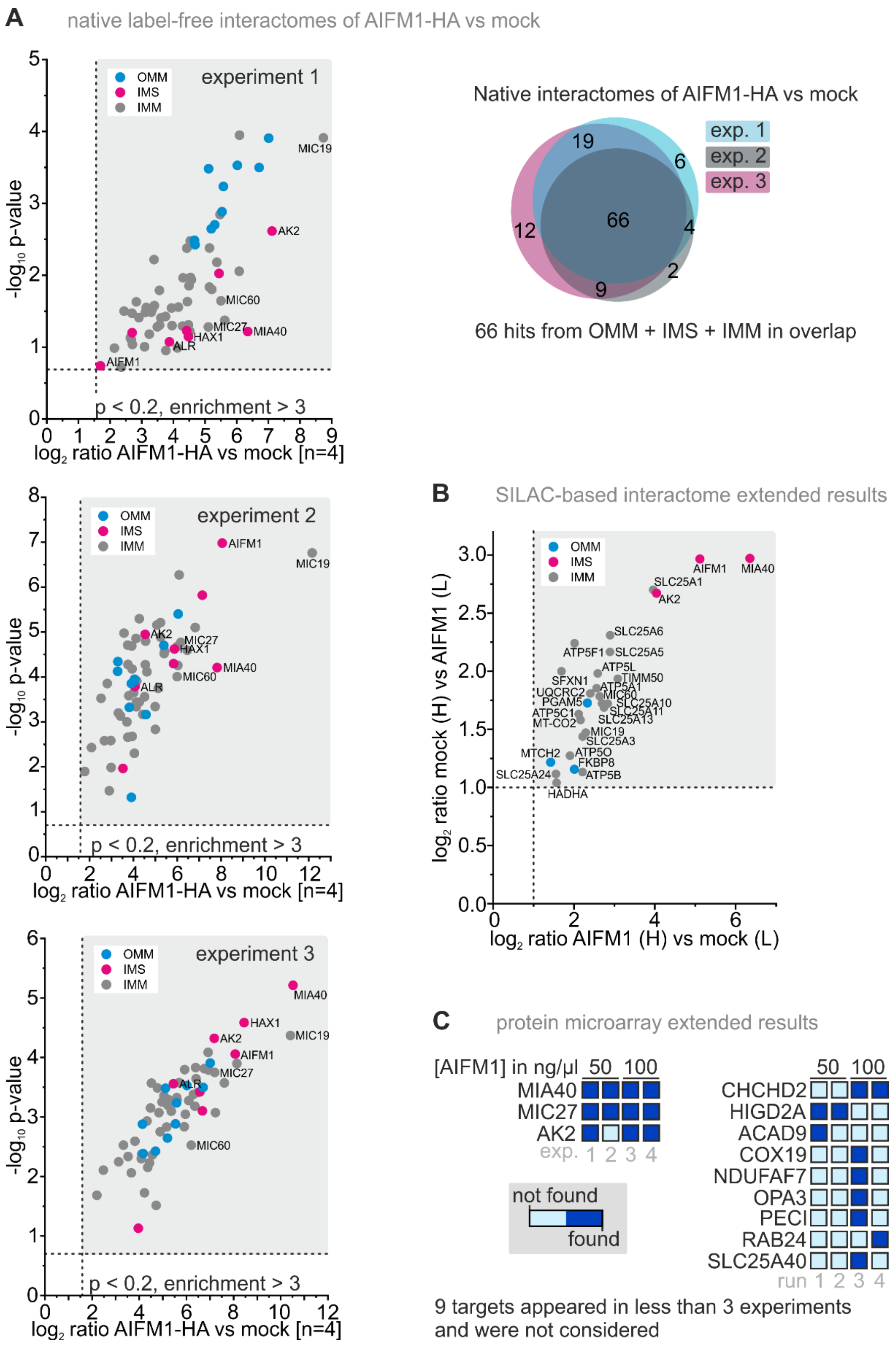

Figure S1: A high-confidence interactome reveals AK2 and MICOS components as novel AIFM1 interaction partners related to Figure 1.

(A) Individual data sets for the three repeats with four biological replicates each shown in **Figure 1C**. The interactomes show considerable overlap leading to the identification of 66 potential interactors of AIFM1-HA.

(B) SILAC-based data set for the experiment shown in **Figure 1D**. We identified 27 potential interactors localized in IMM, IMS and OMM. Notably, the datasets from (A) and (B) do not only show AK2, MIA40, and MICOS components as potential AIFM1 interactors but also members of the SLC25 family (including the ADP/ATP carrier SLC25A5) and the ATPase (including ATP5A1, ATP5B, ATP5F1, ATP5L, and ATP5O)

(C) Extended results for the protein microarray shown in **Figure 1E**. Many different targets were only identified in single experiments. Only AK2, MIA40 and MIC27 were consistently identified.

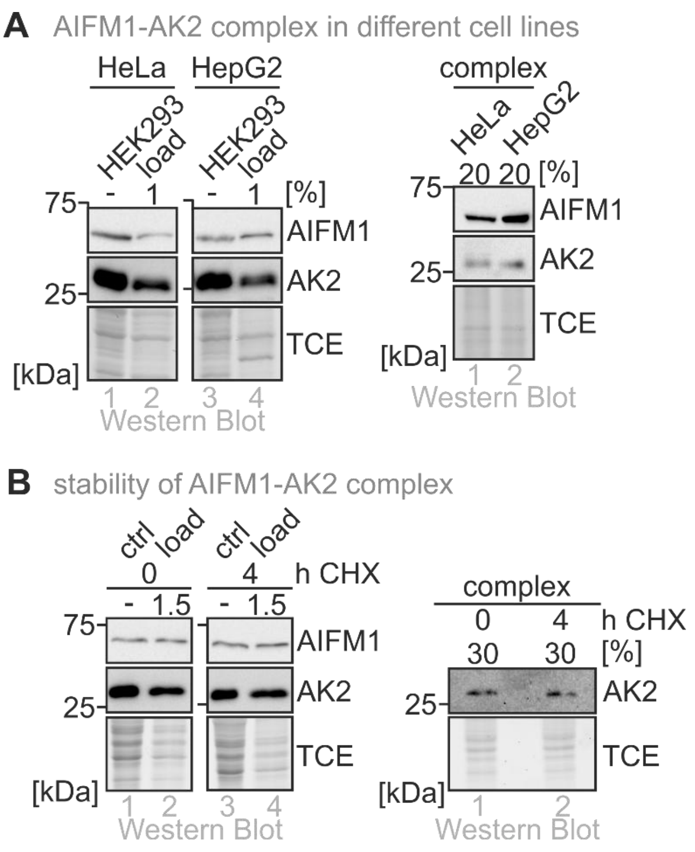

Figure S2: Properties of the AIFM1-AK2 complex related to Figure 2.

(A) The AIFM1-AK2A complex is present in different cell lines. The experiment was performed as in **Figure 2F** in the indicated cell lines.

(B) The AIFM1-AK2A complex is stable. The experiment was performed as in **Figure 2F** except that cells were treated with the translation inhibitor cycloheximide for 4 hours or left untreated. The amounts of AK2A in the AIFM1-AK2A complex do not change during this time indicating a stable complex.

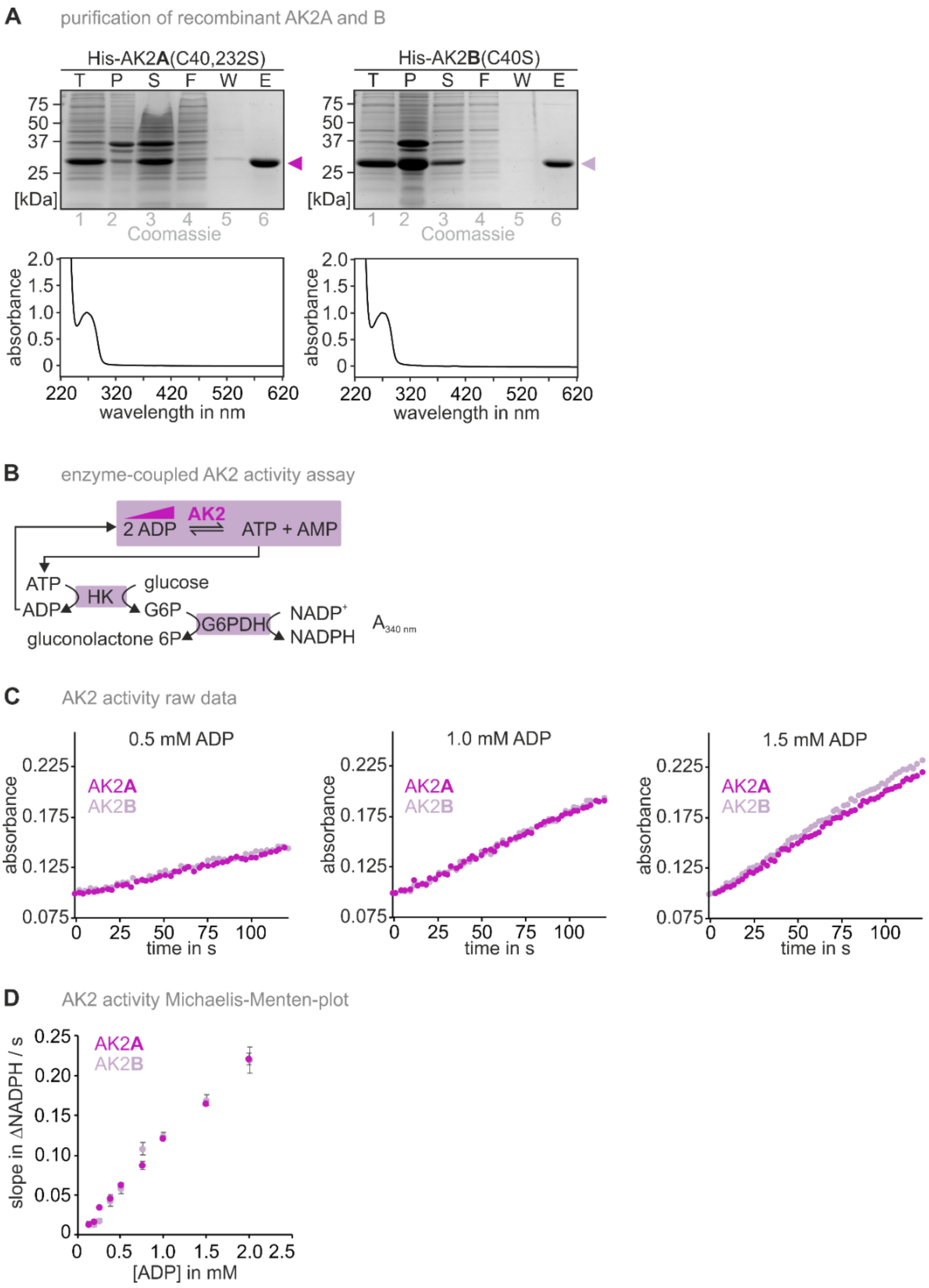

Figure S3: Recombinant AK2 isoform A and B exhibit a similar enzymatic profile related to Figure 2.

(A) Purification of the isoforms His-AK2A and His-AK2B. Both proteins were well-behaved and can be purified in similar amounts and to similar purity. T: total, P: pellet, S: supernatant, F: flow-through, W: wash, E: eluate

(B) AK2 activity assay with varying ADP concentrations. Hexokinase (HK) and glucose-6-phosphate dehydrogenase (G6PDH) couple the reduction of $NADP^+$ to the interconversion of adenine nucleotides. G6P, glucose-6-phosphate

(C,D) Raw data **(C)** and velocity *vs* ADP concentration slope plot **(D)** for the enzyme activities of AK2A and AK2B. The concentration of ADP is titrated. Both isoforms exhibit the same activity towards ADP.

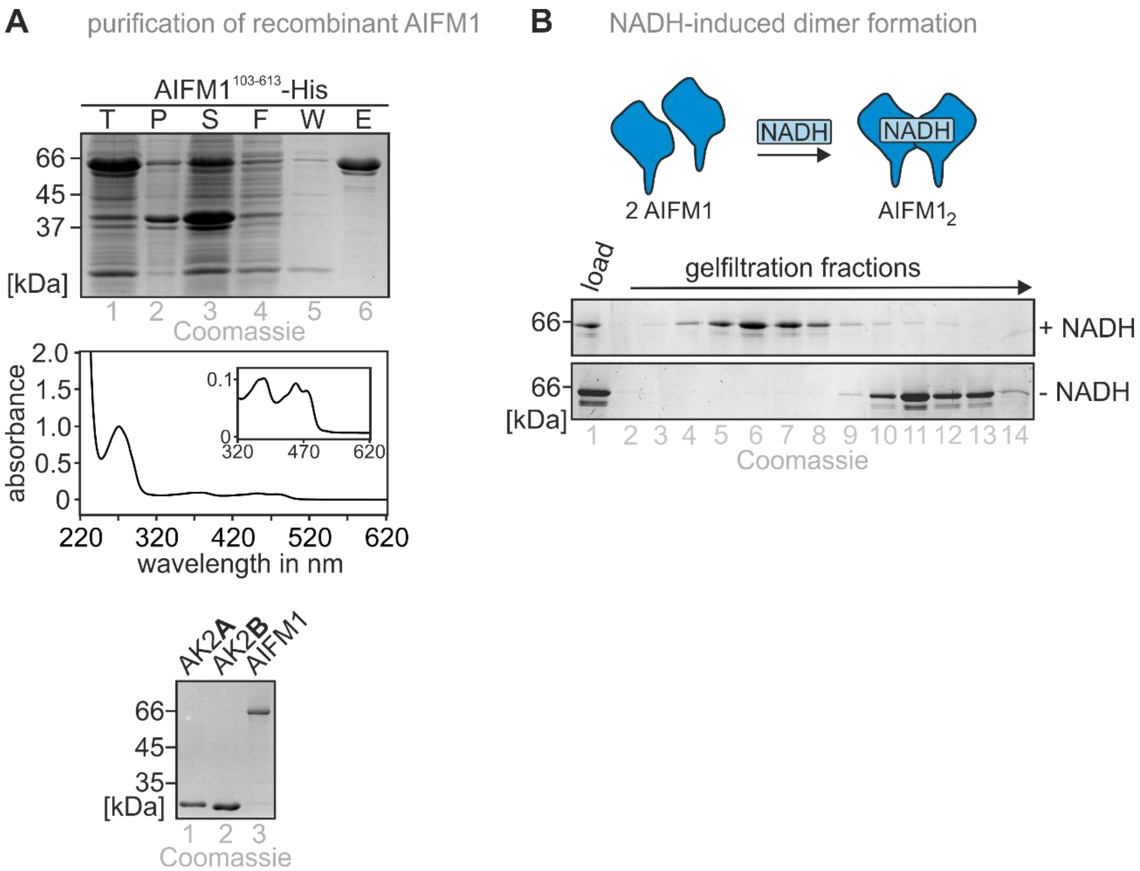

Figure S4: NADH-induced *in vitro* dimerization of recombinant soluble AIFM1 related to Figure 2.

(A) Purification of soluble AIFM1 (AIFM1 103-613). AIFM1, lacking the mitochondrial targeting signal and the transmembrane domain, was purified and contained the FAD cofactor. Purified AK2A and AK2B used in the *in vitro* reconstitution assay of the AIFM1-AK2 complex were loaded for comparison onto the same gel as AIFM1. T: total, P: pellet, S: supernatant, F: flow-through, W: wash, E: eluate

(B) NADH-induced dimer formation of AIFM1. NADH addition leads to rapid AIFM1 dimerization that can be followed by gel filtration. In the absence of NADH, AIFM1 migrates at the height of the monomer.

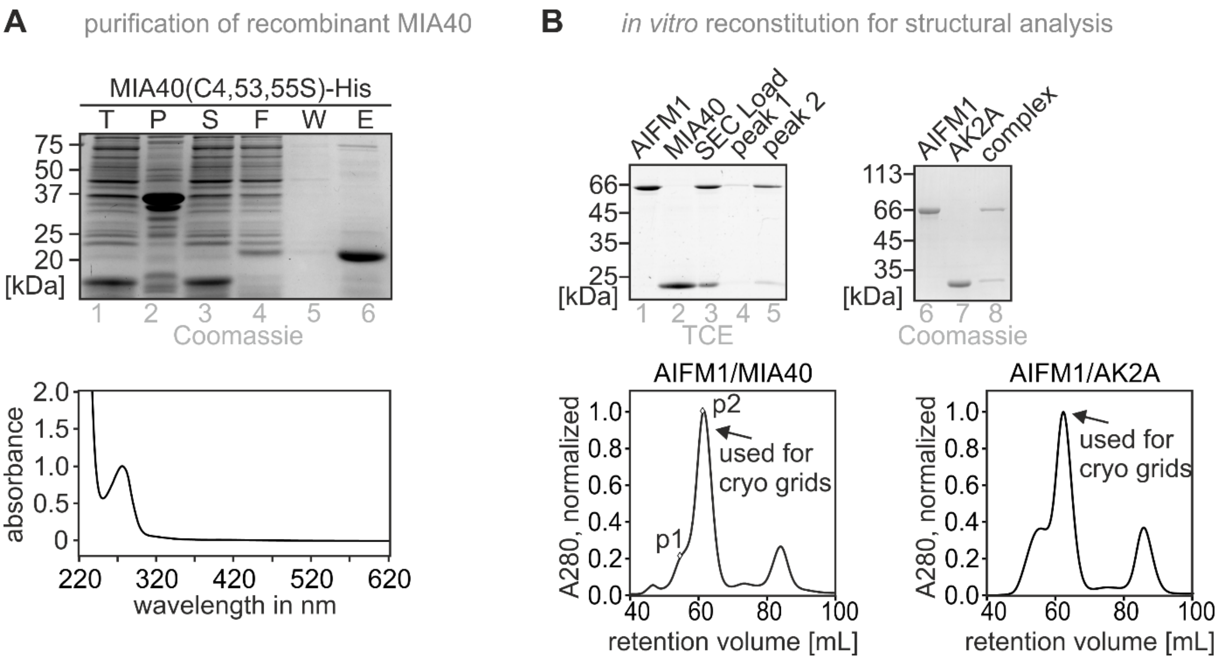

Figure S5: Protein preparation for cryo-EM related to Figure 3.

(A) Purification of the redox-inactive MIA40-C4,53,55S variant. T: total, P: pellet, S: supernatant, F: flow-through, W: wash, E: eluate

(B) *In vitro* reconstitution of the AIFM1-MIA40 and AIFM1-AK2A complexes and isolation of the complexes by gel filtration for cryo-EM.

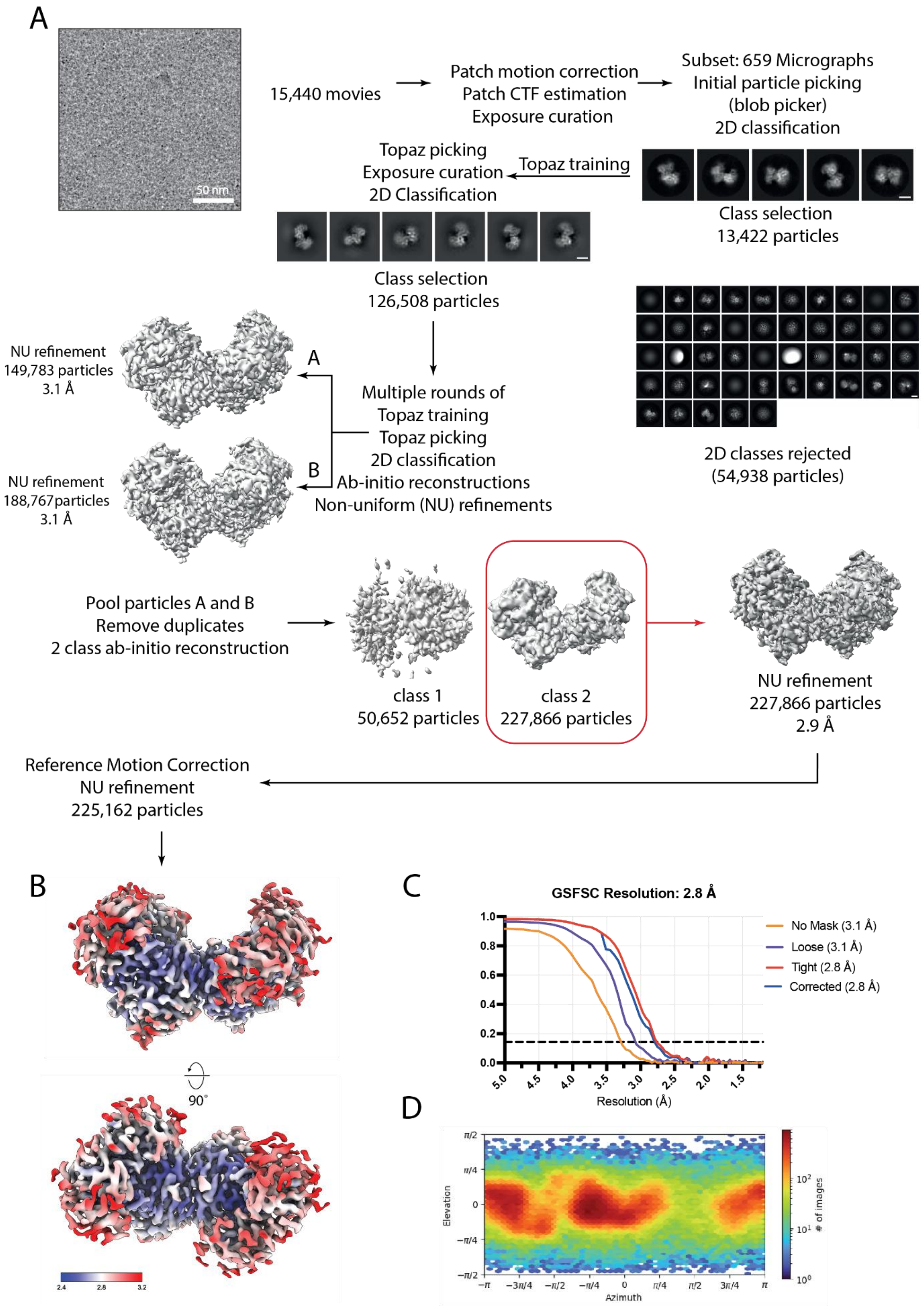

Figure S6: Cryo-EM data processing workflow of the AIFM1 dimer related to Figure 3.

(A) Processing workflow for the AIFM1 dimer. All data processing was performed in cryoSPARC [1]. The first model of the Topaz particle picker [2] was trained with a subset of particles identified by 2D

classification after using the blob picker. Several rounds of Topaz training with particles that gave rise to well-resolved 2D class averages were performed, successively increasing the number and quality of particle picks. Particle subsets were selected based on structural details visible in 2D class averages. *Ab-initio* reconstructions using multiple classes were performed to further remove particles not contributing to well-resolved 3D reconstructions. Similarly, before the last refinement, a new *ab-initio* reconstruction with two classes was performed, sorting out further particles that did not give rise to well-resolved reconstructions. Scale bars in 2D class averages are 5 nm.

(B) Final reconstruction colored according to local resolution, ranging from 2.4 Å (blue) to 3.2 Å (red).

(C) The global resolution determined by the FSC cut-off at 0.143 was 2.8 Å.

(D) Euler angle distribution plot, exported from cryoSPARC.

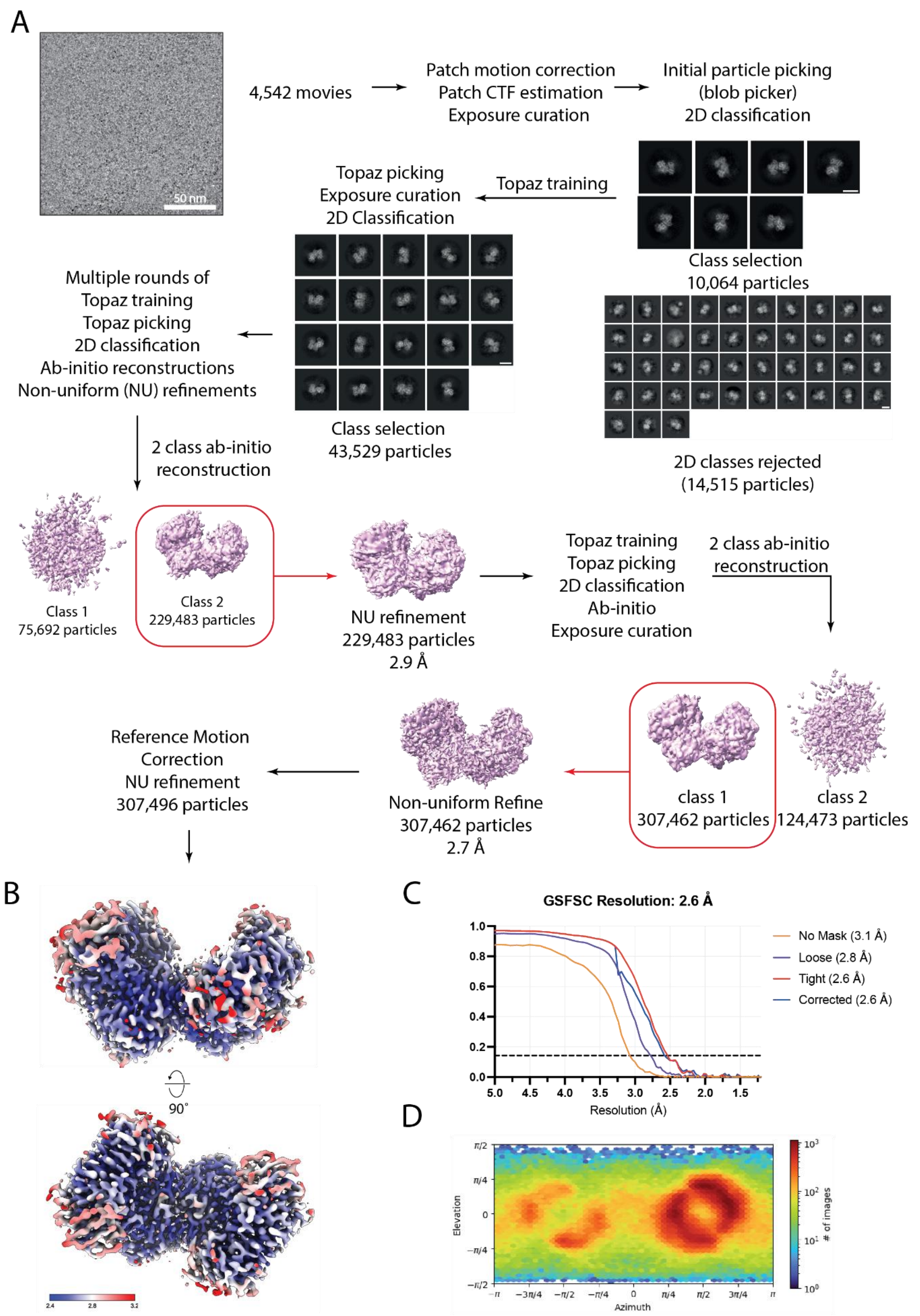

Figure S7: Cryo-EM data processing workflow of the AIFM1-AK2A complex related to Figure 3.

(A) Processing workflow for the AIFM1-AK2A complex. All data processing was performed in cryoSPARC [1]. The first model of the Topaz particle picker was trained with a subset of particles identified

by 2D classification after using the blob picker. Several rounds of Topaz training with particles that gave rise to well-resolved 2D class averages were performed, successively increasing the number and quality of particle picks. Particle subsets were selected on the basis of structural details visible in 2D class averages. *Ab-initio* reconstructions using multiple classes were performed to further remove particles not contributing to well-resolved 3D reconstructions. Similarly, before the last refinement, a new *ab-initio* reconstruction with two classes was performed, sorting out further particles that did not give rise to well-resolved reconstructions. Scale bars in 2D class averages are 5 nm.

(B) Final reconstruction colored according to local resolution, ranging from 2.4 Å (blue) to 3.2 Å (red).

(C) The global resolution determined by the FSC cut-off at 0.143 was 2.6 Å.

(D) Euler angle distribution plot, exported from cryoSPARC.

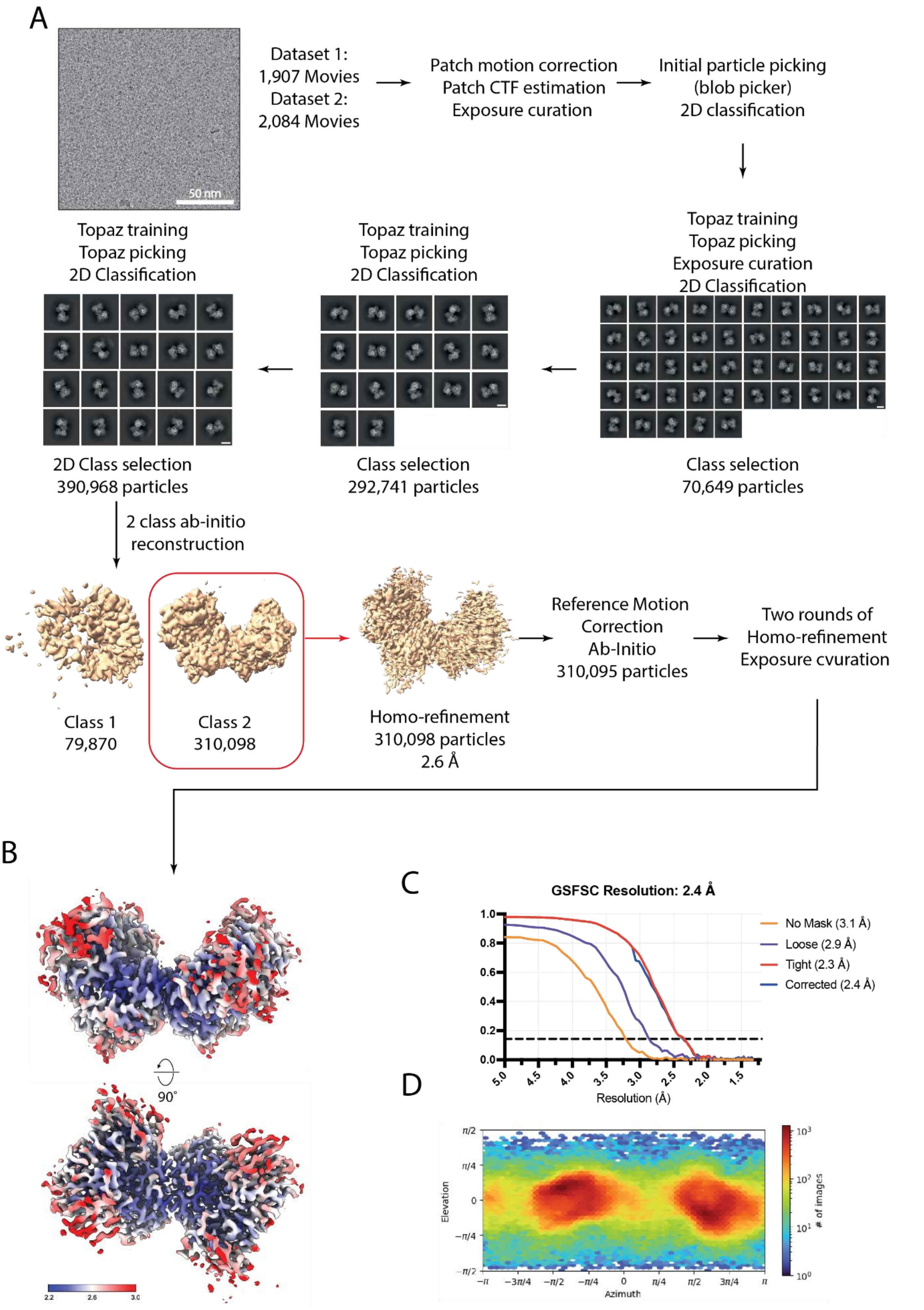

Figure S8: Cryo-EM data processing workflow of the AIFM1-MIA40 complex related to Figure 3.

(A) Processing workflow for the AIFM1-MIA40 complex. All data processing was performed in cryoSPARC [1]. The first model of the Topaz particle picker was trained with a subset of particles identified

by 2D classification after using the blob picker. Several rounds of Topaz training with particles that gave rise to well-resolved 2D class averages were performed, successively increasing the number and quality of particle picks. Particle subsets were selected based on structural details visible in 2D class averages. *Ab-initio* reconstructions using multiple classes were performed to further remove particles not contributing to well-resolved 3D reconstructions. Similarly, before the last refinement, a new *ab-initio* reconstruction with two classes was performed, sorting out further particles that did not give rise to well-resolved reconstructions. Scale bars in 2D class averages are 5 nm.

(B) Final reconstruction colored according to local resolution, ranging from 2.2 Å (blue) to 3.0 Å (red).

(C) The global resolution determined by the FSC cut-off at 0.143 was 2.4 Å.

(D) Euler angle distribution plot, exported from cryoSPARC.

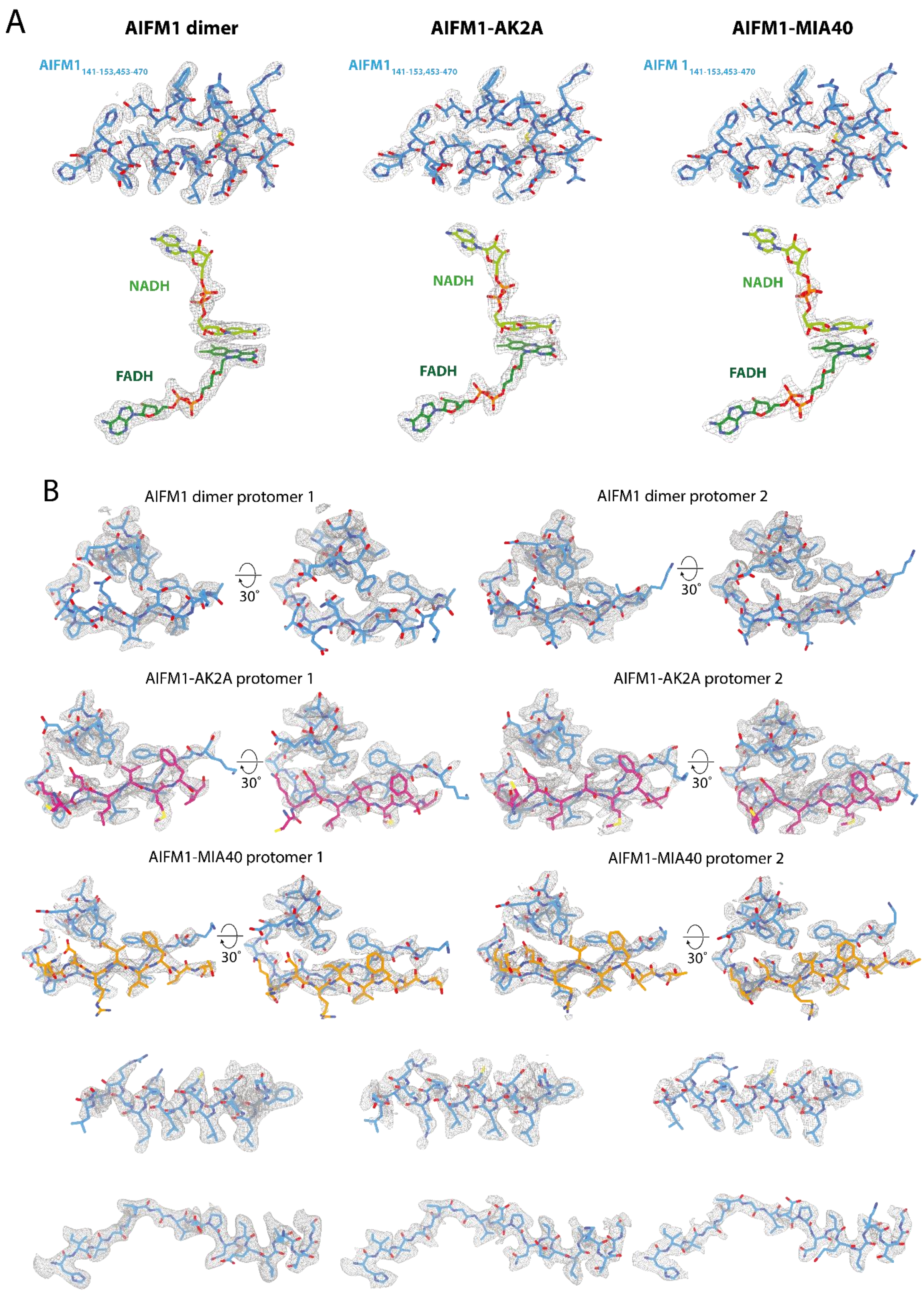

Figure S9: Cryo-EM map quality related to Figure 3.

(**A**) Exemplary cryo-EM densities (grey mesh) and atomic models obtained in this study for AIFM1 (aa 141-153,453-470), top panels, or the NAD and FAD cofactors (bottom panels), of all three structures shown here.

(B) Exemplary cryo-EM densities (grey mesh) and atomic models of the C-terminal domain of AIFM1 (aa 343-349 and 500-510, blue), AK2A (aa 232-239, blue), and MIA40 (aa 8-16, orange), shown for all three structures and both protomers of each dimer.

(C) Exemplary cryo-EM densities (grey mesh) and atomic models of AIFM1 aa 308-327, shown for the AIFM1 dimer (left), AIFM1-AK2A (middle) and AIFM1-MIA40 (right) models.

(D) Exemplary cryo-EM densities (grey mesh) and atomic models of AIFM1 aa 393-411, shown for the AIFM1 dimer (left), AIFM1-AK2A (middle) and AIFM1-MIA40 (right) models.

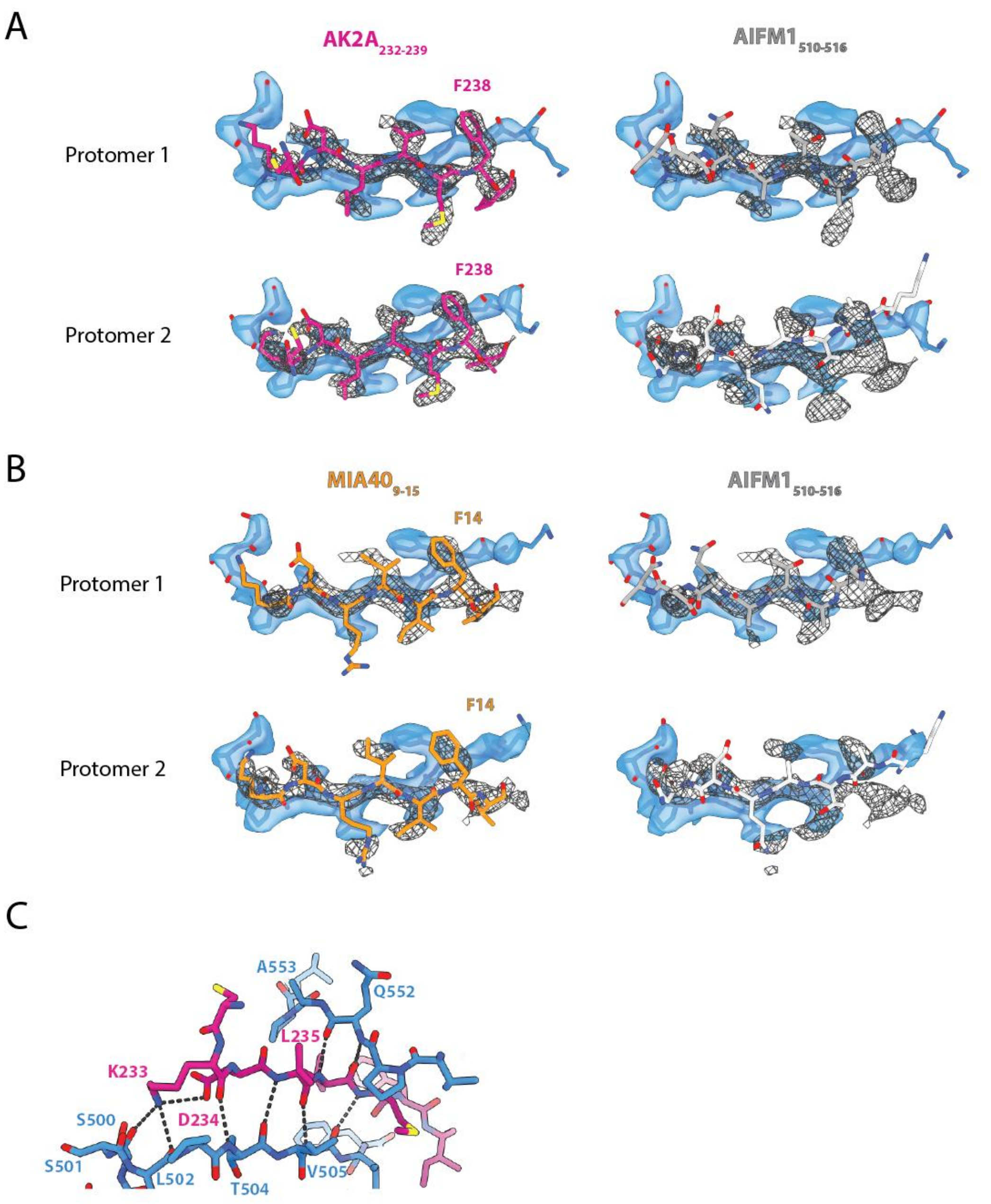

Figure S10: Details of AK2A and MIA40 interactions with AIFM1, unambiguous fit of AK2A and MIA40 related to Figure 3.

(A) Cryo-EM map of the AIFM1-AK2A complex and fitted model for AIFM1 (aa 500-510), (blue, transparent map) and AK2A (aa 232-239), (purple, grey mesh map, left panels) showing the fit with AK2A as opposed to AIFM1 (aa 510-516), (grey model, right panels).

(B) Cryo-EM map of the AIFM1-MIA40 complex and fitted model for AIFM1 (aa 500-510), (blue, transparent map) and MIA40 (aa 9-15), (orange, grey mesh map, left panels) showing the fit with MIA40 as opposed to AIFM1 (aa 510-516), (grey model, right panels).

(C) Details of the interactions of AK2A (purple) with the C-loop of AIFM1 (blue), and hydrogen bonds between the conserved K233 and D234 of AK2A (corresponding to K9 and D10 of MIA40) and AIFM1.

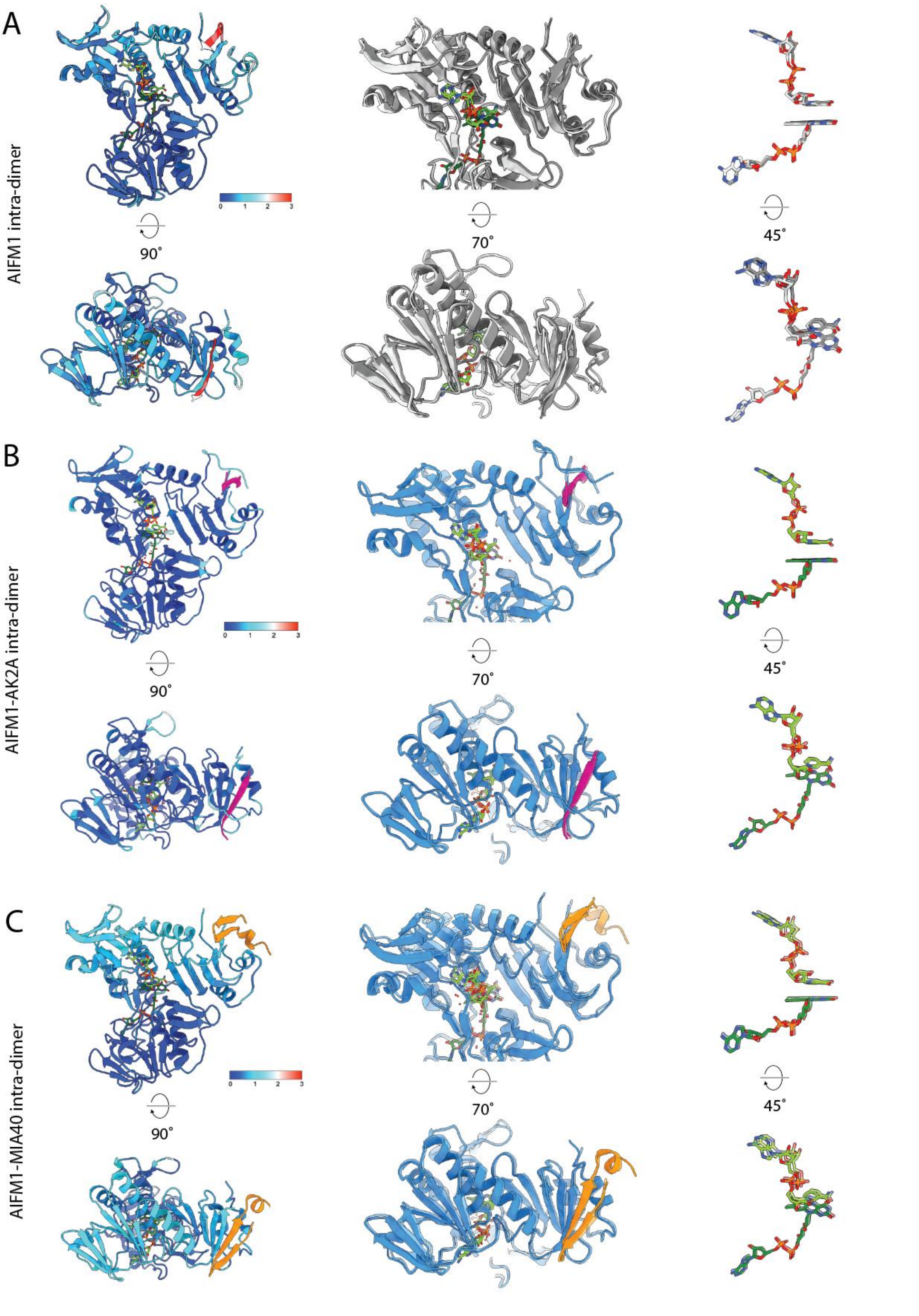

Figure S11: Structural variability between protomers within each AIFM1 dimer of each complex related to Figure 4.

AIFM1 monomers within each complex were superimposed using UCSF ChimeraX at N-terminal residues including the dimer interface (aa 232-257, 404-434 and 440-450). Left panels: models colored

according to the root-mean-square deviation (rmsd) of the Cα atoms. Color code: blue = 0 Å, cyan = 1 Å, white = 2 Å, red = 3 Å. Middle panels: enlarged view of an overlay of both monomer models after alignment, one of the models transparent, to highlight displacement. Right panels: enlarged view of the NAD and FAD cofactors of both monomers after alignment of the models to visualize the direction and extent of variability. **(A)** AIFM1 dimer. **(B)** AIFM1-AK2A complex. **(C)** AIFM1-MIA40 complex.

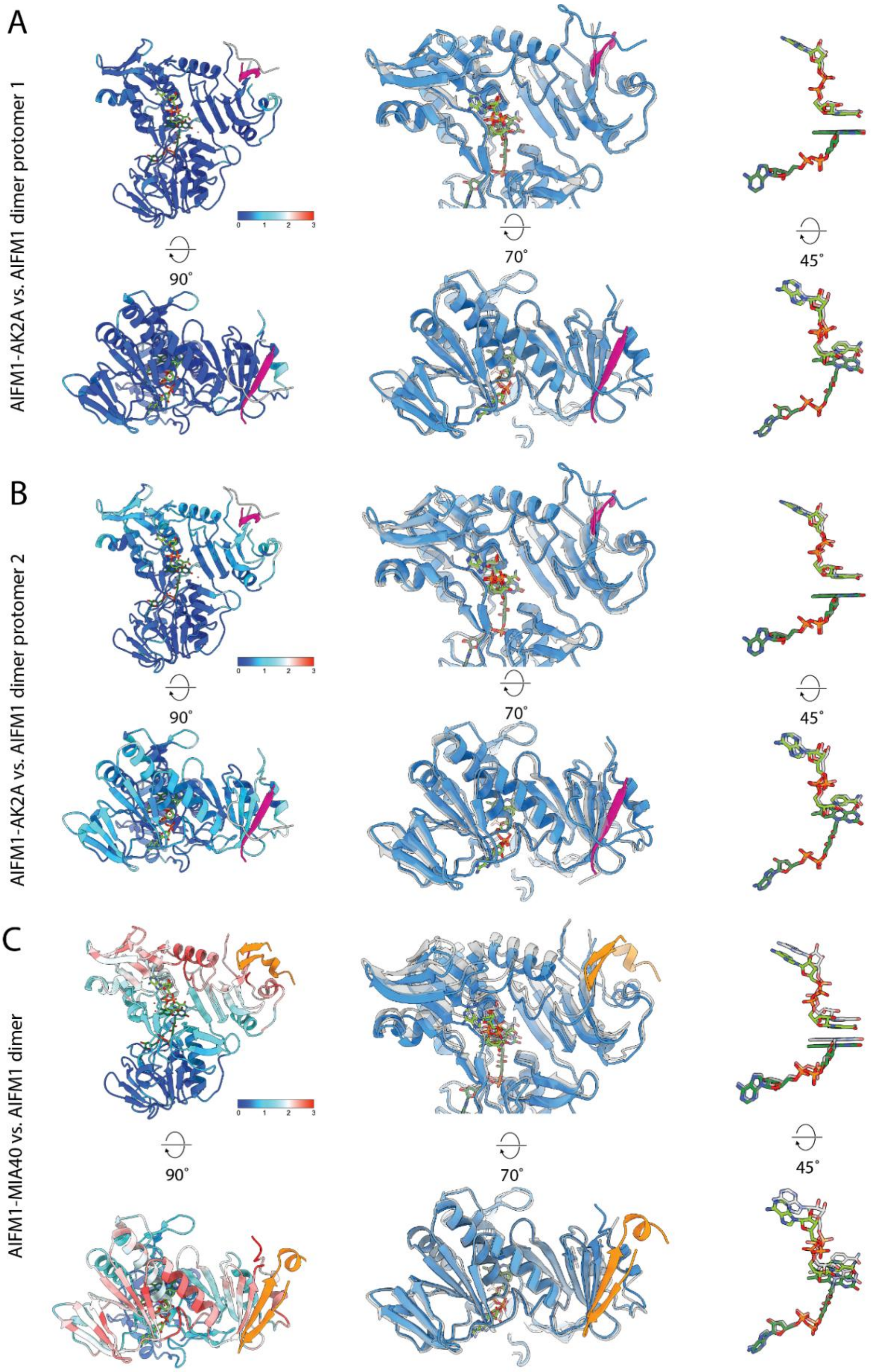

Figure S12: Structural variability between monomers of AIFM1-MIA40 and AIFM1-AK2A complexes as compared to the AIFM dimer related to Figure 4.

Monomers of the AIFM1-AK2A complex (**A, B**) or the AIFM1-MIA40 complex (**C**) were superimposed using UCSF ChimeraX at the N-terminal β-sheets (aa 128-165 and aa 212-261). Left panels: models colored according to the root-mean-square deviation (rmsd) of the Cα atoms. Color code: blue = 0 Å,

cyan = 1 Å, white = 2 Å, red = 3 Å. Middle panels: enlarged view of an overlay of both monomer models after alignment, one of the models transparent, to highlight displacement. Right panels: enlarged view of the NAD and FAD cofactors of both monomers after alignment of the models to visualize the direction and extent of variability. For AIFM1-AK2A, the alignment of one monomer to each of the AIFM1 dimer protomers is shown. For AIFM1-MIA40, only the monomer-to-monomer comparison with the strongest variability is shown.

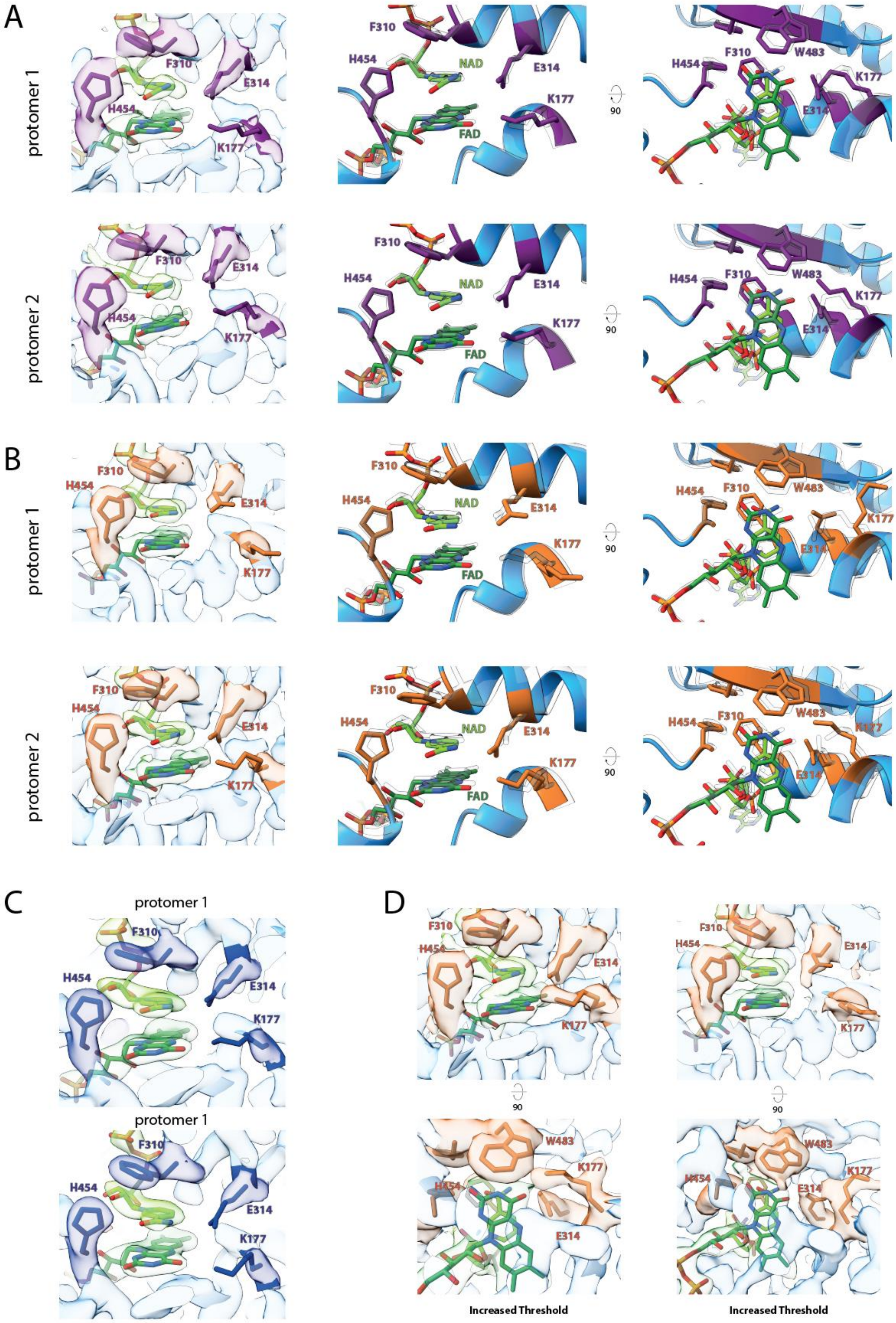

Figure S13: Structural details and conformational changes of the active site of AIFM1 related to Figure 4.

(A) Active site of the two protomers (top and bottom row) of AIFM1-AK2A. Left panel: Local cryo-EM density (semi-transparent) and fitted model, cofactor binding residues shown as stick representation. Middle and right panels: Enlarged views of the AIFM1 active site, residues stabilizing the cofactors shown as stick representation and in purple. The model of the AIFM1 dimer is shown transparent and as an overlay.

(B) Active site of the two protomers (top and bottom row) of MIA40-bound AIFM1. Left panel: Local cryo-EM density (semi-transparent) and fitted model, cofactor binding residues shown as stick representation. Middle and right panels: Enlarged views of the AIFM1 active site, residues stabilizing the cofactors shown as stick representation and in orange. The model of the AIFM1 dimer is shown transparent and as an overlay.

(C) Cryo-EM density of the active site of the AIFM1 dimer of both protomers (top and bottom panels) with the respected atomic model fitted.

(D) Cryo-EM density of the active site of AIFM1-MIA40 of both protomers (left and right panels) shown at increased threshold (bottom panels) to visualize weaker densities indicating flexibility.

A

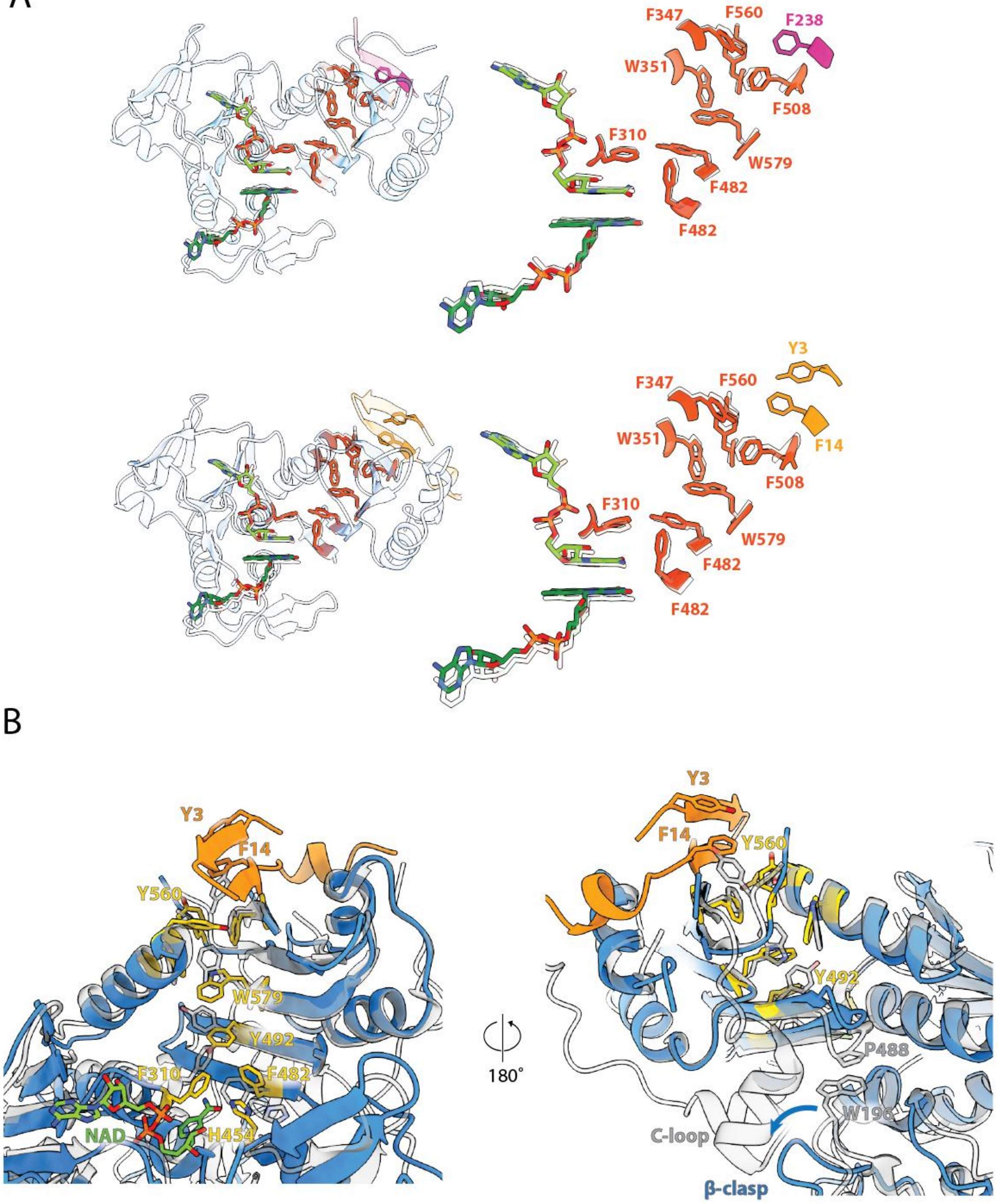

Figure S14: Aromatic tunnel of AIFM1 and conformational stabilization of the aromatic tunnel by AK2A and MIA40 binding related to Figure 4.

(A) Aromatic tunnel of AIFM1. Left panel: aromatic residues of AIFM1 (dark orange), AK2A (top, purple) and MIA40 (bottom, light orange) forming the aromatic tunnel within an AIFM1 monomer, linking the cofactor binding site and protein surface. Right panel: enlarged detail of amino acids and the NAD and FAD cofactors involved. Transparent model: overlay of the AIFM1 dimer lacking AK2A or MIA40 binding.

(B) Structural details of aromatic aa side chains forming the ‘aromatic tunnel’ and the conformational impact of MIA40 binding (orange). Aromatic tunnel residues and the NAD binding H454 are highlighted in yellow. The AIFM1 model in the monomeric, oxidized conformation (PDB 4BV6, [3]) is shown as a grey, transparent overlay.

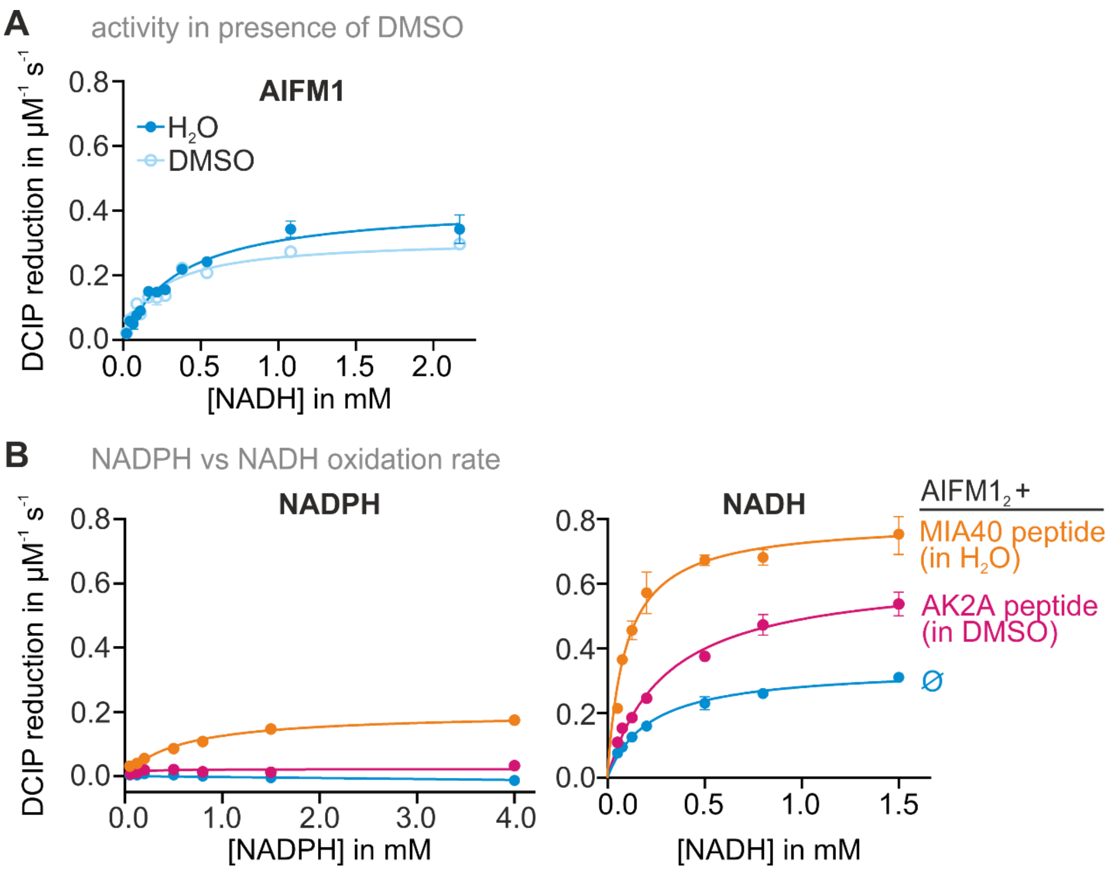

Figure S15: AIFM1 changes its activity upon binding of AK2 or MIA40 related to Figure 4.

(A) Activity of AIFM1 towards NADH and DCIP does not change in the presence of low amounts of DMSO.

(B) Addition of AK2 or MIA40 to AIFM1 increases its activity towards NADH and DCIP. AIFM1 activity towards NADPH is in this assay negligible. Only binding of MIA40 to AIFM1 results in a minor increase of AIFM1 activity. Please observe the differences in the X-axis of the two plots in this panel.

REFERENCES

[S1] Punjani, A., Rubinstein, J.L., Fleet, D.J., and Brubaker, M.A. (2017). cryoSPARC: algorithms for rapid unsupervised cryo-EM structure determination. Nat Methods *14*, 290-296. 10.1038/nmeth.4169.

[S2] Bepler, T., Morin, A., Rapp, M., Brasch, J., Shapiro, L., Noble, A.J., and Berger, B. (2019). Positive-unlabeled convolutional neural networks for particle picking in cryo-electron micrographs. Nat Methods *16*, 1153-1160. 10.1038/s41592-019-0575-8.

[S3] Ferreira, P., Villanueva, R., Martinez-Julvez, M., Herguedas, B., Marcuello, C., Fernandez-Silva, P., Cabon, L., Hermoso, J.A., Lostao, A., Susin, S.A., and Medina, M. (2014). Structural insights into the coenzyme mediated monomer-dimer transition of the pro-apoptotic apoptosis inducing factor. Biochemistry *53*, 4204-4215. 10.1021/bi500343r.
