# Supplementary material for: Interaction with AK2A links AIFM1 to cellular energy metabolism
Source: Mol Cell. Author manuscript; Available in PMC 2025 Jul 30. (PMC7617965; doi:10.1016/j.molcel.2025.05.036)
Supplement: Document S2. [file EMS207342-supplement-Document_S2_.pdf]

Molecular Cell

Article

Interaction with AK2A links AIFM1 to cellular energy metabolism

Graphical abstract

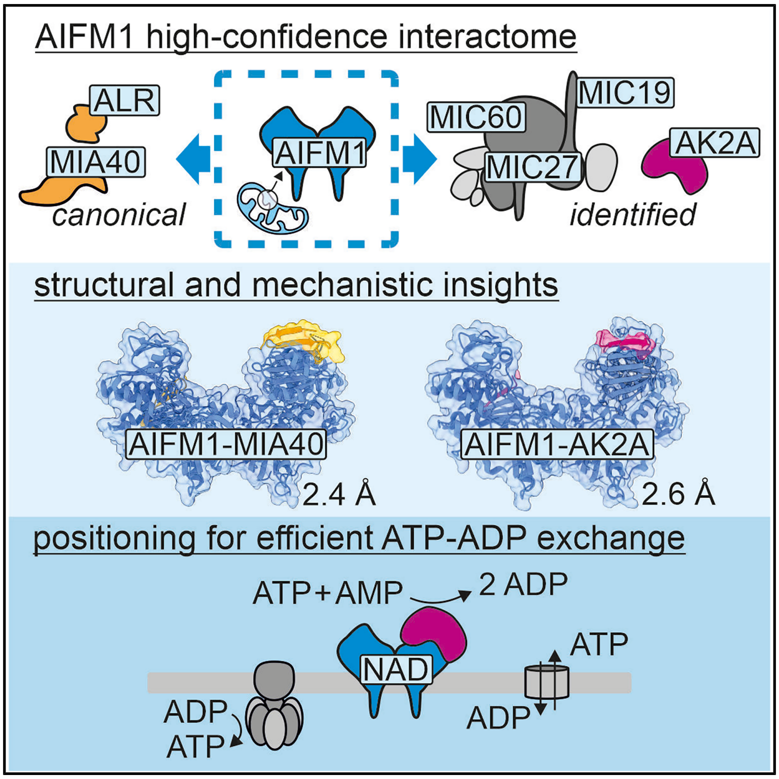

Highlights

- AIFM1 interacts with MICOS subunits, adenylate kinase 2 isoform A (AK2A), and MIA40
- NADH-dependent AIFM1 dimers form stable complexes with AK2A
- AIFM1 dimers interact with AK2A and MIA40 through common structural mechanisms
- An AIFM1/AK2A hub ensures metabolic adaptation

Authors

Robin Alexander Rothemann, Egor Pavlenko, Mrityunjoy Mondal, ..., Daniele Bano, Simon Poepsel, Jan Riemer

Correspondence

spoepsel@uni-koeln.de (S.P.), jan.riemer@uni-koeln.de (J.R.)

In brief

AIFM1 is crucial for mitochondrial function. Here, Rothemann, Pavlenko et al. show that AK2A interacts with NADH-dependent AIFM1 dimers. They demonstrate that this interaction stabilizes AIFM1 dimers and enhances their activity. Given the proximity to ADP/ATP translocases and ATP synthase, the AIFM1-AK2A complex may contribute to metabolic adaptation.

Rothemann et al., 2025, Molecular Cell *85*, 2550–2566
July 3, 2025 © 2025 The Author(s). Published by Elsevier Inc.
https://doi.org/10.1016/j.molcel.2025.05.036

Molecular Cell

Article

Interaction with AK2A links AIFM1 to cellular energy metabolism

Robin Alexander Rothemann,[1,8] Egor Pavlenko,[2,8] Mrityunjoy Mondal,[3] Sarah Gerlich,[1] Pavel Grobushkin,[1] Sebastian Mostert,[1] Julia Racho,[1] Konstantin Weiss,[1] Dylan Stobbe,[1] Katharina Stillger,[4] Kim Lapacz,[1] Silja Lucia Salscheider,[1] Carmelina Petrungaro,[1] Dan Ehninger,[3] Thi Hoang Duong Nguyen,[5] Jörn Dengjel,[6] Ines Neundorf,[4] Daniele Bano,[3] Simon Poepsel,[2,7,8,*] and Jan Riemer[1,7,8,9,*]

[1]Redox Metabolism Group, Institute for Biochemistry, University of Cologne, 50674 Cologne, Germany

[2]Center for Molecular Medicine Cologne (CMMC), Faculty of Medicine and University Hospital, University of Cologne, 50931 Cologne, Germany

[3]German Center for Neurodegenerative Diseases (DZNE), 53127 Bonn, Germany

[4]Peptide Biochemistry Group, Institute for Biochemistry, University of Cologne, 50674 Cologne, Germany

[5]Medical Research Council Laboratory of Molecular Biology, Cambridge CB20QH, UK

[6]Department of Biology, University of Fribourg, 1700 Fribourg, Switzerland

[7]Cologne Excellence Cluster on Cellular Stress Responses in Aging-Associated Diseases (CECAD), University of Cologne, 50931 Cologne, Germany

[8]These authors contributed equally

[9]Lead contact

*Correspondence: spoepsel@uni-koeln.de (S.P.), jan.riemer@uni-koeln.de (J.R.)

https://doi.org/10.1016/j.molcel.2025.05.036

SUMMARY

Apoptosis-inducing factor 1 (AIFM1) is a flavoprotein essential for mitochondrial function and biogenesis. Its interaction with MIA40/CHCHD4, the central component of the mitochondrial disulfide relay, accounts for some, but not all, aspects of AIFM1 function. We provide a high-confidence AIFM1 interactome that elucidates functional partners within the mitochondrial intermembrane space. We found that AIFM1 binding to adenylate kinase 2 (AK2), an essential enzyme that maintains cellular adenine nucleotide pools, depends on the AK2 C-terminal domain. High-resolution cryoelectron microscopy (cryo-EM) and biochemical analyses showed that both MIA40 and AK2A bind the AIFM1 C-terminal β-sheet domain. Their binding enhances NADH oxidoreductase activity by locking an active dimer conformation and, in the case of MIA40, affecting the cofactor-binding site. The AIFM1-AK2A interaction is important during mitochondrial respiration because AIFM1 serves as a recruiting hub within the IMS, regulating mitochondrial bioenergetic output by creating hotspots of metabolic enzymes.

INTRODUCTION

Apoptosis-inducing factor mitochondrial 1 (AIFM1) is a NADH-binding flavin adenine dinucleotide (FAD)-dependent oxidoreductase in the mitochondrial intermembrane space (IMS). It plays key roles in mitochondrial function, respiratory chain maintenance, redox control, and non-caspase-dependent cell death.[1–5] Disease-causing AIFM1 mutations in humans and AIFM1 knockdown or knockout (KO) in mice impair mitochondrial bioenergetics, causing neurological disorders, muscle atrophy, and cardiomyopathy.[4,6–11] AIFM1 contributes to the biogenesis of the respiratory chain complex,[10–15] partly through interacting with the mitochondrial disulfide relay system.[3,4,16–21] It facilitates the import and the activity of MIA40 (also CHCHD4), a key factor for mitochondrial protein import and folding.[13–15,22–24] AIFM1 loss alters MIA40-dependent oxidative folding, while MIA40 overexpression can partially compensate.[13–15]

AIFM1 senses IMS NADH levels. Its oxidoreductase activity is facilitated by two Rossman fold FAD- and NADH-binding domains (Figure 1A).[25] Upon NADH binding, an air-stable $FADH_2$-NAD^+ charge-transfer complex (CTC) forms,[26–28] inducing AIFM1 dimerization and the release of a C-terminal regulatory segment termed C-loop.[26,29–31] The NAD-binding residues H454 and F310 of AIFM1 re-arrange to allosterically transmit NADH-dependent structural changes to the AIFM1 dimerization interface, promoting dimerization. The concurrent re-arrangement of aromatic side chains to form an "aromatic tunnel" transmits structural changes to the C-terminal domain. Ultimately, the "β-hairpin" releases the C-loop to open a binding site of electron acceptors and a second NADH molecule.[29,31] NADH levels in the IMS under non-stress conditions have been suggested to drive

 © 2025 The Author(s). Published by Elsevier Inc.
This is an open access article under the CC BY license (http://creativecommons.org/licenses/by/4.0/).

CellPress
OPEN ACCESS

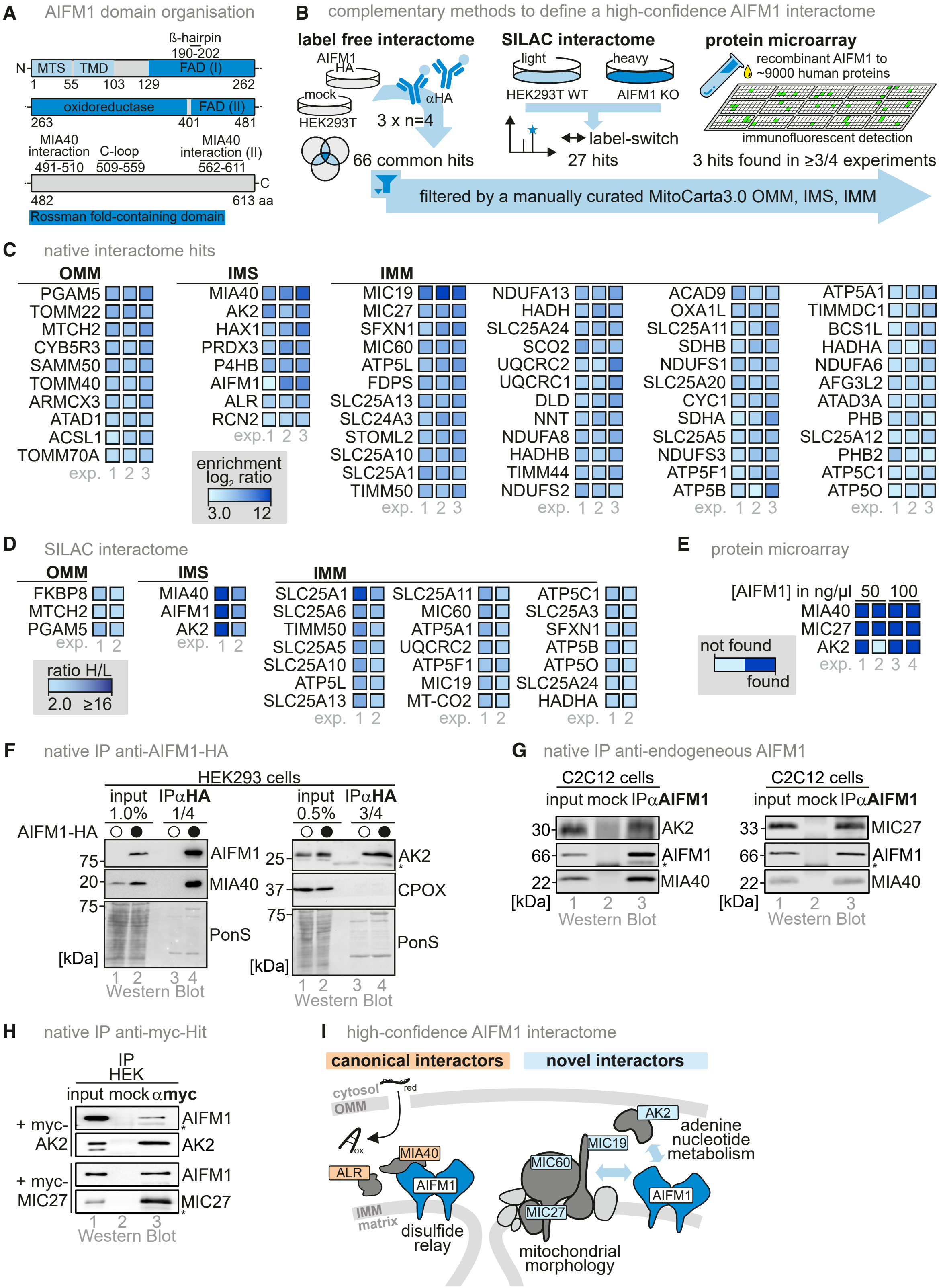

(legend on next page)

AIFM1 dimerization, whereas NADH deficiency, e.g., during starvation, may favor monomeric AIFM1.[1,15,30]

AIFM1 dimerization is required for MIA40 import and binding and is thus critical for MIA40 function.[13,15,23,32] The unstructured N-terminal region of MIA40 mediates the interaction with AIFM1.[13,15] A recent crystal structure revealed the interaction of a MIA40 β-hairpin (amino acids, aa 3–15) with the AIFM1 C-terminal region (aa 491–510 and 562–611) by parallel β-strand complementation.[32] These results suggest that C-loop displacement is required to establish the MIA40-binding site.

Given the pleiotropic effects of pathogenic AIFM1 mutations on oxidative phosphorylation (OXPHOS),[1,4,20,33] we reasoned that AIFM1 deficiency may cause metabolic phenotypes by impacting other binding partners. Here, we provide a high-confidence AIFM1 interactome that identified adenylate kinase 2 isoform A (AK2A) and mitochondrial contact site and cristae organizing system (MICOS) complex components. Although MICOS establishes and maintains mitochondrial morphology, AK2 controls the adenine nucleotide balance in the IMS, which is essential for mitochondrial ADP/ATP carrier function (SLC25A4-A6).[34–37] In organs with high energy demand such as the heart, AK2 accounts for almost half of the cellular AK activity, emphasizing its critical role in respiration.[38,39] AK2 loss leads to impaired mitochondrial function,[40,41] hampers induction of the endoplasmic reticulum unfolded protein response,[40] and sensitizes cells to induction of apoptosis.[42–44] AK2 KO in mice is embryonic lethal (E7.5),[45] whereas human patients suffer from an autosomal recessive form of severe combined immunodeficiency named reticular dysgenesis.[46,47]

Single-particle cryoelectron microscopy (cryo-EM) revealed that MIA40 and AK2A bind the same site in AIFM1, enhancing dimer stability and NADH oxidase activity of AIFM1 at physiological NADH concentrations. The AIFM1-AK2A interaction likely supports metabolic adaptation from fermentative to respiratory carbon sources by positioning AK2A near ADP/ATP carriers.

RESULTS

AK2 and MICOS components are AIFM1 interaction partners

We employed complementary approaches to obtain a high-confidence interactome of AIFM1 (Figure 1B). First, we performed native immunoprecipitation (IP) from HEK293 cells stably expressing C-terminally HA-tagged AIFM1, followed by quantitative label-free proteomic analysis. We performed three independent experiments with four biological replicates each and identified 66 proteins with IMS localization or domains facing the IMS as potential interaction partners of AIFM1 (Figures 1B, 1C, and S1A). We then employed a similar but SILAC (stable isotope labeling with aa in cell culture)-based approach, which allows quantification of protein enrichment (Figures 1B, 1D, and S1B), and identified 27 potential interaction partners of AIFM1. We complemented these analyses by an unbiased *in vitro* protein-protein profiling using microchips containing purified recombinant human proteins and incubated them with purified AIFM1 (soluble AIFM1 lacking the transmembrane domain[25]). Antibody detection of AIFM1 revealed three proteins that fulfilled our stringent selection criteria for a potential interaction with AIFM1 (Figures 1B, 1E, and S1C).

By integrating these datasets, we retrieved MIA40, AK2, and MICOS components (MIC27 in all three, MIC19 and MIC60 in two approaches) as common AIFM1 interactors. MIA40 was previously identified to interact with AIFM1.[13–15,22] We verified interaction partners by IP of AIFM1-HA (Figure 1F) and endogenous AIFM1 (Figure 1G). Reciprocal myc-AK2 and myc-MIC27 IPs confirmed their interaction (Figure 1H). Together with the identification of AK2 and MICOS components as AIFM1 interactors, we established a possible link to energy metabolism and the formation and maintenance of mitochondrial morphology (Figure 1I).

The C-terminal region in the isoform AK2A is important for AIFM1 interaction

In gel filtration experiments, we found almost all cellular AIFM1 as well as MIA40 in complexes with an apparent molecular

Figure 1. A high-confidence interactome of AIFM1 reveals AK2 and MICOS components as interaction partners

(A) Domain layout of AIFM1. The two Rossman folds for FAD and NADH binding encompass aa 129–170, 203–261, and 403–479, and 171–202 and 265–399, respectively. The C-loop includes aa 509–559 and the β-hairpin aa 190–202.

(B) Approaches to determine a high-confidence AIFM1 interactome. Three approaches were employed: native immunoprecipitation (IP) of AIFM1-HA followed by label-free proteomics, a SILAC-labeling-based approach, and a native protein-protein profiling microarray approach.

(C) Interaction partners of AIFM1-HA from native label-free co-immunoprecipitation (coIP) experiments. The experiment was reproduced three times with 4 biological replicates rendering the results as indicative for 12 coIP experiments. Fold enrichment in the AIFM1-HA IP was plotted for significant hits of the experimental repeats. A hit is significantly enriched in all three experimental repeats and localizes to mitochondrial inner (IMM) membranes, outer (OMM) membranes, or IMS. $N = 12$ biological replicates, an unpaired one-sample two-sided Student's t test was applied ($p < 0.2$, $\log_2$ enrichment > 3).

(D) Interaction partners of AIFM1-HA from native SILAC-based coIP experiments. The experiment was reproduced with inverted isotope labeling. Proteins were counted as hits if at least two peptides per protein were enriched more than 2-fold and the proteins have a localization in the IMS, IMM, or OMM. $N = 2$ biological replicates.

(E) Identification of AIFM1 interaction partners by protein-protein microarray (Data S1). 12 putative AIFM1-interacting partners localizing to IMM, OMM, and IMS were detected; 3 of them were common candidates in at least three out of four replicates. $N = 4$ biological replicates (2 with 50 and 2 with 100 ng/μl).

(F) Test for interaction with identified hits by AIFM1-HA-IP. Immunoblot analyses were performed against AK2, MIA40, and HA, and, as a non-interacting control, the IMS protein CPOX. PonS, ponceau staining; asterisk, antibody chains.

(G) Test for interaction with identified hits by IP of endogenous AIFM1. Immunoblot analyses were performed against AK2, MIC27, MIA40, and AIFM1. Asterisk, antibody chains.

(H) IP of myc-tagged AK2 and MIC27 to test for interaction with AIFM1. HEK293 cells were transiently transfected with plasmids expressing myc-AK2 or myc-MIC27. Cells were lysed under native conditions, and myc-AK2 and myc-MIC27 were precipitated using myc-antibody beads. Immunoblot analyses were performed against AK2, MIC27, and AIFM1. Asterisk, antibody chains.

(I) MICOS subunits and AK2 are confirmed high-confidence interaction partners of AIFM1, linking AIFM1 to cellular metabolism and mitochondrial morphology.

CellPress
OPEN ACCESS

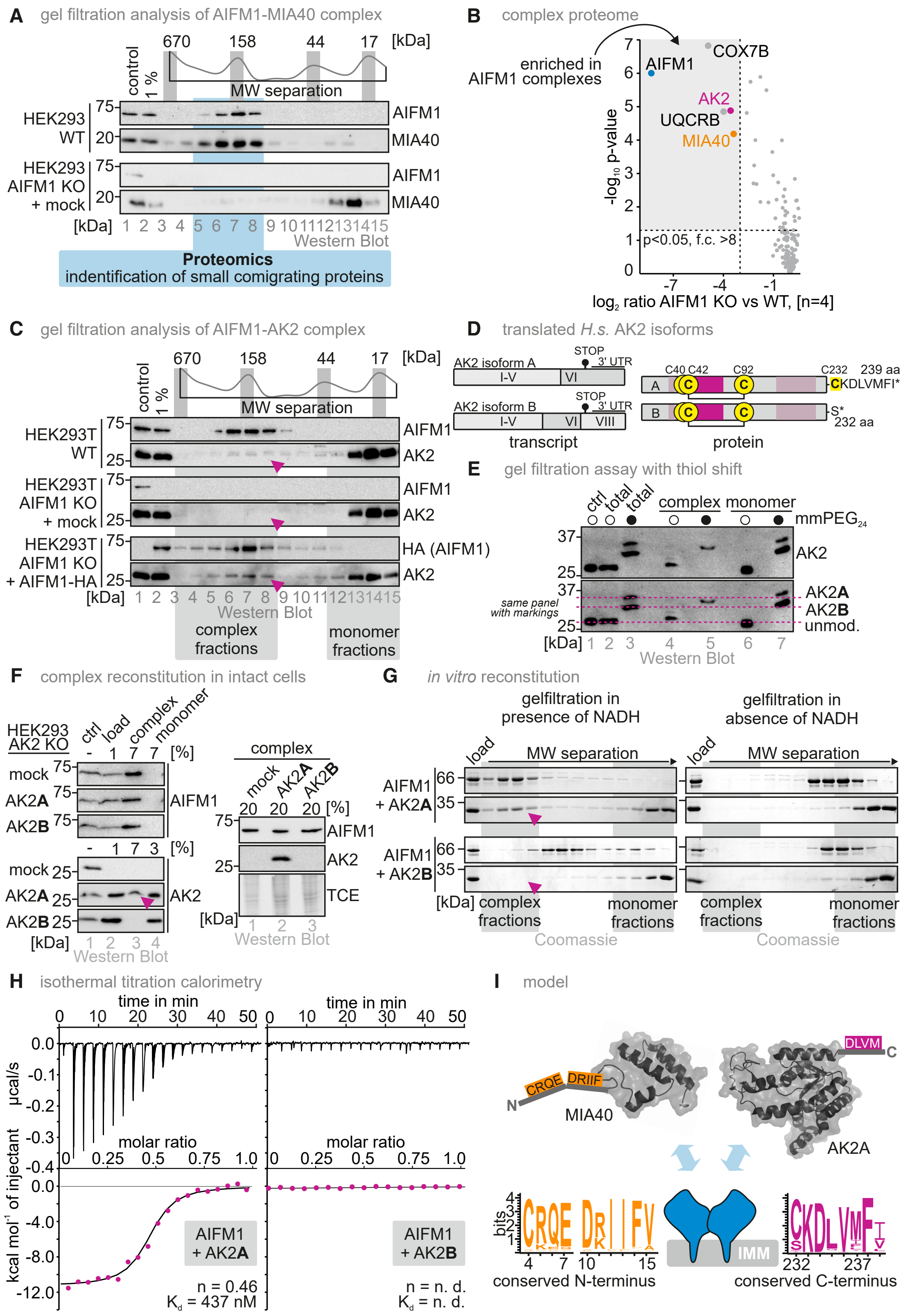

(legend on next page)

weight (MW) between 100 and 200 kDa (Figure 2A, Salscheider et al.[15]). Deletion of AIFM1 resulted in a shift of MIA40 to lower MW fractions (Figure 2A, Salscheider et al.[15]). Because we expected a similar MW shift for other AIFM1-interacting partners, we subjected the high MW AIFM1 complex fractions to proteomic analysis (Figure 2A). AIFM1, MIA40, UQCRB, COX7B, and AK2 were detected in the AIFM1 complex fractions in the wild-type (WT) but not the AIFM1 KO cell lysates (Figure 2B). Importantly, this complementary approach supports a stable AIFM1-AK2 complex that persists during gel filtration. We further confirmed this complex in WT, AIFM1 KO, and AIFM1 KO cells complemented with AIFM1-HA using gel filtration and immunoblotting (Figures 2C and S2A). This complex persists upon cycloheximide treatment for 4 h, indicating that it is stable and long-lived, similar to the AIFM1-MIA40 complex (Figure S2B).

Of note, gel filtration is unsuitable for investigating MICOS-AIFM1 interactions because the >700 kDa MW of a mature MICOS-AIFM1 complex would prevent the detection of the relatively small shift upon loss of AIFM1. Given the impact of AIFM1 deletion on cellular metabolism and the robust identification of AK2 as an AIFM1 interaction partner, we performed a detailed mechanistic analysis of the AIFM1-AK2 interaction.

AK2 isoforms AK2A and AK2B differ only in their C-terminal residues. Although AK2A is 239 aa in length, AK2B ends at S232 (Figure 2D). The enzymatically active domains do not differ and, consequently, the purified proteins have similar activities (Figure S3). To experimentally distinguish AK2 isoforms, we made use of a cysteine in AK2A (C232) that is absent in AK2B and performed a thiol shift assay of the endogenous AK2 comigrating with AIFM1 during gel filtration. The covalent attachment of the maleimide mmPEG24 to free cysteines is detectable as a shift on SDS-PAGE. We detected only AK2A in the high MW fractions upon gel filtration (Figure 2E). Accordingly, in AK2 KO cells expressing AK2A or AK2B, only AK2A, but not AK2B, interacted with AIFM1, as demonstrated by gel filtration (Figure 2F).

We next tested the direct interaction between AIFM1 and AK2 isoforms *in vitro* using recombinant purified AK2A and B and AIFM1 (Figures S3 and S4). Gel filtration showed a NADH-dependent comigration of AK2A, but not AK2B, with AIFM1, suggesting an NADH- and dimerization-dependent interaction similar to AIFM1-MIA40[13,15,32,48] (Figure 2G and S4). We assessed binding between purified AK2 and AIFM1 using isothermal calorimetry (ITC), confirming that only AK2A interacts with AIFM1 (Figure 2H), showing a K_d of 437 nM and a binding stoichiometry of 0.46, indicative of one AK2A and two AIFM1 molecules per complex. The binding is slightly weaker compared with MIA40 (K_d = 180 nM) but adhered to the same stoichiometry.[15] Collectively, our findings show that AK2A interacts with AIFM1 and that the last seven aa, forming a conserved region among AK2A homologs, mediate this interaction (conserved are, in particular, K233, D234, V236, and F238; Figure 2I).

Structures of the AIFM1-AK2A and AIFM1-MIA40 complexes reveal a shared binding site

We solved three structures of human soluble AIFM1 (aa 103–613) by cryo-EM: the dimer (1) without interactors (termed “AIFM1 dimer”), (2) bound to AK2A, or (3) to MIA40 (Figure S5). We obtained reconstructions for the AIFM1 dimer and AIFM1-AK2A and AIFM-MIA40 complexes at global resolutions of 2.8, 2.6, and 2.4 Å, respectively (Figures S6–S9; Table 1). We built atomic

Figure 2. Using its conserved C terminus, the isoform AK2A forms a stable complex with the AIFM1 dimer

(A) Proteomic analysis after complex separation by gel filtration, assessing proteins depleted of the AIFM1-MIA40 complex fractions in AIFM1 KO cells. Endogenous AIFM1 and endogenous MIA40 migrated in a complex with a size of around 150 kDa (as judged by comparison to protein markers: thyroglobulin, 670 kDa; γ-globulin, 158 kDa; ovalbumin, 44 kDa; and myoglobulin, 17 kDa). Absence of AIFM1 resulted in the migration of MIA40 at the height of monomeric MIA40. Fractions of the complex region (light blue) were submitted to quantitative label-free proteomic analysis.

(B) Results of the gel-filtration-coupled proteomics experiment. As expected, MIA40 and AIFM1 are strongly depleted in AIFM1 KO cells at the height of the complex. Applying stringent parameters (fold change > 8) results in the identification of three additional proteins that might potentially reside in the AIFM1-MIA40 complex, AK2, COX7B, and UQCRB. $N = 4$ biological replicates, an unpaired one-sample two-sided Student’s t test was applied ($p < 0.05$, fold change > 8).

(C) Gel filtration analysis of WT, AIFM1 KO, and AIFM1 KO cells complemented with AIFM1-HA HEK293 cells. The majority of the highly abundant AK2 migrated at its monomeric mass. About 5% of the protein was found reproducibly in the same fractions as the MIA40-AIFM1 complex (purple arrowhead). This higher molecular weight fraction of AK2 disappeared upon loss of AIFM1 and reappeared after reintroduction of AIFM1-HA. For better representation, we often pooled in subsequent experiments multiple samples that represented either the complex or the monomer into one common "complex" or "monomer" fraction, respectively.

(D) Human AK2 is present in multiple splice forms. Most prominent are AK2A and AK2B that, as proteins, only differ in the last 8 aa. The remainder of the proteins contain the important sites for activity (P loop, lid, and NMP-binding domain) and also the structural disulfide bond. AK2B stops after 232 aa residues with S232, whereas AK2A stops after 239 aa. Instead of S232, AK2A bears C232. This additional cysteine residue in AK2A (total of 4 cysteines) compared with AK2B (3 cysteines) can aid in distinguishing these two very similar isoforms of the protein.

(E) Gel filtration assay of HEK293 cells coupled to thiol shift assay. Gel filtration fractions were subjected to thiol shift analysis and subsequently analyzed by SDS-PAGE and immunoblotting. The treatment with mmPEG24 modifies all 4 cysteines in AK2A and all 3 cysteines in AK2B. The modification of one additional cysteine in AK2A leads to slower migration of AK2A on SDS-PAGE. The majority of cellular AK2 appears to be AK2B, but it is AK2A that comigrates with AIFM1 in the higher MW complex. Complex and monomer fractions are presented.

(F) Gel filtration assay of HEK293 cells depleted of AK2 (AK2 knockout and AK2 KO) complemented with an empty vector (Mock), AK2A, or AK2B. Gel filtration fractions of the region containing monomeric AK2 and the region containing AK2 in complex were separately pooled and analyzed by SDS-PAGE and immunoblotting. Only AK2A can be detected in the complex fraction.

(G) *In vitro* reconstitution of the AK2A-AIFM1 complex and analysis by gel filtration. Purified AIFM1 was incubated with purified AK2A or AK2B in the presence or absence of NADH. Formation of a higher molecular weight (MW) complex of AIFM1 and AK2 is only detectable in the presence of NADH when AK2A is used. Because NADH is required for dimerization of AIFM1, this implies that, as for the MIA40-AIFM1 complex, only the AIFM1 dimer can bind to AK2A.

(H) Isothermal titration calorimetry (ITC) analysis of AIFM1 together with AK2A or AK2B in the presence of NADH. AIFM1 and AK2A bind to each other with a K_d of approximately 0.44 μM and stoichiometry between AIFM1 and AK2A of approximately 2 to 1. AK2B does not interact with AIFM1 in this experiment.

(I) The interaction sites in MIA40 and AK2A for their interaction with AIFM1. The interaction site in MIA40 spans roughly the first 15 aa and contains conserved charged and aromatic aa. AK2A interacts with AIFM1 via the last 9 aa. Likewise, in this aa patch, conserved charged and aromatic/aliphatic aa are present.

Table 1. Cryo-EM data collection and processing

	AIFM1 dimer	AIFM-AK2	AIFM1-MIA40
PDB	9GQY	9GR0	9GQZ
EMDB	51514	51516	51515
Data collection and processing			
Microscope	Titan Krios G4i	Titan Krios G4i	Titan Krios G3i
Voltage (keV)	300	300	300
Magnification	120,000	120,000	120,000
Pixel size at detector (Å/pixel)	0.46	0.58	0.654
Total electron exposure (e^{-}/Å^2)	50	50	50.82/50.57
Number of frames	468	468	48
Defocus range (μm)	0.7–1.7	0.7–1.7	0.6–2.6
Automation software	EPU	EPU	EPU
Energy filter	Selectris	Selectris	N/A
Micrographs collected (no.)	15,440	4,542	1,907/2,084
For each reconstruction			
Final particles (no.)	227,866	307,496	291,656
Space group	P1	P1	P1
Map sharpening B factor (Å^2)	124.9	97.7	88.2
Resolution (global, Å)			
FSC 0.5 (unmasked/masked)	3.1/2.91	2.87/2.72	2.98/2.68
FSC 0.143 (unmasked/masked)	2.79/2.75	2.6/2.56	2.37/2.34
FSC 0 (unmasked/masked)	2.76/2.72	2.57/2.54	2.34/2.30
Model composition			
Chains	5	7	7
Atoms	7,068 (hydrogens: 0)	7,224 (hydrogens: 0)	7,336 (hydrogens: 0)
Residues	protein: 887 nucleotide: 0	protein: 905 nucleotide: 0	protein: 915 nucleotide: 0
Water	12	24	17
Ligands	FAD: 2; NAD: 2	FAD: 2; NAD: 2	FAD: 2; NAD: 2
Model refinement			
Bonds (RMSD)			
Length (Å) (# > 4σ)	0.005 (0)	0.003 (0)	0.002 (0)
Angles (°) (# > 4σ)	0.591 (0)	0.527 (0)	0.499 (0)
MolProbity score	1.50	1.48	1.61
Ramachandran plot and validation			
Clash score	4.39	4.30	6.35
Ramachandran plot (%)			
Outliers	0.00	0.00	0.00
Allowed	4.10	3.92	3.88
Favored	95.90	96.08	96.12
Rama-Z (Ramachandran plot Z-score RMSD)			
Whole (N = 893)	−0.40 (0.28)	−0.17 (0.28)	0.40 (0.28)
Helix (N = 247)	1.35 (0.35)	1.16 (0.35)	1.78 (0.34)
Sheet (N = 214)	−0.33 (0.36)	0.63 (0.35)	0.09 (0.34)
Loop (N = 432)	−1.00 (0.29)	−1.30 (0.27)	−0.42 (0.30)
Rotamer outliers (%)	0.28	0.00	0.00
Cβ outliers (%)	NA	NA	NA
Peptide plane (%)			
Cis proline/general	0.0/0.0	0.0/0.0	0.0/0.0

(*Continued on next page*)

Table 1. *Continued*

	AIFM1 dimer	AIFM-AK2	AIFM1-MIA40
Twisted proline/general	0.0/0.0	0.0/0.0	0.0/0.0
CaBLAM outliers (%)	2.18	1.70	2.02
ADP (B-factors)			
Iso/Aniso (#)	7,068/0	7,224/0	7,336/0
Protein (min/max/mean)	9.09/132.73/63.11	6.14/105.04/36.33	22.79/135.12/64.32
Nucleotide (min/max/mean)	—	—	—
Ligand (min/max/mean)	18.37/80.80/37.07	8.74/40.20/21.01	31.78/68.66/46.21
Water (min/max/mean)	18.05/53.20/30.17	12.12/31.41/19.63	30.22/58.08/46.41
Occupancy			
Mean	1.00	1.00	1.00
occ = 1 (%)	100.00	100.00	100.00
0 < occ < 1 (%)	0.00	0.00	0.00
occ > 1 (%)	0.00	0.00	0.00

models of AIFM1 aa 128–611 (lacking aa 511–557 [AIFM1 dimer or AIFM1-MIA40] or aa 511–549 [AIFM1-AK2A] due to flexibility), as well as the interacting residues 232–239 of AK2A and 2–20 of MIA40, into these maps. Despite using full-length AK2A and MIA40, only these interacting parts were observed in our cryo-EM reconstructions. High-resolution cryo-EM reconstructions of both dimeric and complexed AIFM1 enabled a detailed analysis of the structural implications of MIA40 and AK2A binding in solution.

AK2A and MIA40 bind the same β-sheet of the AIFM1 C-terminal domain (aa 480–510 and 559–580) via parallel β-strand complementation, adding one and two β-strands, respectively (Figures 3A and 3B). In the crystal structures of the murine and human AIFM1 dimers, aa 538–544 or 510–516, respectively, occupied the same site.[25,27,32] Unambiguous densities corresponding to AK2A F238 or MIA40 F14 show the added β-strands originating from these proteins rather than AIFM1 (Figures S10A and S10B). AK2A and MIA40 were unambiguously built bound to both AIFM1 protomers, indicating that two AK2A or MIA40 molecules can bind to one AIFM1 dimer in solution. No reconstructions with only one binding site occupied by either AK2A or MIA40 were obtained. MIA40 and AK2A may stabilize the complex, resulting in an over-representation of two MIA40 or AK2A molecules per AIFM1 dimer in the best-resolved reconstructions.

Two sets of hydrophobic interactions stabilize AK2A and MIA40 binding. On one side of the β-sheet, L235 and M237 of AK2A or I13 of MIA40 interact with V505 and V507 of AIFM1. On the other side, a hydrophobic patch (AIFM1 Y347, F508, and Y560) harbors V236 and F238 of AK2A or I12 and F14 of MIA40 (Figure 3C). MIA40 Y3 stacks with F14 to stabilize the second β-strand. In addition, conserved Lys and Asp residues of the complementing β-strand, i.e., AK2A K233/D234 and MIA40 K9/D10, form a possible hydrogen-bonding network with S500, the backbone carbonyl of L502, and T504 side chain of AIFM1 (Figure S10C). Although MIA40 adds two strands to the AIFM1 β-sheet, AK2A adds only one but additionally binds part of the C-loop (aa 550–558) that traverses the AK2A β-strand, possibly forming hydrogen bonds between the backbone carbonyl of AIFM1 Q525 and the backbone amine of AK2A V236 (Figure S10C). A similar C-loop conformation was reported in a structure of a human AIFM1 dimer.[29] Thus, although the interaction site is identical for AK2A and MIA40, other aspects of the interaction are divergent. This is also reflected by the differences in binding affinities of MIA40 and AK2A toward AIFM1 (180[15,48] vs. 437 nM [Figure 2H], respectively) and agrees with *in vitro* and cellular competition experiments showing replacement of AK2A from a preformed AIFM1-AK2A complex by MIA40 but not vice versa (Figures 3D and 3E). Thus, when AIFM1 availability is limited, MIA40 may outcompete AK2A for binding to AIFM1.

Structural changes in AIFM1 upon AK2A or MIA40 binding alter AIFM1 dimer stability and NADH oxidase activity

We then assessed the structural variability between AIFM1 protomers within all complexes aligned to the N-terminal domain near the dimer interface. The AIFM1 dimer and, in particular, AIFM1-MIA40 show inter-protomer variability predominantly of NAD binding and parts of the C-terminal domain, whereas little displacement was seen when comparing AK2A-bound protomers (Figure S11). Comparing the conformations of AIFM1 protomers between complexes, AIFM1-AK2A protomers closely resembled AIFM1 dimer protomers (Figures S12A and S12B). MIA40, however, induced up to 3 Å Cα RMSD (root-mean-square deviation) variability compared with the AIFM1 dimer, particularly of the NAD-binding domain, with the strongest displacement of the α-helix contacting the C-terminal domain (aa 345–359) (Figure S12C). The NAD-binding domain shifts toward the N-terminal domain and dimer interface, also reflected in the relative position of the NAD and FAD cofactors upon MIA40 binding. However, in AIFM1-MIA40, the distance between the nicotinamide and isoalloxazine rings is reduced by approx. 0.2 Å. The position of the adenines differs by up to >2 Å when comparing the AIFM1 dimer and the AIFM1-MIA40 complex (Figure 4A). Thus, the tight interaction of MIA40 leads to a compaction of AIFM1 that affects the active site via displacement of the NAD-binding domain.

CellPress
OPEN ACCESS

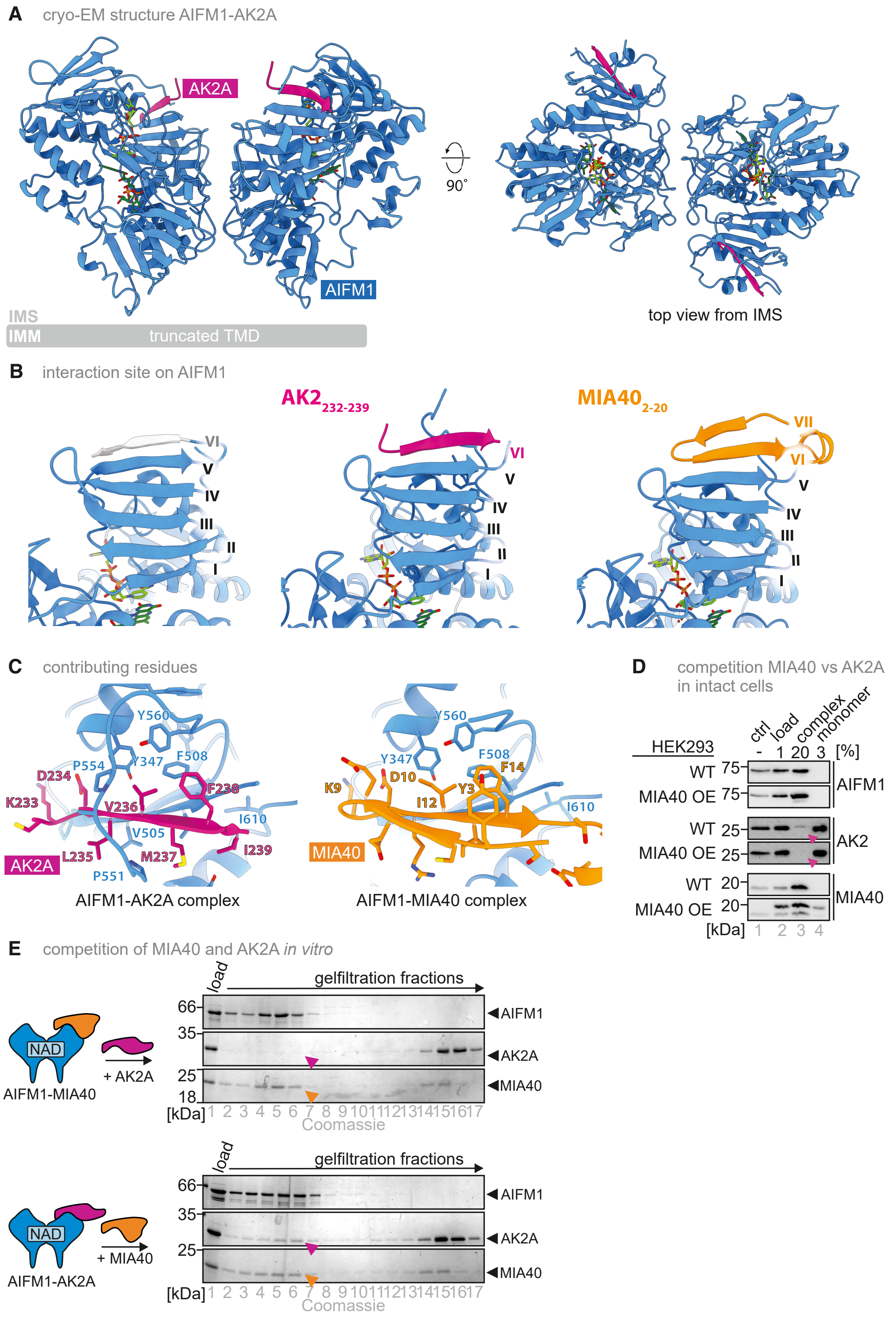

(legend on next page)

AIFM1 F310, E314, and K177 are key residues binding NAD and FAD, in agreement with published structures of AIFM1 dimers.[27,32] MIA40 binding propagates conformational changes to the active site. Specifically, the α-helix containing E314 and F310 (aa 310–326) moves closer to the cofactors by 0.2–0.6 Å (Figures 4B and S13). The Cα distances between F310, E314, and H454 to NAD, and between K177, E314, and the FAD isoalloxazine, are reduced in AIFM1-MIA40 as compared with the AIFM1 dimer or AIFM1-AK2A (Figure 4B). E314 forms hydrogen bonds with NAD and a salt bridge with K177, which then bonds with the N5 atom of the FAD isoalloxazine ring (Figure S13D). Despite uncertainties in the side chain densities of K177 (probably flexible) and E314 (degraded by beam-induced damage), our AIFM1-MIA40 map suggests distinct conformations of E314 and K177 in the AIFM1 protomers of AIFM1-MIA40: one resembles the AIFM1 dimer and AIFM1-AK2A structures, whereas the other K177 side chain points away from the cofactors, releasing interactions with E314 and the FAD isoalloxazine. The unclear side chain density in this region indicates conformational variability, reducing local resolution. In one MIA40-bound protomer, F310 and H454 show different conformations as compared with the other AIFM1 protomers solved, which may affect cofactor engagement.

Taken together, AK2A and MIA40 bind the same site but with distinct binding modes and structural consequences. AK2A adds one β-strand and binds part of the AIFM1 C-loop. AK2A binding rigidifies the AIFM1 dimer, indicated by low inter-protomer variability. MIA40 adds two β-strands and leads to a compaction of AIFM1, in particular the NAD-binding domain, leading to a compacted active site and shorter distance between the nicotinamide of NAD and the isoalloxazine of FAD. Overall, MIA40 binding compacts the hydrogen-bonding network around the cofactors, reducing the distance between FAD and NAD, which may enhance charge transfer.

All our structures resemble reduced AIFM1, i.e., NAD^+ bound after CTC formation. Consequently, we observe the hallmark re-orientation conformation of the "aromatic tunnel" side chains (F310, Y347, W351, F482, Y492, F508, Y560, and W579) connecting the central cofactors and periphery of the C-terminal domain[26] (Figures 4C and S14). Concurrently, a loop connecting two C-terminal β-strands (aa 487–489) releases W198 of the so-called β-clasp that is part of the regulatory β-hairpin (aa 190–202) (Figures 4C and S14B). Consequently, the C-loop is released, providing surface accessibility to the binding site of electron acceptors or a second NADH cofactor.[29] We show that AK2A and MIA40 extend the aromatic tunnel by one (AK2A F238) or two (MIA40 F14, Y3) aromatic residues (Figures 3C, 4C, and S14B). Thereby, AK2A and MIA40 binding represent a conformational lock of the NAD-bound conformation of the "aromatic tunnel" and the released C-loop. Specifically, AK2A F238 and MIA40 F14 prevent re-arrangement of Y560 and, as a consequence, W579 and Y492, to arrest the loop aa 487–489 in a conformation that does not allow the stacking of P488 and W196 that contributes to β-clasp and C-loop stabilization (Figures 3C, 4C, and S14B).

We next assessed stability and redox properties of AIFM1-AK2A and AIFM1-MIA40 complexes (Figure 4D). To this end, we employed purified MIA40 or AK2A or derived AIFM1-interacting peptides (Figure 4E). First, AK2A or MIA40 had a strong impact on AIFM1 complex stability, in line with our structural observations (Figure 4F). Although NADH leads to rapid AIFM1 dimerization,[15,48] dimers dissociated with a half-life ($t_{1/2}$) of ~4 h upon removal of excess NADH. Binding of purified AK2A or MIA40 proteins or interacting peptides strongly stabilized the complex ($t_{1/2} > 7$ h; Figure 4F), along with inhibited re-oxidation of $FADH_2$ (Figure 4G). MIA40 or AK2A peptides stabilized reduced FAD, albeit less than full-length proteins (Figure 4G). AIFM1 redox properties were assessed in a 2,6-dichlorophenolindophenol (DCIP)-reduction assay[26] (Figure 4H). Using AIFM1 alone, we observed efficient NADH oxidation with an apparent K_M and k_{cat} toward NADH of 0.37 mM (H_2O, solvent for MIA40 peptides)/0.23 mM (dimethyl sulfoxide, DMSO, solvent for AK2 peptides) and 0.50 s^{-1} (H_2O) and 0.37 s^{-1} (DMSO), respectively (Figure 4I). The MIA40 peptide decreased the apparent K_M and increased k_{cat} (0.09 mM, 0.89 s^{-1}), indicating increased AIFM1 redox activity even at physiological concentrations of NADH (5–160 μM, total cytosolic NAD between 50 and 500 μM).[49–53] A MIA40 peptide with the aromatic Y3 and F14 replaced by isoleucine failed to exert these effects, emphasizing the importance of the stabilization of the aromatic tunnel conformation by MIA40. AK2A peptides did not change the K_M but increased k_{cat} (0.28 mM, 0.71 s^{-1}), implying that AIFM1 activity was increased by AK2A, albeit to a lesser extent than by MIA40. Again, an F238L AK2A peptide did not increase k_{cat}. AIFM1 did not show activity against NADPH in the DCIP-reduction assay, and neither AK2A nor MIA40 peptides increased this negligible activity, suggesting that NADH is the natural reductant employed by AIFM1 (Figure S15B).[26,29]

Collectively, we show that MIA40 and AK2A binding stabilized the AIFM1 complex and strongly increased

Figure 3. Atomic models of the AIFM1 dimer, AIFM1-AK2A, and the AIFM1-MIA40 complexes reveal binding interfaces

(A) Atomic model of the AIFM1 dimer (blue) bound to AK2A (purple). Of AK2A, high-resolution density accounting for aa 232–239 was obtained at both C-terminal domains of the AIFM1 protomers. The remainder of AK2A was presumably flexible and therefore not reconstructed by single-particle cryo-EM at high resolution.

(B) AK2A (aa 232–239) (purple) and MIA40 (aa 2–20) (orange) bind to the AIFM1 C-terminal domain via parallel β-strand complementation. Gray: additional β-strand of AIFM1 (aa 511–516) in the AIFM1 dimer in the absence of AK2A or MIA40. No densities that could correspond to other parts of AK2A and MIA40 could be detected by cryo-EM nor by negative-stain EM (data not shown), suggesting that the rest of these interaction partners do not stably interact with AIFM1 under the conditions used but remain largely flexible relative to AIMF1. Amino acids 580–612 of AIFM1 are omitted for clarity.

(C) Detailed view of residues stabilizing the interaction of AK2A and MIA40 with AIFM1.

(D) Overexpression of MIA40 in HEK293 cells replaces AK2A from the AIFM1-AK2A complex. Experiment was performed as described in Figure 2F with WT HEK293 cells and cells overexpressing MIA40 (MIA40 OE). Purple arrowheads indicate that in MIA40 OE cells, AK2A is lost from the complex fraction.

(E) *In vitro* competition assay between AK2A and MIA40 for binding to AIFM1. Experiment was performed as described in Figure 2G, except that after pre-binding of MIA40 to AIFM1, AK2A was added (upper panel) or vice versa (lower panel). Although AK2A is not able to bind to AIFM1 if MIA40 is already present, MIA40 can bind to AIFM1 even if AK2A was pre-bound. Purple and orange arrowheads indicate the position of AK2A and MIA40, respectively, in the complex fraction.

CellPress
OPEN ACCESS

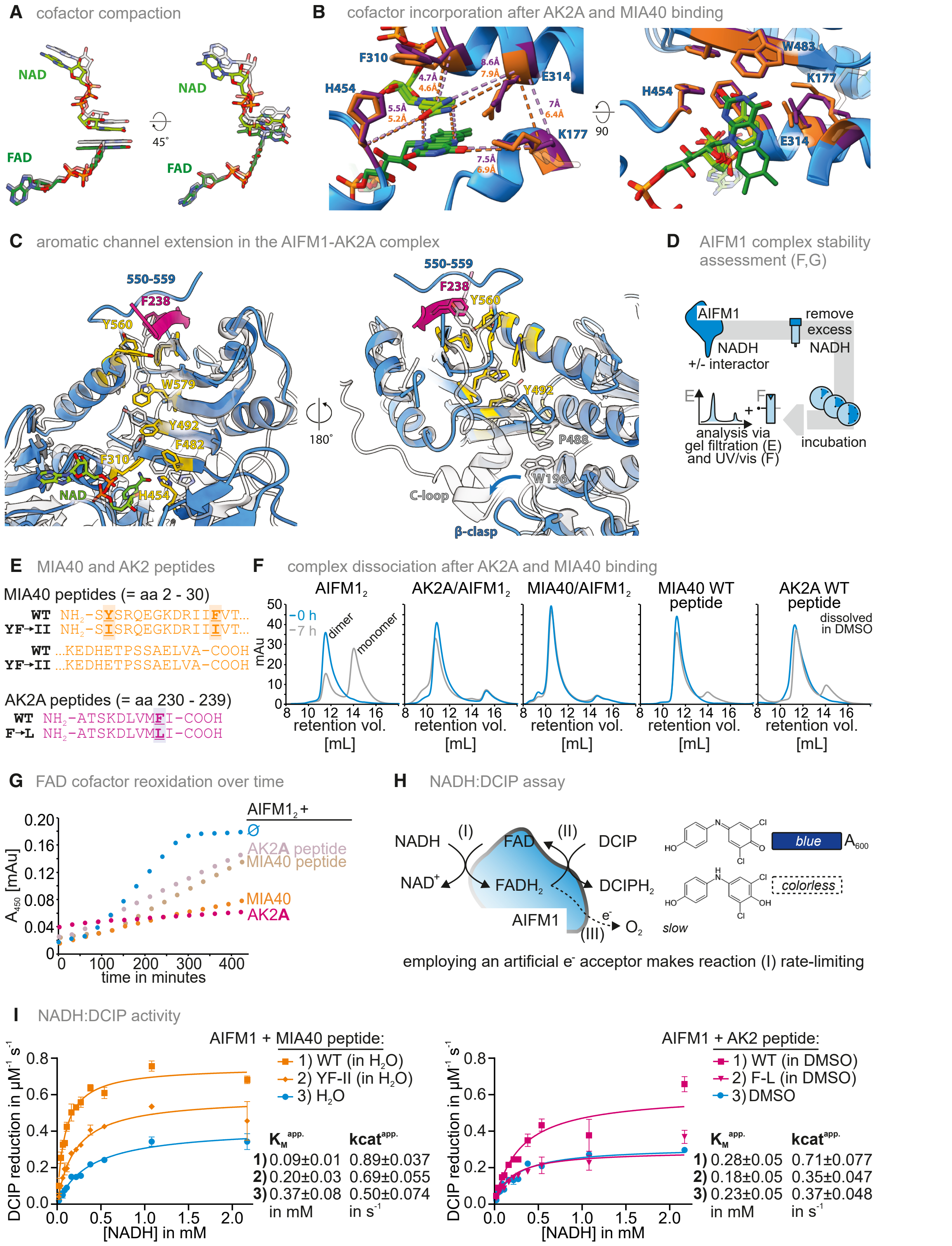

(legend on next page)

NADH-oxidation activity. Our structural analysis provides a mechanistic explanation of these observations. Extension of the "aromatic tunnel" stabilizes the dimer-competent and C-loop-released conformation impacting both complex stability and redox activity. Stabilization is also supported by reduced inter-protomer variability (Figure S11) and a higher resolution of AIFM1-AK2A, with less data than for the AIFM1 dimer under identical sample preparation and imaging conditions (Figures S6 and S7). AK2A further restricts C-loop mobility by tethering aa 550–558. MIA40 impacts the CTC and cofactor-binding site, which could have an impact on the redox activity of AIFM1.

AK2A is responsive to AIFM1 levels and complements the AK2 KO during growth on a respiratory carbon source

We sought to explore the biological importance of the AIFM1-AK2A interaction. In HEK293 and other cells, the AIFM1-interacting AK2A isoform is less abundant than AK2B (Figures 2E and 5A) and its levels seem to depend on AIFM1 (Figures 5B and 5C). We previously presented a MIA40-dependent mechanism of AK2 import.[54] Overall levels of AK2 were affected by loss of MIA40[54] but only very mildly by AIFM1 loss.[15] Based on these data, it seems that AK2A and AK2B are imported by the same mechanism, although AK2A is less stable when not interacting with AIFM1.

To address the physiological impact of AIFM1-AK2A binding, we performed proliferation assays using AK2 KO cells complemented with AK2A or AK2B. AK2 KO cells showed slightly impaired growth on glucose and upon transfer to galactose (galactose shift, Figure 5D). Complementation with AK2A and AK2B improved growth on glucose, albeit AK2B-complemented cells showed slightly reduced growth compared with AK2A at lower cell density. This effect was more pronounced upon galactose shift, where AK2A but not AK2B fully complemented the AK2 KO.

We hypothesize that AIFM1 positions AK2A to provide proximity to mitochondrial inner membrane (IMM) translocases. To test this, we compared the AIFM1-HA and SMAC^{MTS}-AIFM1$^{(103–613)}$-HA interactomes (Figure 5E). The latter AIFM1 variant is soluble in the IMS, whereas AIFM1-HA remains IMM bound. Interactome comparison revealed MICOS components, SLC25 carriers, and ATP synthase subunits enriched for AIFM1-HA, whereas both variants interacted with MIA40 and AK2A (Figure 5F).

In summary, AK2A is sufficient to fully complement AK2 KO cells. Together with our structural and interactomic analyses, we provide evidence that AIFM1 brings AK2 into proximity of ADP/ATP carriers to facilitate the transport of adenine nucleotides across the IMM, which might be particularly important during shifts of carbon sources and the corresponding metabolic changes.

DISCUSSION

Role of AIFM1 in IMS organization

AIFM1, initially linked to cell death, is now recognized for its pro-survival role in complex I biogenesis.[12] Several studies have revealed its involvement in the mitochondrial disulfide relay by facilitating MIA40 import and activation.[13–15,22,23] We discovered AIFM1 interactors, including AK2A and MICOS complex subunits. Focusing on AK2A, we found that AK2A, but not the more abundant AK2B, is stabilized by AIFM1 binding via its last seven aa that are absent in AK2B. AK2A and MIA40 bind the same site in the AIFM1 C-terminal domain by β-strand complementation. In competition assays MIA40 displaced AK2A from AIFM1, suggesting that limited AIFM1 availability may destabilize AK2A, potentially altering mitochondrial metabolism.

Notably, a pathogenic AK2 variant lacking the last aa at the C terminus (K233*) was identified in a patient with hematopoiesis defects. As a consequence, this patient only expressed AK2B. Because AK2B could apparently not compensate for the loss

Figure 4. Structural impact of MIA40 and AK2A binding on functional domains and conformations of AIFM1

(A) Isolated view of the NAD and FAD cofactor orientations in MIA40-AIFM1 (light and dark green, respectively) compared with the dimer (transparent, gray overlay).

(B) Detailed view of the AIFM1 active site. Overlay of AK2A- and MIA40-bound AIFM1 residues stabilizing the cofactors, shown in stick representation for AK2A (purple) and MIA40 (orange). Dashed lines: distances between the Cα atoms of the respective residues or cofactor atoms, colored according to AK2A-bound (purple) and MIA40-bound (orange) dimer. The second observed conformation of AIFM1 K177 is visualized as transparent.

(C) Structural details of aromatic aa side chains forming the "aromatic tunnel" and the conformational impact of AK2A binding (orange). Aromatic tunnel residues and the NAD binding H454 are highlighted in yellow. The AIFM1 model in the monomeric, oxidized conformation (PDB: 4BV6; Ferreira et al.[29]) is shown as a gray, transparent overlay.

(D) Peptides representing the AIFM1 interaction sites in AK2A and MIA40 and their respective aromatic mutant variants.

(E) Strategy for the assessment of different AIFM1 complexes *in vitro*. The AIFM1 dimer, AIFM1-MIA40, and AIFM1-AK2A complexes were established by incubating AIFM1 with AK2A, MIA40, or the respective interaction site peptides in the presence of NADH. Unbound NADH was rapidly removed using gel filtration. Samples were taken at different times and analyzed by gel filtration to visualize the respective shares of complexes and AIFM1 monomer (F) or ultraviolet-visible (UV-vis) spectroscopy at 450 nm to visualize the redox state of the AIFM1 redox cofactor, FAD (G).

(F) Stability of the AIFM1 dimer and the AIFM1-AK2A and AIFM1-MIA40 complexes. 0 and 7 h after removal of excess NADH, samples were analyzed by gel filtration and absorbance at 280 nm was used as an indicator for proteins in the respective fractions. The AIFM1 dimer rapidly disassembled, whereas complexes of AIFM1 with AK2A, MIA40, or the respective binding site peptides were strongly stabilized.

(G) Redox state of the FAD cofactor. The redox state of FAD was continuously monitored over time after the removal of excess NADH. An increase in the absorbance signal indicates the oxidation of $FADH_2$ over time. In the AIFM1 dimer, $FADH_2$ became rapidly oxidized, whereas complexes of AIFM1 with AK2A and MIA40 or (and to a lesser extent) the respective binding site peptides of AK2A or MIA40 maintained $FADH_2$ for longer times in the reduced state.

(H) Strategy for the assessment of AIFM1 NADH oxidoreductase activity using 2,6-dichlorophenolindophenol (DCIP) as an artificial electron acceptor.

(I) Changes in enzymatic activity of AIFM1 upon binding of MIA40- or AK2A-binding-site peptides. Binding of the MIA40-binding-site peptide increases the apparent k_{cat} and lowers the K_M toward NADH. For the AK2A-binding peptide, the apparent k_{cat} also increases whereas the K_M toward NADH remains similar. All changes are attenuated if, instead of the WT peptides, peptides are used, in which aromatic residues are mutated to aliphatic residues (MIA40-Y3I, F14I [YF – II] and AK2-F238L [F – L]).

CellPress
OPEN ACCESS

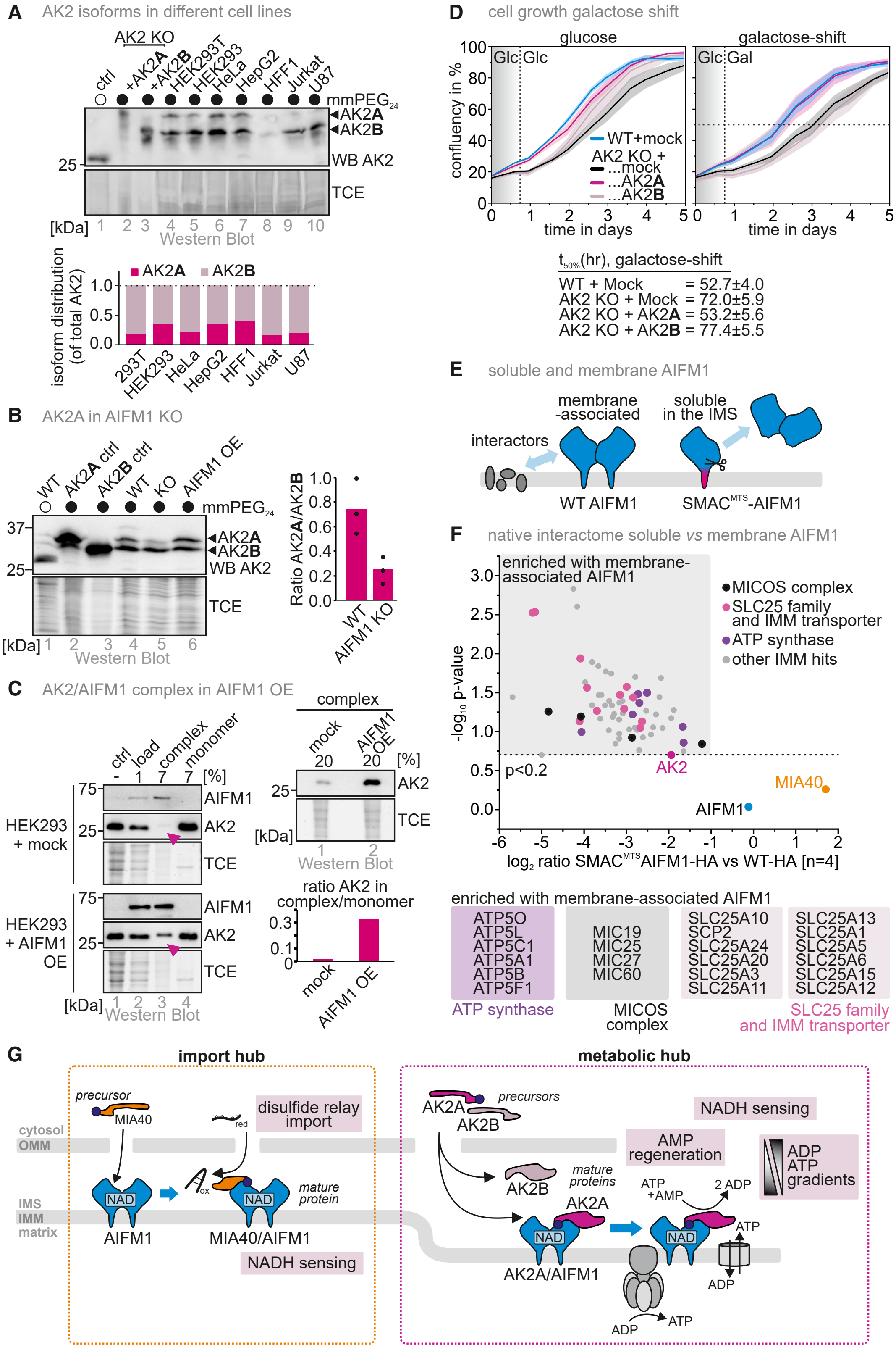

(legend on next page)

of AK2A, this clinical evidence further underscores the pathophysiological relevance of AIFM1-AK2A binding.[46]

AIFM1 complex stability and stoichiometry

Our structural data explain how AK2A and MIA40 binding stabilizes the AIFM1 dimer. We propose that by capping the aromatic tunnel at the AIFM1 C-terminal domain, MIA40 and AK2A stabilize this conformational hallmark of reduced, dimeric AIFM1. AK2A further interacts with aa 550–558, disfavoring C-loop engagement with its inhibitory binding site at the AIFM1 C-terminal domain. Thereby, MIA40 and AK2A binding favor C-loop detachment and electron acceptor binding site accessibility.

Our ITC measurements suggest a stoichiometry of one MIA40/AK2A per AIFM1 dimer, whereas gel filtration after long-term co-incubation indicated the presence of two MIA40/AK2A protomers per AIFM1 dimer. Accordingly, our and previously published structures[32] show two AK2A or MIA40 molecules per AIFM1 dimer. The inter-protomer variability of AIFM1 within the AIFM1 dimer and AIFM1-MIA40 suggests structural asymmetry within the complex. This is also reflected in the variability of local cryo-EM densities of the sixth β-strand of AIFM1 added to the C-terminal β-sheet in the dimer, as well as in reported inter-protomer differences within the dimer.[26,32] The inherent structural variability within the AIFM1 dimer may be linked to the variability within the AIFM1-MIA40 complex that we observe in our cryo-EM structure, which could be induced by MIA40 initially binding to one of the AIFM1 protomers. One or two MIA40 or AK2A molecules per AIFM1 dimer also leaves open the possibility of a mixed complex comprising AIFM1 and both MIA40 and AK2A.

Impact on NADH turnover

AIFM1 can act as NADH oxidase or NADH:ubiquinone oxidoreductase, although the reaction is not very efficient. Accordingly, its K_M toward NADH was relatively high in the DCIP assay (K_M = 370 μM), being well above physiological levels (5–160 μM NADH[49–53]). MIA40 or AK2A binding, however, significantly increased NADH oxidation activity in the physiological NADH range. The increased oxidoreductase activity of the complex can be explained by the impact of AK2A and MIA40 on dimer stability and C-loop detachment, thus favoring surface accessibility of the electron acceptor binding site. AK2A additionally binds part of the C-loop, further preventing its (re-) attachment. MIA40 may impact AIFM1 oxidoreductase activity by other means, namely by propagating compaction to the CTC, reducing the FAD-NAD distance. Therefore, MIA40 and AK2A have a dual impact on AIFM1 function, affecting dimer stability and oxidoreductase activity by directly impacting the concerted structural rearrangements that mediate dimerization and C-loop release upon CTC formation.

Positioning AK2A for efficient ATP-ADP exchange

NADH enables AIFM1 dimerization and AK2A binding (Figure 5G), whereas growth assays confirm the importance of the AIFM1-AK2A interaction because AK2B cannot complement the AK2 KO despite similar enzymatic activity.

The AK2 A positioning near key metabolic hubs might serve two purposes: (1) converting AMP to ADP, thereby preventing accumulation of a non-transportable adenine nucleotide, and (2) maintaining the adenine nucleotide gradient, optimizing ADP/ATP exchange. This is further supported by the low K_M of AK2 toward AMP.[54]

Because both AIFM1-AK2A and AIFM1-MIA40 remain stable complexes in the absence of NADH, we assume that short-term fluctuations leave both complexes intact. Interestingly, AK2 is stringently inhibited by elevated AMP levels, which are possibly present under starvation conditions.[54,55] Thus, during short-term starvation and upon increasing AMP levels, AK2A activity in the AIFM1-AK2A complex might be switched off, decreasing the efficiency of ADP-ATP exchange across the IMM and the IMS. Prolonged, low levels of NADH might then also affect the initial formation of the AIFM1-AK2A complex.

In summary, AIFM1 regulates the two branches of the OXPHOS system because it controls complex I biogenesis and ADP availability, which is key to maintaining the activity of the ATP synthase.

Limitations of the study

We identified AK2A as an interaction partner of AIFM1. Our structural and biochemical analyses of the complex strongly support a model in which AK2A is positioned by AIFM1 close to ADP/ATP carriers and the ATP-synthase in the IMM, thereby supporting metabolic adaptation processes. However, several

Figure 5. A cellular function of the AIFM1-AK2A complex in facilitating ADP/ATP exchange across the mitochondrial inner membrane (IMM)

(A) Ratio between AK2 isoforms. As in Figure 2E, except that the thiol shift assay was performed on lysates of different cells. In all analyzed cell lines, AK2A constitutes the minor isoform with shares of 20%–40%.

(B) Levels of AK2 isoforms upon AIFM1 depletion. Experiment was performed as in (A) with the indicated cell lines. Depletion of AIFM1 results in decreased AK2A levels while leaving AK2B mostly unchanged. N = 3 biological replicates.

(C) Levels of AIFM1-AK2A complex upon AIFM1 overexpression. Experiment was performed as in Figure 2F. Overexpression of AIFM1 results in increased amounts of AIFM1-AK2A complex.

(D) Assessment of proliferation of AK2 KO HEK293 cells complemented with either AK2A or AK2B. AK2 KO grows slower compared with WT cells on glucose as well as upon galactose shift. Complementation of the AK2 KO with AK2A rescues growth of AK2 KO cells on glucose and upon galactose shift, whereas AK2B rescues only glucose-grown cells.

(E) Strategy for the assessment of AIFM1-transmembrane-domain-dependent interaction partners. AIFM1 KO cell lines inducibly and stably expressing either WT AIFM1-HA or AIFM1-HA with a well-established cleavable SMAC mitochondrial targeting signal (SMAC-MTS). This latter MTS targets AIFM1 also to the IMS where, after import and processing, it is present in a soluble non-membrane-bound state. Interactomes of these proteins will be compared.

(F) Interactome of WT AIFM1-HA and SMAC-MTS AIFM-HA. Many SLC25 family members and MICOS subunits as well as ATP synthase subunits were enriched on WT AIFM1.

(G) Model: the AIFM1-AK2 interaction facilitates the positioning of AK2 close to ADP/ATP transporters in the IMM. This promotes growth under conditions requiring mitochondrial respiration.

open questions remain to be addressed in future studies, including the physiological role of the AIFM1-AK2A complex in complex model organisms, the regulation of AIFM1-AK2A complex dynamics in dependence of cellular NADH levels, and the crosstalk between AK2 function and AIFM1 redox function.

RESOURCE AVAILABILITY

Lead contact

Requests for further information and resources should be directed to, and will be fulfilled by, the lead contact, Jan Riemer (jan.riemer@uni-koeln.de).

Materials availability

The datasets generated and/or analyzed during the current study are available from the corresponding authors on reasonable request. lead contact

Data and code availability

- The mass spectrometry proteomics data have been deposited to the ProteomeXchange Consortium via the PRIDE[56] partner repository with the dataset identifier PRIDE: PXD055617. The protein-protein interaction microarray data can be found in Data S1. Atomic coordinates and EM maps have been deposited at the Protein Data Bank (PDB) with the accession numbers PDB: 9GQY and EMD-51514 (AIFM1 dimer), PDB: 9GQZ and EMD-51515 (AIFM1-MIA40), and PDB: 9GR0 and EMD-51516 (AIFM1-AK2A).
- This paper does not report original code.
- Any additional information required to reanalyze the data reported in this paper is available from the lead contact upon request.

ACKNOWLEDGMENTS

The Deutsche Forschungsgemeinschaft (DFG) funds the research of J.R. through the grants RI2150/5-1 (project ID 435235019), SPP2453 (project ID 541742459), RTG2550/1 (project ID 411422114), CRC1218 (project ID 269925409), and CRC1678 (project ID 520471345). S.P. is funded by CMMC core funding (JRG XI), by the DFG - SFB1430 (project ID 424228829), and the CANTAR network funded by the state of Northrhine-Westphalia. D.B. is supported by DZNE core budget and the SPP2453 (project ID 541742459). D.B. is a member of the ETERNITY project consortium, funded by the European Union through Horizon Europe Marie Skłodowska-Curie Actions Doctoral Networks (MSCA-DN) under the grant ID 101072759. We thank the CECAD Proteomics Facility for the analysis of proteome data; the facility is supported by the DFG-large instrument grant INST 216/1163-1 FUGG. We thank the CECAD Imaging and the StruBiTEM cryo-EM (Cologne, funded by DFG grant INST 216/949-1 FUGG) facilities, in particular Monika Gunkel and Elmar Behrmann. Cryo-EM data of the AIFM1 dimer and AIFM1-AK2A were collected at the cryo-EM center of the University of Münster (cryo-EM SoN, project ID 496113311). We thank Christos Gatsogiannis and Alexander Neuhaus for support. We thank Ms Christiane Bartling-Kirsch and Dr. Lena Wischhof for their help. We thank Kathrin Ulrich, Matthias Weith, and members of the Riemer lab for critical reading of the manuscript. We thank Anja Wittmann and Anika Seiler for technical support.

AUTHOR CONTRIBUTIONS

J. Riemer designed the study and acquired funding. R.A.R. designed and cloned constructs. R.A.R., K.L., and S.L.S. designed and generated cell lines. R.A.R., D.S., S.M., P.G., K.W., and S.G. carried out the biochemical experiments. J. Racho and R.A.R. performed immunoprecipitations and label-free proteomics experiments. C.P. and J.D. performed SILAC proteomics experiments. S.P., E.P., and T.H.D.N. solved and verified the cryo-EM structures. S.G. performed cell proliferation assays. D.E., M.M., and D.B. performed *in vitro* protein-protein profiling experiments and validation studies in cells. K.S. and I.N. designed and synthesized peptides. All authors analyzed data. J. Riemer, S.P., D.B., and R.A.R. wrote the first manuscript draft. All authors provided input to the manuscript and proofread it.

DECLARATION OF INTERESTS

The authors have nothing to disclose and no conflict of interest.

STAR★METHODS

Detailed methods are provided in the online version of this paper and include the following:

- KEY RESOURCES TABLE
- EXPERIMENTAL MODEL AND STUDY PARTICIPANT DETAILS
- METHOD DETAILS
  - Plasmids, cell lines, chemical treatments
  - Generation of HEK293 knockout cells
  - Immunoprecipitation
  - SILAC-based mass spectrometry
  - Interaction profiling by using microchips
  - Immunoblotting and image acquisition
  - In vitro AK2 activity assay
  - Analytical size-exclusion chromatography
  - Thiol shift assay to differentiate AK2 isoforms
  - Peptide synthesis
  - Protein purification
  - In vitro complex reconstitution
  - Isothermal titration calorimetry (ITC)
  - Cryo-EM grid preparation and data collection
  - Cryo-EM data processing
  - Model building and refinement
  - DCIP activity assay
  - Cell proliferation assay
- QUANTIFICATION AND STATISTICAL ANALYSIS

SUPPLEMENTAL INFORMATION

Supplemental information can be found online at https://doi.org/10.1016/j.molcel.2025.05.036.

Received: September 13, 2024
Revised: March 28, 2025
Accepted: May 30, 2025
Published: June 26, 2025

REFERENCES

1. Herrmann, J.M., and Riemer, J. (2021). Apoptosis inducing factor and mitochondrial NADH dehydrogenases: redox-controlled gear boxes to switch between mitochondrial biogenesis and cell death. Biol. Chem. *402*, 289–297. https://doi.org/10.1515/hsz-2020-0254.
2. Novo, N., Ferreira, P., and Medina, M. (2021). The apoptosis-inducing factor family: Moonlighting proteins in the crosstalk between mitochondria and nuclei. IUBMB Life *73*, 568–581. https://doi.org/10.1002/iub.2390.
3. Reinhardt, C., Arena, G., Nedara, K., Edwards, R., Brenner, C., Tokatlidis, K., and Modjtahedi, N. (2020). AIF meets the CHCHD4/Mia40-dependent mitochondrial import pathway. Biochim. Biophys. Acta Mol. Basis Dis. *1866*, 165746. https://doi.org/10.1016/j.bbadis.2020.165746.
4. Wischhof, L., Scifo, E., Ehninger, D., and Bano, D. (2022). AIFM1 beyond cell death: An overview of this OXPHOS-inducing factor in mitochondrial diseases. EBiomedicine *83*, 104231. https://doi.org/10.1016/j.ebiom.2022.104231.
5. Bano, D., and Prehn, J.H.M. (2018). Apoptosis-Inducing Factor (AIF) in Physiology and Disease: The Tale of a Repented Natural Born Killer. EBiomedicine *30*, 29–37. https://doi.org/10.1016/j.ebiom.2018.03.016.

6. Klein, J.A., Longo-Guess, C.M., Rossmann, M.P., Seburn, K.L., Hurd, R. E., Frankel, W.N., Bronson, R.T., and Ackerman, S.L. (2002). The harlequin mouse mutation downregulates apoptosis-inducing factor. Nature *419*, 367–374. https://doi.org/10.1038/nature01034.

7. van Empel, V.P.M., Bertrand, A.T., van der Nagel, R., Kostin, S., Doevendans, P.A., Crijns, H.J., de Wit, E., Sluiter, W., Ackerman, S.L., and De Windt, L.J. (2005). Downregulation of apoptosis-inducing factor in harlequin mutant mice sensitizes the myocardium to oxidative stress-related cell death and pressure overload-induced decompensation. Circ. Res. *96*, e92–e101. https://doi.org/10.1161/01.RES.0000172081.30327.28.

8. van Empel, V.P.M., Bertrand, A.T., van Oort, R.J., van der Nagel, R., Engelen, M., van Rijen, H.V., Doevendans, P.A., Crijns, H.J., Ackerman, S.L., Sluiter, W., et al. (2006). EUK-8, a superoxide dismutase and catalase mimetic, reduces cardiac oxidative stress and ameliorates pressure overload-induced heart failure in the harlequin mouse mutant. J. Am. Coll. Cardiol. *48*, 824–832. https://doi.org/10.1016/j.jacc.2006.02.075.

9. Ghezzi, D., Sevrioukova, I., Invernizzi, F., Lamperti, C., Mora, M., D'Adamo, P., Novara, F., Zuffardi, O., Uziel, G., and Zeviani, M. (2010). Severe X-linked mitochondrial encephalomyopathy associated with a mutation in apoptosis-inducing factor. Am. J. Hum. Genet. *86*, 639–649. https://doi.org/10.1016/j.ajhg.2010.03.002.

10. Bertan, F., Wischhof, L., Scifo, E., Guranda, M., Jackson, J., Marsal-Cots, A., Piazzesi, A., Stork, M., Peitz, M., Prehn, J.H.M., et al. (2021). Comparative analysis of CI- and CIV-containing respiratory supercomplexes at single-cell resolution. Cell Rep. Methods *1*, 100002. https://doi.org/10.1016/j.crmeth.2021.100002.

11. Wischhof, L., Gioran, A., Sonntag-Bensch, D., Piazzesi, A., Stork, M., Nicotera, P., and Bano, D. (2018). A disease-associated Aifm1 variant induces severe myopathy in knockin mice. Mol. Metab. *13*, 10–23. https://doi.org/10.1016/j.molmet.2018.05.002.

12. Vahsen, N., Candé, C., Brière, J.J., Bénit, P., Joza, N., Larochette, N., Mastroberardino, P.G., Pequignot, M.O., Casares, N., Lazar, V., et al. (2004). AIF deficiency compromises oxidative phosphorylation. EMBO J. *23*, 4679–4689. https://doi.org/10.1038/sj.emboj.7600461.

13. Hangen, E., Féraud, O., Lachkar, S., Mou, H., Doti, N., Fimia, G.M., Lam, N. V., Zhu, C., Godin, I., Muller, K., et al. (2015). Interaction between AIF and CHCHD4 Regulates Respiratory Chain Biogenesis. Mol. Cell *58*, 1001–1014. https://doi.org/10.1016/j.molcel.2015.04.020.

14. Meyer, K., Buettner, S., Ghezzi, D., Zeviani, M., Bano, D., and Nicotera, P. (2015). Loss of apoptosis-inducing factor critically affects MIA40 function. Cell Death Dis. *6*, e1814. https://doi.org/10.1038/cddis.2015.170.

15. Salscheider, S.L., Gerlich, S., Cabrera-Orefice, A., Peker, E., Rothemann, R.A., Murschall, L.M., Finger, Y., Szczepanowska, K., Ahmadi, Z.A., Guerrero-Castillo, S., et al. (2022). AIFM1 is a component of the mitochondrial disulfide relay that drives complex I assembly through efficient import of NDUFS5. EMBO J. *41*, e110784. https://doi.org/10.15252/embj.2022110784.

16. Zarges, C., and Riemer, J. (2024). Oxidative protein folding in the intermembrane space of human mitochondria. FEBS Open Bio *14*, 1610–1626. https://doi.org/10.1002/2211-5463.13839.

17. Geldon, S., Fernández-Vizarra, E., and Tokatlidis, K. (2021). Redox-Mediated Regulation of Mitochondrial Biogenesis, Dynamics, and Respiratory Chain Assembly in Yeast and Human Cells. Front. Cell Dev. Biol. *9*, 720656. https://doi.org/10.3389/fcell.2021.720656.

18. Al-Habib, H., and Ashcroft, M. (2021). CHCHD4 (MIA40) and the mitochondrial disulfide relay system. Biochem. Soc. Trans. *49*, 17–27. https://doi.org/10.1042/BST20190232.

19. Backes, S., and Herrmann, J.M. (2017). Protein Translocation into the Intermembrane Space and Matrix of Mitochondria: Mechanisms and Driving Forces. Front. Mol. Biosci. *4*, 83. https://doi.org/10.3389/fmolb.2017.00083.

20. Modjtahedi, N., Tokatlidis, K., Dessen, P., and Kroemer, G. (2016). Mitochondrial Proteins Containing Coiled-Coil-Helix-Coiled-Coil-Helix (CHCH) Domains in Health and Disease. Trends Biochem. Sci. *41*, 245–260. https://doi.org/10.1016/j.tibs.2015.12.004.

21. Stojanovski, D., Bragoszewski, P., and Chacinska, A. (2012). The MIA pathway: a tight bond between protein transport and oxidative folding in mitochondria. Biochim. Biophys. Acta *1823*, 1142–1150. https://doi.org/10.1016/j.bbamcr.2012.04.014.

22. Petrungaro, C., Zimmermann, K.M., Küttner, V., Fischer, M., Dengjel, J., Bogeski, I., and Riemer, J. (2015). The Ca(2+)-Dependent Release of the Mia40-Induced MICU1-MICU2 Dimer from MCU Regulates Mitochondrial Ca(2+) Uptake. Cell Metab. *22*, 721–733. https://doi.org/10.1016/j.cmet.2015.08.019.

23. Murschall, L.M., Gerhards, A., MacVicar, T., Peker, E., Hasberg, L., Wawra, S., Langer, T., and Riemer, J. (2020). The C-terminal region of the oxidoreductase MIA40 stabilizes its cytosolic precursor during mitochondrial import. BMC Biol. *18*, 96. https://doi.org/10.1186/s12915-020-00824-1.

24. Brosey, C.A., Shen, R., and Tainer, J.A. (2025). NADH-bound AIF activates the mitochondrial CHCHD4/MIA40 chaperone by a substrate-mimicry mechanism. EMBO J. *44*, 1220–1248. https://doi.org/10.1038/s44318-024-00360-6.

25. Maté, M.J., Ortiz-Lombardía, M., Boitel, B., Haouz, A., Tello, D., Susin, S. A., Penninger, J., Kroemer, G., and Alzari, P.M. (2002). The crystal structure of the mouse apoptosis-inducing factor AIF. Nat. Struct. Biol. *9*, 442–446. https://doi.org/10.1038/nsb793.

26. Churbanova, I.Y., and Sevrioukova, I.F. (2008). Redox-dependent changes in molecular properties of mitochondrial apoptosis-inducing factor. J. Biol. Chem. *283*, 5622–5631. https://doi.org/10.1074/jbc.M709147200.

27. Sevrioukova, I.F. (2009). Redox-linked conformational dynamics in apoptosis-inducing factor. J. Mol. Biol. *390*, 924–938. https://doi.org/10.1016/j.jmb.2009.05.013.

28. Sorrentino, L., Calogero, A.M., Pandini, V., Vanoni, M.A., Sevrioukova, I.F., and Aliverti, A. (2015). Key Role of the Adenylate Moiety and Integrity of the Adenylate-Binding Site for the NAD(+)/H Binding to Mitochondrial Apoptosis-Inducing Factor. Biochemistry *54*, 6996–7009. https://doi.org/10.1021/acs.biochem.5b00898.

29. Ferreira, P., Villanueva, R., Martínez-Júlvez, M., Herguedas, B., Marcuello, C., Fernandez-Silva, P., Cabon, L., Hermoso, J.A., Lostao, A., Susin, S.A., et al. (2014). Structural insights into the coenzyme mediated monomer-dimer transition of the pro-apoptotic apoptosis inducing factor. Biochemistry *53*, 4204–4215. https://doi.org/10.1021/bi500343r.

30. Brosey, C.A., Ho, C., Long, W.Z., Singh, S., Burnett, K., Hura, G.L., Nix, J. C., Bowman, G.R., Ellenberger, T., and Tainer, J.A. (2016). Defining NADH-Driven Allostery Regulating Apoptosis-Inducing Factor. Structure *24*, 2067–2079. https://doi.org/10.1016/j.str.2016.09.012.

31. Villanueva, R., Ferreira, P., Marcuello, C., Usón, A., Miramar, M.D., Peleato, M.L., Lostao, A., Susin, S.A., and Medina, M. (2015). Key Residues Regulating the Reductase Activity of the Human Mitochondrial Apoptosis Inducing Factor. Biochemistry *54*, 5175–5184. https://doi.org/10.1021/acs.biochem.5b00696.

32. Fagnani, E., Cocomazzi, P., Pellegrino, S., Tedeschi, G., Scalvini, F.G., Cossu, F., Da Vela, S., Aliverti, A., Mastrangelo, E., and Milani, M. (2024). CHCHD4 binding affects the active site of apoptosis inducing factor (AIF): Structural determinants for allosteric regulation. Structure *32*, 594–602.e4. https://doi.org/10.1016/j.str.2024.02.008.

33. Kettwig, M., Schubach, M., Zimmermann, F.A., Klinge, L., Mayr, J.A., Biskup, S., Sperl, W., Gärtner, J., and Huppke, P. (2015). From ventriculomegaly to severe muscular atrophy: expansion of the clinical spectrum related to mutations in AIFM1. Mitochondrion *21*, 12–18. https://doi.org/10.1016/j.mito.2015.01.001.

34. Dzeja, P., and Terzic, A. (2009). Adenylate kinase and AMP signaling networks: metabolic monitoring, signal communication and body energy sensing. Int. J. Mol. Sci. *10*, 1729–1772. https://doi.org/10.3390/ijms10041729.

CellPress
OPEN ACCESS

35. Dzeja, P.P., Zeleznikar, R.J., and Goldberg, N.D. (1998). Adenylate kinase: kinetic behavior in intact cells indicates it is integral to multiple cellular processes. Mol. Cell. Biochem. *184*, 169–182. https://doi.org/10.1023/A:1006859632730.

36. Noma, T. (2005). Dynamics of nucleotide metabolism as a supporter of life phenomena. J. Med. Invest. *52*, 127–136. https://doi.org/10.2152/jmi.52.127.

37. Ruprecht, J.J., and Kunji, E.R.S. (2020). The SLC25 Mitochondrial Carrier Family: Structure and Mechanism. Trends Biochem. Sci. *45*, 244–258. https://doi.org/10.1016/j.tibs.2019.11.001.

38. Dzeja, P.P., Vitkevicius, K.T., Redfield, M.M., Burnett, J.C., and Terzic, A. (1999). Adenylate kinase-catalyzed phosphotransfer in the myocardium: increased contribution in heart failure. Circ. Res. *84*, 1137–1143. https://doi.org/10.1161/01.res.84.10.1137.

39. Pucar, D., Dzeja, P.P., Bast, P., Juranic, N., Macura, S., and Terzic, A. (2001). Cellular energetics in the preconditioned state: protective role for phosphotransfer reactions captured by 18O-assisted 31P NMR. J. Biol. Chem. *276*, 44812–44819. https://doi.org/10.1074/jbc.M104425200.

40. Burkart, A., Shi, X., Chouinard, M., and Corvera, S. (2011). Adenylate kinase 2 links mitochondrial energy metabolism to the induction of the unfolded protein response. J. Biol. Chem. *286*, 4081–4089. https://doi.org/10.1074/jbc.M110.134106.

41. Six, E., Lagresle-Peyrou, C., Susini, S., De Chappedelaine, C., Sigrist, N., Sadek, H., Chouteau, M., Cagnard, N., Fontenay, M., Hermine, O., et al. (2015). AK2 deficiency compromises the mitochondrial energy metabolism required for differentiation of human neutrophil and lymphoid lineages. Cell Death Dis. *6*, e1856. https://doi.org/10.1038/cddis.2015.211.

42. Single, B., Leist, M., and Nicotera, P. (1998). Simultaneous release of adenylate kinase and cytochrome c in cell death. Cell Death Differ. *5*, 1001–1003. https://doi.org/10.1038/sj.cdd.4400462.

43. Köhler, C., Gahm, A., Noma, T., Nakazawa, A., Orrenius, S., and Zhivotovsky, B. (1999). Release of adenylate kinase 2 from the mitochondrial intermembrane space during apoptosis. FEBS Lett. *447*, 10–12. https://doi.org/10.1016/s0014-5793(99)00251-3.

44. Lee, H.J., Pyo, J.O., Oh, Y., Kim, H.J., Hong, S.H., Jeon, Y.J., Kim, H., Cho, D.H., Woo, H.N., Song, S., et al. (2007). AK2 activates a novel apoptotic pathway through formation of a complex with FADD and caspase-10. Nat. Cell Biol. *9*, 1303–1310. https://doi.org/10.1038/ncb1650.

45. Zhang, S., Yamada, S., Park, S., Klepinin, A., Kaambre, T., Terzic, A., and Dzeja, P. (2021). Adenylate kinase AK2 isoform integral in embryo and adult heart homeostasis. Biochem. Biophys. Res. Commun. *546*, 59–64. https://doi.org/10.1016/j.bbrc.2021.01.097.

46. Lagresle-Peyrou, C., Six, E.M., Picard, C., Rieux-Laucat, F., Michel, V., Ditadi, A., Demerens-de Chappedelaine, C., Morillon, E., Valensi, F., Simon-Stoos, K.L., et al. (2009). Human adenylate kinase 2 deficiency causes a profound hematopoietic defect associated with sensorineural deafness. Nat. Genet. *41*, 106–111. https://doi.org/10.1038/ng.278.

47. Pannicke, U., Hönig, M., Hess, I., Friesen, C., Holzmann, K., Rump, E.M., Barth, T.F., Rojewski, M.T., Schulz, A., Boehm, T., et al. (2009). Reticular dysgenesis (aleukocytosis) is caused by mutations in the gene encoding mitochondrial adenylate kinase 2. Nat. Genet. *41*, 101–105. https://doi.org/10.1038/ng.265.

48. Romero-Tamayo, S., Laplaza, R., Velazquez-Campoy, A., Villanueva, R., Medina, M., and Ferreira, P. (2021). W196 and the beta-Hairpin Motif Modulate the Redox Switch of Conformation and the Biomolecular Interaction Network of the Apoptosis-Inducing Factor. Oxid. Med. Cell. Longev. *2021*, 6673661. https://doi.org/10.1155/2021/6673661.

49. Stocchi, V., Cucchiarini, L., Magnani, M., Chiarantini, L., Palma, P., and Crescentini, G. (1985). Simultaneous extraction and reverse-phase high-performance liquid chromatographic determination of adenine and pyridine nucleotides in human red blood cells. Anal. Biochem. *146*, 118–124. https://doi.org/10.1016/0003-2697(85)90405-1.

50. Lu, W., Wang, L., Chen, L., Hui, S., and Rabinowitz, J.D. (2018). Extraction and Quantitation of Nicotinamide Adenine Dinucleotide Redox Cofactors. Antioxid. Redox Signal. *28*, 167–179. https://doi.org/10.1089/ars.2017.7014.

51. Sallin, O., Reymond, L., Gondrand, C., Raith, F., Koch, B., and Johnsson, K. (2018). Semisynthetic biosensors for mapping cellular concentrations of nicotinamide adenine dinucleotides. eLife *7*, e32638. https://doi.org/10.7554/eLife.32638.

52. Cambronne, X.A., Stewart, M.L., Kim, D., Jones-Brunette, A.M., Morgan, R.K., Farrens, D.L., Cohen, M.S., and Goodman, R.H. (2016). Biosensor reveals multiple sources for mitochondrial NAD(+). Science *352*, 1474–1477. https://doi.org/10.1126/science.aad5168.

53. Yang, H., Yang, T., Baur, J.A., Perez, E., Matsui, T., Carmona, J.J., Lamming, D.W., Souza-Pinto, N.C., Bohr, V.A., Rosenzweig, A., et al. (2007). Nutrient-sensitive mitochondrial NAD^+ levels dictate cell survival. Cell *130*, 1095–1107. https://doi.org/10.1016/j.cell.2007.07.035.

54. Finger, Y., Habich, M., Gerlich, S., Urbanczyk, S., van de Logt, E., Koch, J., Schu, L., Lapacz, K.J., Ali, M., Petrungaro, C., et al. (2020). Proteasomal degradation induced by DPP9-mediated processing competes with mitochondrial protein import. EMBO J. *39*, e103889. https://doi.org/10.15252/embj.2019103889.

55. Soboll, S., Scholz, R., and Heldt, H.W. (1978). Subcellular metabolite concentrations. Dependence of mitochondrial and cytosolic ATP systems on the metabolic state of perfused rat liver. Eur. J. Biochem. *87*, 377–390. https://doi.org/10.1111/j.1432-1033.1978.tb12387.x.

56. Perez-Riverol, Y., Bai, J., Bandla, C., Garcia-Seisdedos, D., Hewapathirana, S., Kamatchinathan, S., Kundu, D.J., Prakash, A., Frericks-Zipper, A., Eisenacher, M., et al. (2022). The PRIDE database resources in 2022: a hub for mass spectrometry-based proteomics evidences. Nucleic Acids Res. *50*, D543–D552. https://doi.org/10.1093/nar/gkab1038.

57. Emsley, P., Lohkamp, B., Scott, W.G., and Cowtan, K. (2010). Features and development of Coot. Acta Crystallogr. D Biol. Crystallogr. *66*, 486–501. https://doi.org/10.1107/S0907444910007493.

58. Punjani, A., Rubinstein, J.L., Fleet, D.J., and Brubaker, M.A. (2017). cryoSPARC: algorithms for rapid unsupervised cryo-EM structure determination. Nat. Methods *14*, 290–296. https://doi.org/10.1038/nmeth.4169.

59. Liebschner, D., Afonine, P.V., Baker, M.L., Bunkóczi, G., Chen, V.B., Croll, T.I., Hintze, B., Hung, L.W., Jain, S., McCoy, A.J., et al. (2019). Macromolecular structure determination using X-rays, neutrons and electrons: recent developments in Phenix. Acta Crystallogr. D Struct. Biol. *75*, 861–877. https://doi.org/10.1107/S2059798319011471.

60. Bepler, T., Morin, A., Rapp, M., Brasch, J., Shapiro, L., Noble, A.J., and Berger, B. (2019). Positive-unlabeled convolutional neural networks for particle picking in cryo-electron micrographs. Nat. Methods *16*, 1153–1160. https://doi.org/10.1038/s41592-019-0575-8.

61. Ran, F.A., Hsu, P.D., Wright, J., Agarwala, V., Scott, D.A., and Zhang, F. (2013). Genome engineering using the CRISPR-Cas9 system. Nat. Protoc. *8*, 2281–2308. https://doi.org/10.1038/nprot.2013.143.

62. Stroud, D.A., Surgenor, E.E., Formosa, L.E., Reljic, B., Frazier, A.E., Dibley, M.G., Osellame, L.D., Stait, T., Beilharz, T.H., Thorburn, D.R., et al. (2016). Accessory subunits are integral for assembly and function of human mitochondrial complex I. Nature *538*, 123–126. https://doi.org/10.1038/nature19754.

63. Montague, T.G., Cruz, J.M., Gagnon, J.A., Church, G.M., and Valen, E. (2014). CHOPCHOP: a CRISPR/Cas9 and TALEN web tool for genome editing. Nucleic Acids Res. *42*, W401–W407. https://doi.org/10.1093/nar/gku410.

64. Küttner, V., Mack, C., Rigbolt, K.T.G., Kern, J.S., Schilling, O., Busch, H., Bruckner-Tuderman, L., and Dengjel, J. (2013). Global remodelling of cellular microenvironment due to loss of collagen VII. Mol. Syst. Biol. *9*, 657. https://doi.org/10.1038/msb.2013.17.

65. Cox, J., and Mann, M. (2008). MaxQuant enables high peptide identification rates, individualized p.p.b.-range mass accuracies and proteome-

wide protein quantification. Nat. Biotechnol. *26*, 1367–1372. https://doi.org/10.1038/nbt.1511.

66. Wischhof, L., Adhikari, A., Mondal, M., Marsal-Cots, A., Biernat, J., Mandelkow, E.M., Mandelkow, E., Ehninger, D., Nicotera, P., and Bano, D. (2022). Unbiased proteomic profiling reveals the IP3R modulator AHCYL1/IRBIT as a novel interactor of microtubule-associated protein tau. J. Biol. Chem. *298*, 101774. https://doi.org/10.1016/j.jbc.2022.101774.

67. Erdogan, A.J., Ali, M., Habich, M., Salscheider, S.L., Schu, L., Petrungaro, C., Thomas, L.W., Ashcroft, M., Leichert, L.I., Roma, L.P., et al. (2018). The mitochondrial oxidoreductase CHCHD4 is present in a semi-oxidized state in vivo. Redox Biol. *17*, 200–206. https://doi.org/10.1016/j.redox.2018.03.014.

68. Poepsel, S., Kasinath, V., and Nogales, E. (2018). Cryo-EM structures of PRC2 simultaneously engaged with two functionally distinct nucleosomes. Nat. Struct. Mol. Biol. *25*, 154–162. https://doi.org/10.1038/s41594-018-0023-y.

69. Sanchez-Garcia, R., Gomez-Blanco, J., Cuervo, A., Carazo, J.M., Sorzano, C.O.S., and Vargas, J. (2021). DeepEMhancer: a deep learning solution for cryo-EM volume post-processing. Commun. Biol. *4*, 874. https://doi.org/10.1038/s42003-021-02399-1.

70. Pettersen, E.F., Goddard, T.D., Huang, C.C., Meng, E.C., Couch, G.S., Croll, T.I., Morris, J.H., and Ferrin, T.E. (2021). UCSF ChimeraX: Structure visualization for researchers, educators, and developers. Protein Sci. *30*, 70–82. https://doi.org/10.1002/pro.3943.

71. Williams, C.J., Headd, J.J., Moriarty, N.W., Prisant, M.G., Videau, L.L., Deis, L.N., Verma, V., Keedy, D.A., Hintze, B.J., Chen, V.B., et al. (2018). MolProbity: More and better reference data for improved all-atom structure validation. Protein Sci. *27*, 293–315. https://doi.org/10.1002/pro.3330.

STAR★METHODS

KEY RESOURCES TABLE

REAGENT or RESOURCE	SOURCE	IDENTIFIER
**Antibodies**		
Goat anti-Mouse IgG (H&L), HRP Conjugate	ImmunoReagents	Cat# GtxMu-003-DHRPX
Goat anti-Rabbit IgG (H&L), HRP Conjugate	ImmunoReagents	Cat# GtxRb-003-DHRPX, RRID:AB_2884989
Rabbit polyclonal anti-AIFM1	Chemicon	Cat# ab16501
Rabbit polyclonal anti-AK2	Finger et al.[54]	N/A
Rabbit polyclonal anti-MIC19	Proteintech	Cat# 25625-1-AP, RRID:AB_2687533
Rabbit polyclonal anti-MIC27	Proteintech	Cat# 28514-1-AP, RRID:AB_3086061
Rabbit polyclonal anti-CPOX	St John's Laboratory	Cat# STJ23214
Rabbit polyclonal anti-HA	Sigma-Aldrich	Cat# SAB4300603, RRID:AB_10620829
Rabbit polyclonal anti-MIA40	Petrungaro et al.[22]	N/A
**Bacterial and virus strains**		
Rosetta2 (DE3)-AIFM1	Salscheider et al.[15]	N/A
Rosetta2 (DE3)-AK2A	This work	N/A
Rosetta2 (DE3)-AK2B	This work	N/A
Rosetta2 (DE3)-MIA40 SPS	This work	N/A
**Chemicals, peptides, and recombinant proteins**		
cycloheximide	Sigma	Cat# 239763, RRID:SCR_008988
DCIP	Sigma	Cat# D1878
FuGENE HD Transfection Reagent	Promega	Cat# E2311
Methyl-PEG-Maleimide, mmPEG24	Thermo Fisher	Cat# 22713
NADH	Sigma	Cat# N8129
MIA40 WT	This work	N/A
MIA40 YF->II	This work	N/A
AK2A WT	This work	N/A
AK2A F->L	This work	N/A
**Critical commercial assays**		
Pierce 660 nm Protein Assay Reagent	Thermo Scientific	Cat# 22660
ROTI®Quant universal	Carl Roth	Cat# 0120.1
**Deposited data**		
AIFM1-AK2A atomic coordinates	This work	PDB: 9GR0
AIFM1-dimer atomic coordinates	This work	PDB: 9GQY
AIFM1-MIA40 atomic coordinates	This work	PDB: 9GQZ
AIFM1-AK2A EM map	This work	EMD-51516
AIFM1-dimer EM map	This work	EMD-51514
AIFM1-MIA40 EM map	This work	EMD-51515
Proteomics	This work	PRIDE: PXD055617
**Experimental models: Cell lines**		
Flp-In T-Rex-293	Thermo Fisher	Cat# R78007
HEK293T	N/A	N/A
HeLa	N/A	N/A
HFF1	N/A	N/A
Jurkat	N/A	N/A
U87	N/A	N/A
HepG2	N/A	N/A

(*Continued on next page*)

Continued		
REAGENT or RESOURCE	SOURCE	IDENTIFIER
C2C12	Sigma	Cat# 91031101
Experimental models: Organisms/strains		
One Shot TOP10 Chemically Competent *E. coli*	Thermo Fisher	Cat# C404010
Rosetta2 (DE3) Competent *E. coli*	Novagen	Cat# 70954-4
Oligonucleotides		
DNA primers for cloning	This work	Table S1
Recombinant DNA		
pET-15(b)	Novagen	Cat# 69661-3
pET-24(a)	Novagen	Cat# 69749-3
pcDNA5/FRT/TO	Invitrogen	V652020
PB-CuO-MCSIRES-GFP-EF1-CymR-Puro	System Biosciences	Cat# PBQM812A-1
Myc-DDK	Origene	Cat# RC210614
Super PiggyBac Transposase Expression vector	System Biosciences	Cat# PB210PA-1
Software and algorithms		
ChimeraX (version 1.7.1)	N/A	https://www.cgl.ucsf.edu/chimerax
Coot (version 0.9.8.7)	Emsley et al.[57]	N/A
Corel draw	Corel Corporation	N/A
CryoSparc (version 4.4)	Punjani et al.[58]	N/A
Image Lab 5.2	Biorad Laboratories	N/A
Phenix (version 1.21)	Liebschner et al.[59]	N/A
TOPAZ	Bepler et al.[60]	N/A
Other		
UltrAuFoil® R 1.2/1.3 grid	Quantifoil	Cat# N1-A14nAu30-01

EXPERIMENTAL MODEL AND STUDY PARTICIPANT DETAILS

For cell lines and plasmids used in this study, see Table S1. For the generation of stable, inducible cell lines the HEK293 cell line–based Flp-In T-REx-293 cell line was used with the Flp-In T-REx system (Invitrogen). Cells were cultured in DMEM supplemented with 10% fetal bovine serum at 37°C under 5% CO_2.

METHOD DETAILS

Plasmids, cell lines, chemical treatments

For plasmids, cell lines, antibodies and further tools used in this study, see key resources table and Table S1. Cells were cultured in DMEM supplemented with 8% fetal calf serum (FCS) at 37°C under 5% CO_2. For cycloheximide (CHX) chase experiments, cells were treated with 100 μg/mL CHX dissolved in DMSO. For the generation of stable, inducible HEK293 cell lines, the Flp-In T-REx-293 cell line was used with the Flp-In T-REx system (Invitrogen). For the generation of stable, inducible HEK293T cell lines, the PiggyBac Transposon system (System Biosciences, BioCat) was used. Expression of constructs was induced using 1 μg/mL doxycycline (Flp-In T-Rex) or 30 μg/mL cumate (PiggyBac Transposon) for the indicated time points. Expression of SMAC^{MTS}-AIFM1-HA in HEK293T cells was induced with only 15 μg/mL to obtain comparable protein levels.

Generation of HEK293 knockout cells

HEK293 knockout cell lines were generated using the pSpCas9(BB)-2A-GFP (PX458) CRISPR/Cas9 construct (a gift from F. Zhang; Addgene, plasmid 4813; Ran et al.[61]) as described previously.[62] In brief, CRISPR/Cas9 gRNAs were designed for gene disruption using CHOPCHOP software.[63] Transfections were performed using Lipofectamine LTX (Thermo Fisher Scientific) and green fluorescent cells were individually sorted.

Immunoprecipitation

Immunoprecipitations were carried out under native lysis conditions. The cells were washed with PBS, supplemented with 20 mM NEM (N-Ethylmaleimide). After incubation in PBS supplemented with 20 mM NEM for 15 minutes, the cells were mechanically detached by scraping and sedimented with 800 x g for 5 minutes at 4°C. The cells were gently lysed in ice-cold native IP lysis buffer

(100 mM sodium phosphate pH 8.0, 100 mM sodium chloride, 1% [v/v] Triton X-100, 0.2 mM phenylmethylsulfonyl fluoride [PMSF]) for 1 h on ice. Lysate was cleared by centrifugation 22,000 x g for 1 h at 4°C. Supernatant was transferred to a prewashed agarose matrix and incubated for 3.5 to16 h at 4°C on a tumbling shaker. Afterwards, beads were triply washed with the IP lysis buffer, containing Triton X-100 and once finally washed with IP lysis buffer without Triton X-100. Precipitated proteins were eluted from the agarose matrix by addition of Laemmli buffer (2% sodium dodecyl sulfate [SDS], 60 mM Tris-HCl pH 6.8, 10% glycerol, 0.0025% bromophenol blue) and heating up to 96°C for 2 times 4 min.

SILAC-based mass spectrometry

The experiment was performed as described in Petrungaro et al.[22] Cells were subcultured and passaged in SILAC-DMEM (Thermo Fisher), supplemented with 10% dialyzed FBS (Gibco, Invitrogen), 1% L-glutamine (PAN Biotech), containing either L-arginine or L-arginine-13C6-15N4 (42 mg/L), and L-lysine or L-lysine-13C615N2 (73 mg/L), and 27.3 mg/L proline. After immunoprecipitation, samples were eluted in SDS-PAGE loading buffer containing 1 mM DTT (Dithiothreitol, Sigma-Aldrich) and alkylated using 5.5 mM iodoacetamide (Sigma-Aldrich). Protein mixtures were separated by SDS-PAGE, gel lanes were cut into 10 equal slices, proteins therein were in-gel digested with trypsin (Promega) and the resulting peptide mixtures were processed on STAGE tipps. Mass spectrometric measurements were performed on an LTQ Orbitrap XL mass spectrometer (Thermo Fisher Scientific) coupled to an Agilent 1200 nanoflow-HPLC (Agilent Technologies GmbH) as described.[64] The MS raw data files were uploaded into the MaxQuant software.[65] A full-length IPI human database containing common contaminants such as keratins and enzymes used for in-gel digestion was employed. Methionine oxidation, protein amino-terminal acetylation, carbamidomethyl cysteine and NEM cysteine were set as variable modifications. Double SILAC was chosen as quantitation mode. The MS/MS tolerance was set to 0.5 Da. Peptide lists were further used by MaxQuant to identify and relatively quantify proteins using the following parameters: peptide, and protein false discovery rates (FDR) were set to 0.01, maximum peptide posterior error probability (PEP) was set to 0.1, minimum peptide length was set to 6, minimum number peptides for identification and quantitation of proteins was set to one which must be unique, and identified proteins have been re-quantified.

Interaction profiling by using microchips

To find interactors of AIFM1, a high-content protein-protein microarray was performed using ProtoArray™ Human Protein Microarrays v5.1 (Thermo Fisher Scientific) containing ~9.000 N-terminal Glutathione S-Transferase (GST)-tagged human proteins extracted from transfected insect cells. As described in one of our prior studies,[66] each ProtoArray™ plate was placed at 4°C for equilibration for at least 15 minutes prior to blocking. Plates were then blocked using 5 mL blocking solution (50 mM HEPES, 200 mM NaCl, 0.08% Triton X-100, 25% glycerol, 20 mM glutathione, 1.0 mM DTT, 1X Synthetic Block) at 4°C for 1 h on a shaker at 50 rpm. After incubation, the blocking solution was aspirated and plates were incubated with recombinant AIFM1 protein (concentration of 5 ng/mL and 50 ng/mL) diluted in probe buffer (1X PBS, 0.1% Tween-20, 1X Synthetic Block), while one microarray (negative control) was exposed only to probe buffer for 90 min at 4°C. Afterwards, microplates were washed 5 times for 5 min with wash buffer (1X PBS, 1X Synthetic Block, 0.1% Tween 20). After washing, microplates were incubated with primary antibody in probe buffer for 90 min at 4°C, washed 5 times in probe buffer and incubated with Alexa Fluor™ 647-conjugated goat anti-rabbit IgG (Thermo Fisher Scientific, #A21244: Lot 1654324, 1 µg/mL in probe buffer) for 90 min at 4°C. Plates were then washed 5 times 5 min with wash buffer. To remove the residual salt, each plate was quickly washed with distilled water and dried by centrifuging at 200 g for 1 min. ProtoArray™ plates were scanned using an Axon 4000B fluorescent microarray scanner (Molecular Devices). Hits were considered based on the following criteria: (a) the fluorescent intensity value of the hits should be at least 20-fold higher than the corresponding negative control; (b) the normalized fluorescent signal was greater than 3 standard deviations; (c) the signal-to-noise ratio was higher than 0.5 and (d) the replicate spot coefficient of variation (CV) was lower than 65%.

Immunoblotting and image acquisition

Samples were prepared in Laemmli buffer containing 50 mM DTT, and heat denatured for 5 min at 96°C and DNA degraded by sonification (50% amplitude, 16 cycles). Protein samples were analyzed by SDS-PAGE and immunoblotting. The addition of 2,2,2-trichloroethanol (TCE) to the SDS-PAGE gel allowed for visualization of proteins and as a loading control. The immunoblotting images were detected using the ChemiDoc Touch Imaging system (Bio-Rad).

In vitro AK2 activity assay

The activity assay was carried out by coupling the AK2 reaction to hexokinase (HK) and glucose-6-phosphate dehydrogenase (G6PDH, HK/G6PDH mix from Roche). In this assay, AK2 provides the ATP for glucose phosphorylation by HK, followed by $NADP^+$ reduction to NADPH and an increase in absorbance at 340 nm. The reaction conditions were as follows: 58 mM glycylglycine pH 7.4, 10 mM $MgCl_2$, 0.006% BSA, 0.25 mM $NADP^+$, 20 mM glucose. The concentration of AK2 was set to 8 nM. All measurements were performed in triplicates in 96-well plates and read in a CLARIOstar microplate reader set to 25°C. A measurement without AK2 was performed simultaneously with all measurements to allow subtraction of the background reaction.

Analytical size-exclusion chromatography

Analytical size-exclusion chromatography was performed under native conditions to examine protein complexes between intact proteins. Cells were washed with 1x PBS and mechanically detached by scraping. Cells were sedimented at 500 g for 5 min. Pellets were resolved in 660 μL native lysis buffer (100 mM sodium phosphate pH 8.0, 100 mM sodium chloride, 1% (v/v) Triton X-100), supplemented with 0.2 mM PMSF. Cells were lysed for 1 h on ice, and the lysate was cleared by centrifugation. Lysate was loaded on a HiLoad™ 16/600 Superdex 200 preparation grade gel filtration column and installed in a liquid chromatography system (Aekta Purifier) from GE Healthcare. Eluted fractions were subjected to TCA precipitation, resuspended in loading buffer containing SDS and DTT, and analyzed by immunoblotting or label-free proteomics. A protein size standard was used as a reference, covering a range from 1.35 kDa to 670 kDa (#1511901, Bio-Rad).

Thiol shift assay to differentiate AK2 isoforms

The thiol shift assay was performed as previously described.[67] Cells were lysed in Laemmli buffer and oxidized cysteines were reduced by the addition of 10 mM TCEP (Tris(2-carboxyethyl)phosphine) and incubated at 96°C for 10 min. Following, the newly reduced cysteines were modified with the alkylating agent mmPEG24. Alkylation with 15 mM mmPEG24 was carried out for 1 h at room temperature. Subsequently, samples were separated by SDS–PAGE and analyzed by western blot, followed by immunoblotting.

Peptide synthesis

The peptides SYSRQEGKDRIIFVTKEDHETPSSAELVA-NH2 (MW_{calc} = 3292.58 Da; MW_{exp} = 3293.20, final purity 91 %), SICRQEGKDRIILVTKEDHETPSSAELVA-NH2 (MW_{calc} = 3224.61 Da; MW_{exp} = 3325.74, final purity 78 %), ATSKDLVMFI-NH2 (MW_{calc} = 1123.36, MW_{exp} = 1124.09 final purity 98 %) and ATSKDLVMLI-NH2 (MW_{calc} = 1090.35, MW_{exp} = 1090.08, final purity 98 %) were synthesized by solid-phase peptide synthesis on a peptide synthesizer (Syro I, MultiSynTech) using the fluorenylmethoxycarbonyl (Fmoc)/*tert*-butyl strategy on a Rink amide resin (0.48 mmol/g, 15 μmol scale). The amino acid coupling steps were performed twice for each amino acid using eight equivalents each of OxymaPure (2-cyano-2(hydroxyamino)acetate), DIC (dicyclohexylcarbodiimide), and the respective Fmoc-protected amino acid in DMF (dimethylformamide). The protecting group was removed by incubating the resin first in 40% piperidine in DMF followed by 20% piperidine in DMF. Peptides were cleaved from the resin using a mixture of trifluoroacetic acid/ thioanisole/ 1, 2-ethanedithiol (90:7:3, v/v/v). The crude peptides were purified by reverse-phase HPLC using a linear gradient of 10–60% B in A (A: water/0.1 % TFA; B: acetonitrile (ACN)/0.1 % TFA) over 45 min.

Protein purification

Recombinant proteins were expressed from the indicated plasmids (Table S1) in Rosetta2 *E. coli* strains. Bacterial growth was conducted in LB media (for AIFM1 supplemented with riboflavin and FAD) shaking at 37°C and 180 rpm. AIFM1(103-613) expression was induced with 1.0 mM IPTG and incubated for further 16 h before harvesting. AK2A C40,232S and AK2B C40S expression was induced with 0.1 mM IPTG and incubated further 16 h at 25°C. MIA40 C4S,C53S,C55S expression was induced with 0.5 mM and incubated for further 3 h. Cells were harvested on ice in PBS and stored at -20°C. The 6xHis-tagged constructs were purified by Immobilized Metal Affinity Chromatography using Ni Sepharose (6 Fast Flow, GE). The bacterial lysate was bound to beads in binding buffer supplemented with 10 mM imidazole at 4°C. Beads were washed with binding buffer supplemented with 20 mM imidazole prior to elution with 150 mM imidazole. Imidazole was removed using PD-10 columns (Cytiva) and the proteins stored at 4°C.

In vitro complex reconstitution

The AIFM1 AK2A or MIA40 complex, respectively, was reconstituted by combining the recombinant proteins in a 1:2 to 1:4 (AIFM1: AK2A/MIA40) molar ratio in 100 mM NaCl, 20 mM Tris/CL pH = 7.4 in presence of 0.1 mM NADH. After incubation for 20 minutes and centrifugation, the respective complex was separated from monomeric proteins on a 16/600 Superdex 200 PG or on a Superdex 200 Increase 10/300 GL column. All steps were performed at 4°C.

Isothermal titration calorimetry (ITC)

Isothermal titration calorimetry was performed at 25°C on a MicroCal Auto-ITC200 (Malvern, United Kingdom). For analysis of the interaction between AIFM1 and peptides, 6xHis-tagged AIFM1(103–613) was dialyzed against PBS pH 7.4 supplemented with 0.1 mM NADH at 4°C for 17 h. Lyophilized peptide was dissolved in dialysis buffer to a final concentration of 250 - 300 μM. Ligand proteins were dialyzed in the same batch of buffer as AIFM1(103-613) and used in a concentration of 250 – 300 μM. The concentration of receptor in the sample cell was 30 μM. Measurements were carried out by 2 μl injections of the peptide into the cell with an injection duration of 4 s. Ultimately, 19 injections were performed during the titration.

Cryo-EM grid preparation and data collection

Before cryo-EM, sample quality was assessed by negative staining electron microscopy as previously described[68] (data not shown). For cryo-grid preparation, 3 μL of purified protein was applied to an UltrAuFoil® R 1.2/1.3 grid (Quantifoil) that had been

glow-discharged for 1 minute and 45 seconds. The grids were then blotted for 4 seconds at 100% humidity and 8°C, followed by plunging in liquid ethane cooled by liquid nitrogen using a Vitrobot Mark IV (Thermo Fisher Scientific). The prepared grids were stored in liquid nitrogen until use.

Cryo-EM data for AIFM1-MIA40 were collected using a Titan Krios G3i (Thermo Fisher Scientific), while data for AIFM1 dimer and AIFM1 with AK2 were collected using Titan Krios G4 (Thermo Fisher Scientific), all operated at 300 kV with a 35° tilt to overcome preferred orientation using EPU (Thermo Fisher Scientific).

Two datasets were collected for AIFM1-MIA40: 1907 raw movies for the first dataset and 2084 for the second, using a Falcon III direct electron detector with a pixel size of 0.654 Å/pixel. The total electron dose was 50.82 e/Å^2 for the first dataset and 50.57 e/Å^2 for the second, distributed over 48 frames with a defocus range of -0.6 to -2.6 μm.

For the AIFM1 dimer, 15,440 raw movies with a pixel size of 0.46 Å/pixel and for AIFM1 with AK2A, 4542 movies with a pixel size of 0.58 Å/pixel were collected using a Falcon 4i equipped with a Selectris energy filter (Thermo Fisher Scientific). These movies were stored in electron-event representation (EER) format, with a total dose of 50 e/Å^2 distributed over 468 frames and a defocus range of -0.7 to -1.7 μm.

Cryo-EM data processing

All datasets were processed using cryoSPARC (V4.4).[58] The workflows are described in detail in Figures S6–S8. In summary, the movies were pre-processed with patch-based motion correction and CTF estimation. Initial blob picking and 2D classification were performed to identify classes with distinguishable features representing stacks of intact particles, which were then used to train the TOPAZ picker.[60] After further 2D classification, particles were divided into high- and low-defocus groups for the training of TOPAZ models suitable for picking particles from high- and low-defocus micrographs, respectively. Multiple iterations of 2D classification and multiple-class *ab initio* reconstructions were performed, each followed by TOPAZ model training, which yielded successively larger particle stacks giving rise to higher resolution 3D reconstructions. Inspection of the resulting 2D classes and reconstructions showed that this process sorted out particles not contributing to high-resolution reconstructions rather than heterogeneous conformations or complex compositions. Homogeneous or non-uniform refinements yielded the final, high-resolution reconstructions used for model building, as summarized in Figures S6–S8. For the AIFM1 dimer, two initial models containing 149,783 and 188,760 particles, respectively, were pooled, classified using *ab initio* reconstruction, and refined using non-uniform refinement. The initial models of the AIFM1-AK2A complex (229,483 particles) and the AIFM1-MIA40 complex (310,098 particles), the particles were subjected to another round of TOPAZ training, 2D classification, and ab initio reconstruction, followed by either non-uniform or homogeneous refinement.

Particles of AIFM1 with MIA40 were extracted with a box size of 420 pixels, while the AIFM1 dimer and AIFM1 with AK2A were extracted with a box size of 416 pixels. All refinements were eventually processed with Reference Motion correction resulting in resolutions of 2.8 Å for the AIFM1 dimer (225,162 particles), 2.4 Å for AIFM1 with MIA40 (291,656 particles) and 2.6 Å for AIFM1 with AK2A (307,496 particles). Statistics on data collection and validation are given in Figures S6–S8.

Model building and refinement

For all AIFM1 structures obtained in this study, an AlphaFold2 model was used to obtain an initial model and then cross-referenced with previously published human and mouse AIFM1 models (PDB:4BUR and PDB:3GD4). Residues were adjusted in Coot[57] and models were iteratively refined and adjusted using PHENIX (V1.21)[59] and Coot. For better visualization during initial model building, maps were processed with DeepEMhancer.[69] Maps and models were visualized using ChimeraX.[70] Statistics on data collection and validation reports were automatically generated using MolProbity within Phenix.[71]

DCIP activity assay

AIFM1 catalyzes the efficient reduction of NAD(P)H: 2,6 dichlorophenolindophenol (DCIP). In a two-step reaction, NADH first reduces the FAD cofactor in AIFM1, from which subsequently electrons are transferred onto DCIP. Using DCIP as the electron acceptor, the latter step is faster than the first one allowing the observation of AIFM1-dependent oxidation of NADH in dependence of differing NADH concentrations. The enzymatic activities of AIFM1 alone, reconstituted AIFM1-AK2A-, or AIFM1-MIA40-binding site peptide complexes as NAD(P)H:DCIP oxidoreductases were measured in 20 mM NaCl, 20 mM Tris/Cl pH = 7.4 at 25°C. Recombinant AIFM1 was used in a final concentration of 800 – 927 nM, The concentration of DCIP was kept constant at 200 μM and the indicated peptides at a final concentration of 3.3 μM. The reaction was started by adding NAD(P)H and the DCIP reduction monitored at 600 nm in a plate reader (CLARIOstar, BMG Labtech). The background reaction in the absence of AIFM1 for each NAD(P)H concentration was subtracted from the data.

Cell proliferation assay

For cell proliferation assay recorded with the cytosmart omni (Axion Biosystems), 15,000 cells were seeded on a poly-L-coated 48-well dish and incubated at 37°C. Expression of AK2 isoforms was continuously induced by doxycycline treatment. After 24 hours, the medium was exchanged with DMEM containing galactose (DMEM supplemented with 4.5 g/l galactose, 1 mM sodium pyruvate,

1 x nonessential amino acids, 10% FCS and 500 mg/ml Pen/Strep) or left in medium with glucose. Every day the medium was exchanged by removing 250 μl and adding 250 μl fresh DMEM containing galactose or glucose. Every 6h the coverage of each well was scanned for 6 days using the cytosmart omni.

QUANTIFICATION AND STATISTICAL ANALYSIS

The intensity of immunoblot signals was quantified using Image Lab (Biorad). Error bars in figures represent standard deviation. The number of experiments is reported in the figure legend.

Molecular Cell, Volume *85*

Supplemental information

Interaction with AK2A links AIFM1 to cellular energy metabolism

Robin Alexander Rothemann, Egor Pavlenko, Mrityunjoy Mondal, Sarah Gerlich, Pavel Grobushkin, Sebastian Mostert, Julia Racho, Konstantin Weiss, Dylan Stobbe, Katharina Stillger, Kim Lapacz, Silja Lucia Salscheider, Carmelina Petrungaro, Dan Ehninger, Thi Hoang Duong Nguyen, Jörn Dengjel, Ines Neundorf, Daniele Bano, Simon Poepsel, and Jan Riemer

SUPPLEMENTAL INFORMATION

Interaction with AK2A links AIFM1 to cellular energy metabolism

Robin Alexander Rothemann[1#], Egor Pavlenko[2#], Mrityunjoy Mondal[3], Sarah Gerlich[1], Pavel Grobushkin[1], Sebastian Mostert[1], Julia Racho[1], Konstantin Weiss[1], Dylan Stobbe[1], Katharina Stillger[4], Kim Lapacz[1], Silja Lucia Salscheider[1], Carmelina Petrungaro[1], Dan Ehninger[3], Thi Hoang Duong Nguyen[5], Jörn Dengjel[6], Ines Neundorf[4], Daniele Bano[3], Simon Poepsel[2,7,#,*], Jan Riemer[1,7,#,*,$]

1, Redox Metabolism Group, Institute for Biochemistry, University of Cologne, D-50674 Cologne, Germany

2, Center for Molecular Medicine Cologne (CMMC), Faculty of Medicine and University Hospital, University of Cologne, D-50931 Cologne, Germany.

3, German Center for Neurodegenerative Diseases (DZNE), D-53127 Bonn, Germany.

4, Peptide Biochemistry Group, Institute for Biochemistry, University of Cologne, D-50674 Cologne, Germany

5, MRC Laboratory of Molecular Biology, Cambridge, CB2 0QH, UK

6, Department of Biology, University of Fribourg, CH-1700 Fribourg, Switzerland

7, Cologne Excellence Cluster on Cellular Stress Responses in Aging-Associated Diseases (CECAD), University of Cologne, D-50931 Cologne, Germany.

#, equal contribution

§, lead contact: J.R., Jan Riemer

* address correspondence to

J.R.: jan.riemer@uni-koeln.de, +49-221-470-7306, ORCID 0000-0002-7574-8457,

S.P.: spoepsel@uni-koeln.de, +49-221-478-96987, ORCID 0000-0002-8304-4062

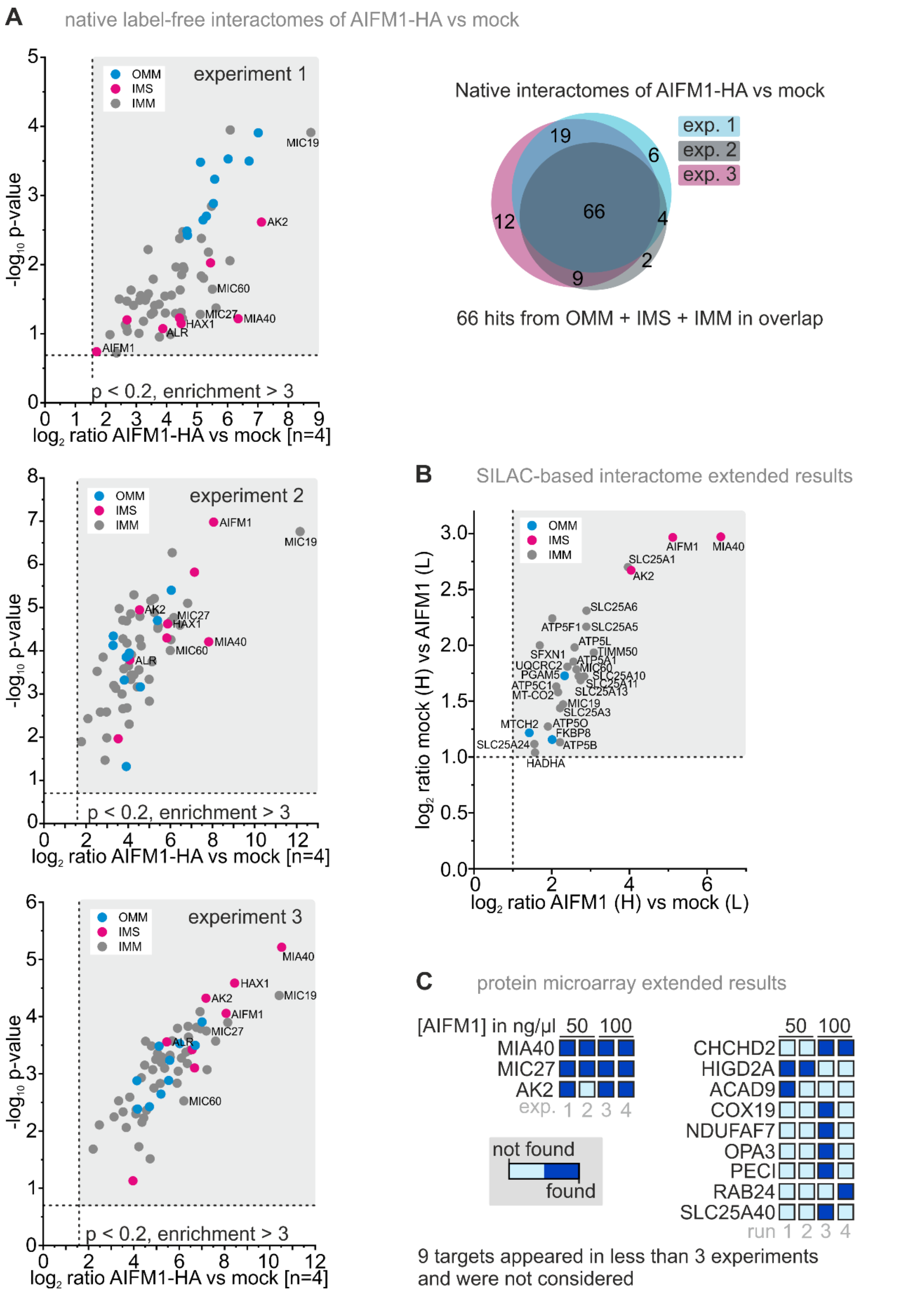

Figure S1: A high-confidence interactome reveals AK2 and MICOS components as novel AIFM1 interaction partners related to Figure 1.

(A) Individual data sets for the three repeats with four biological replicates each shown in **Figure 1C**. The interactomes show considerable overlap leading to the identification of 66 potential interactors of AIFM1-HA.

(B) SILAC-based data set for the experiment shown in **Figure 1D**. We identified 27 potential interactors localized in IMM, IMS and OMM. Notably, the datasets from (A) and (B) do not only show AK2, MIA40, and MICOS components as potential AIFM1 interactors but also members of the SLC25 family (including the ADP/ATP carrier SLC25A5) and the ATPase (including ATP5A1, ATP5B, ATP5F1, ATP5L, and ATP5O)

(C) Extended results for the protein microarray shown in **Figure 1E**. Many different targets were only identified in single experiments. Only AK2, MIA40 and MIC27 were consistently identified.

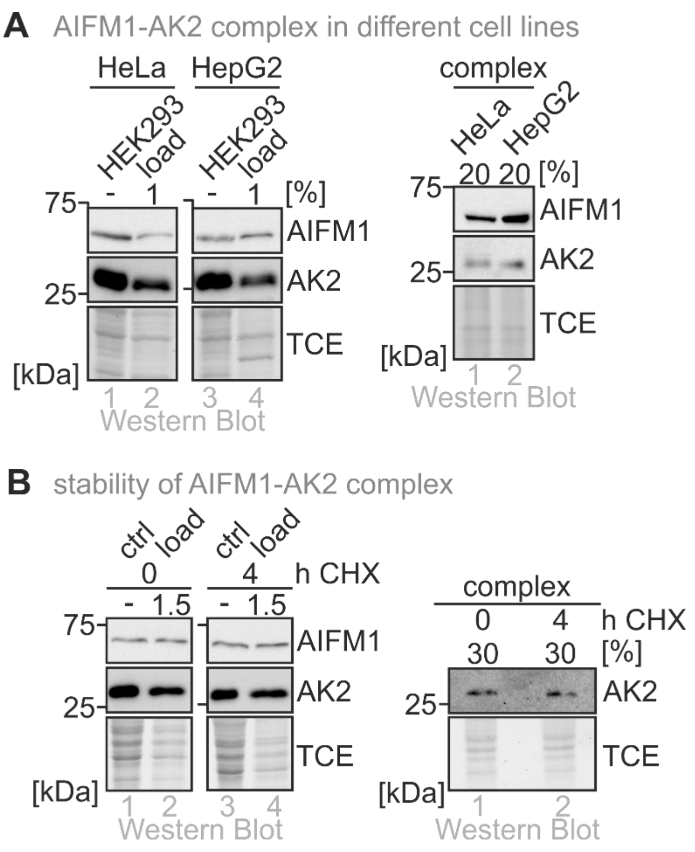

Figure S2: Properties of the AIFM1-AK2 complex related to Figure 2.

(A) The AIFM1-AK2A complex is present in different cell lines. The experiment was performed as in **Figure 2F** in the indicated cell lines.

(B) The AIFM1-AK2A complex is stable. The experiment was performed as in **Figure 2F** except that cells were treated with the translation inhibitor cycloheximide for 4 hours or left untreated. The amounts of AK2A in the AIFM1-AK2A complex do not change during this time indicating a stable complex.

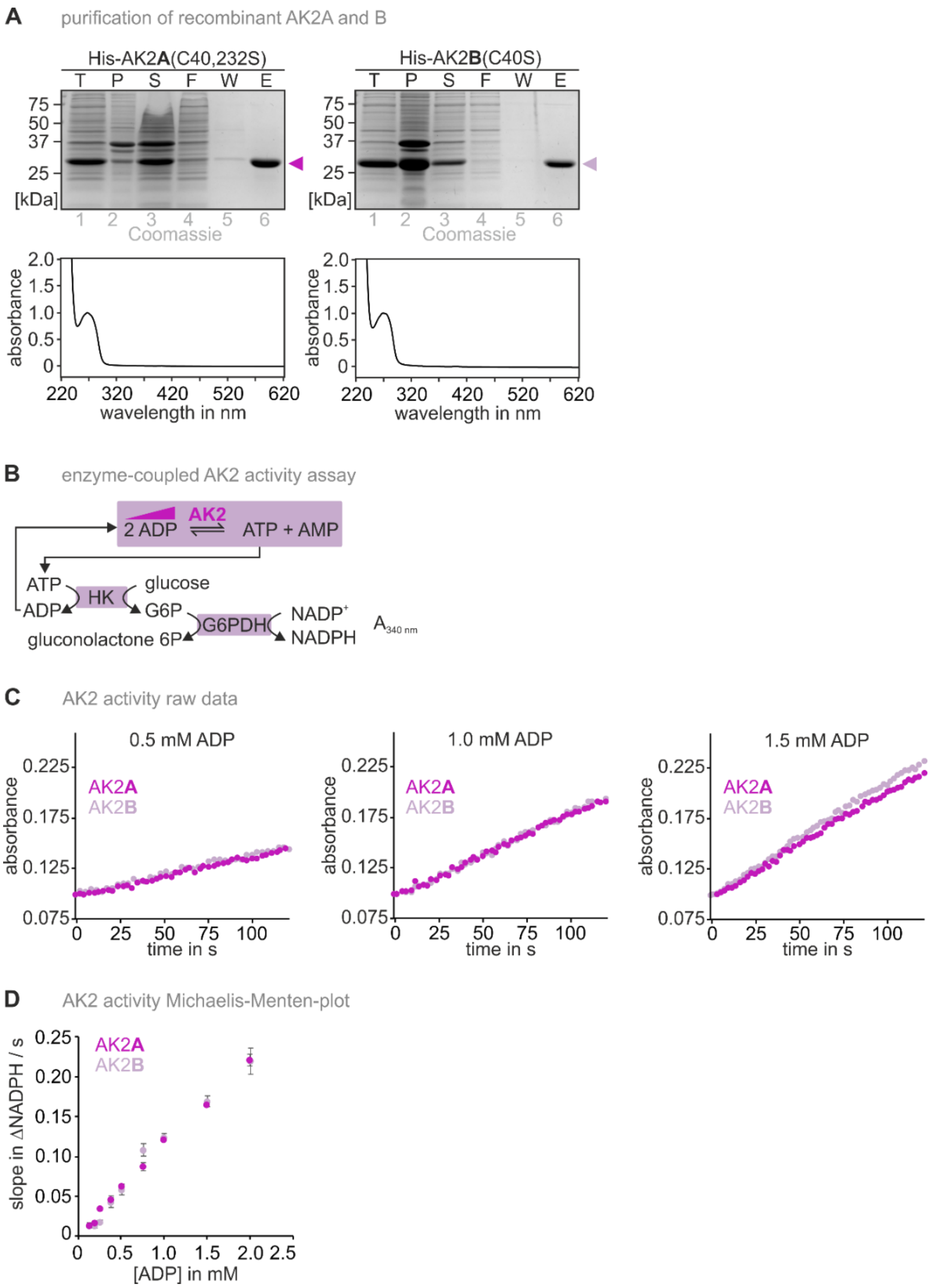

Figure S3: Recombinant AK2 isoform A and B exhibit a similar enzymatic profile related to Figure 2.

(A) Purification of the isoforms His-AK2A and His-AK2B. Both proteins were well-behaved and can be purified in similar amounts and to similar purity. T: total, P: pellet, S: supernatant, F: flow-through, W: wash, E: eluate

(B) AK2 activity assay with varying ADP concentrations. Hexokinase (HK) and glucose-6-phosphate dehydrogenase (G6PDH) couple the reduction of $NADP^+$ to the interconversion of adenine nucleotides. G6P, glucose-6-phosphate

(C,D) Raw data **(C)** and velocity *vs* ADP concentration slope plot **(D)** for the enzyme activities of AK2A and AK2B. The concentration of ADP is titrated. Both isoforms exhibit the same activity towards ADP.

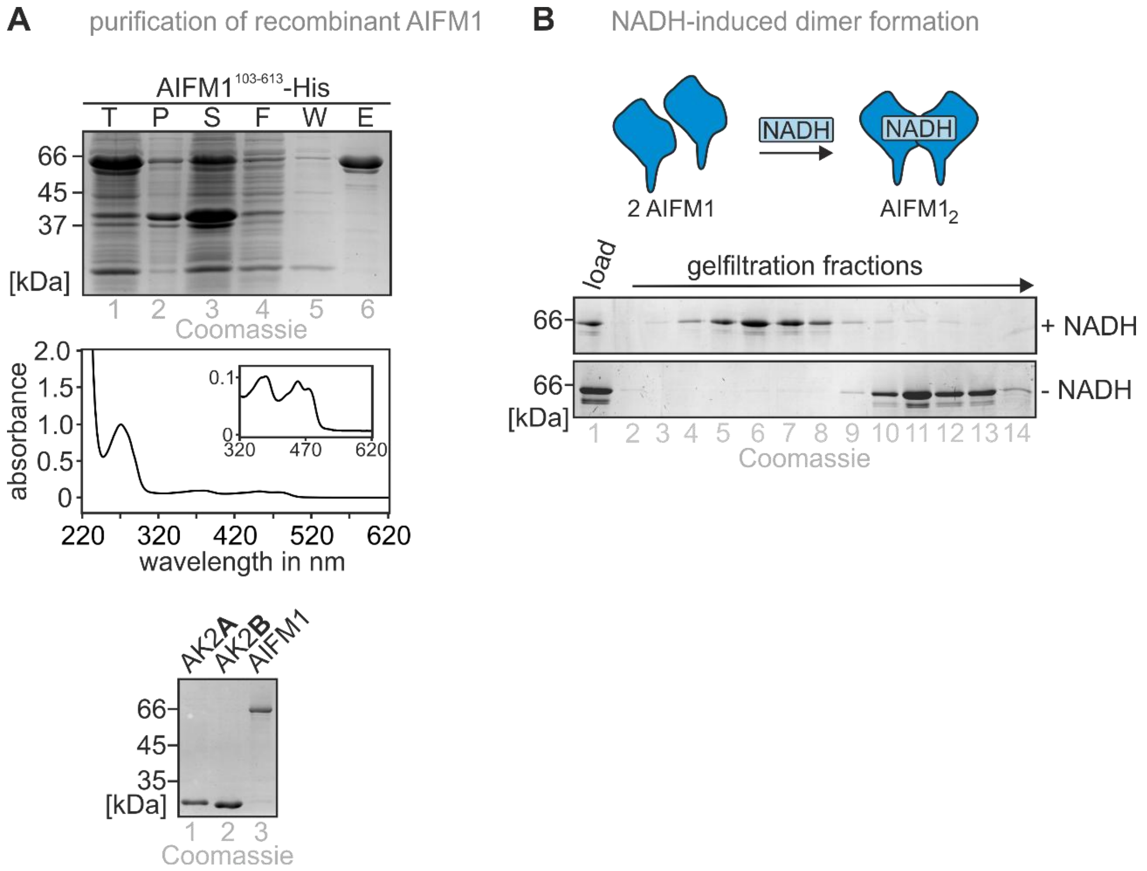

Figure S4: NADH-induced *in vitro* dimerization of recombinant soluble AIFM1 related to Figure 2.

(A) Purification of soluble AIFM1 (AIFM1 103-613). AIFM1, lacking the mitochondrial targeting signal and the transmembrane domain, was purified and contained the FAD cofactor. Purified AK2A and AK2B used in the *in vitro* reconstitution assay of the AIFM1-AK2 complex were loaded for comparison onto the same gel as AIFM1. T: total, P: pellet, S: supernatant, F: flow-through, W: wash, E: eluate

(B) NADH-induced dimer formation of AIFM1. NADH addition leads to rapid AIFM1 dimerization that can be followed by gel filtration. In the absence of NADH, AIFM1 migrates at the height of the monomer.

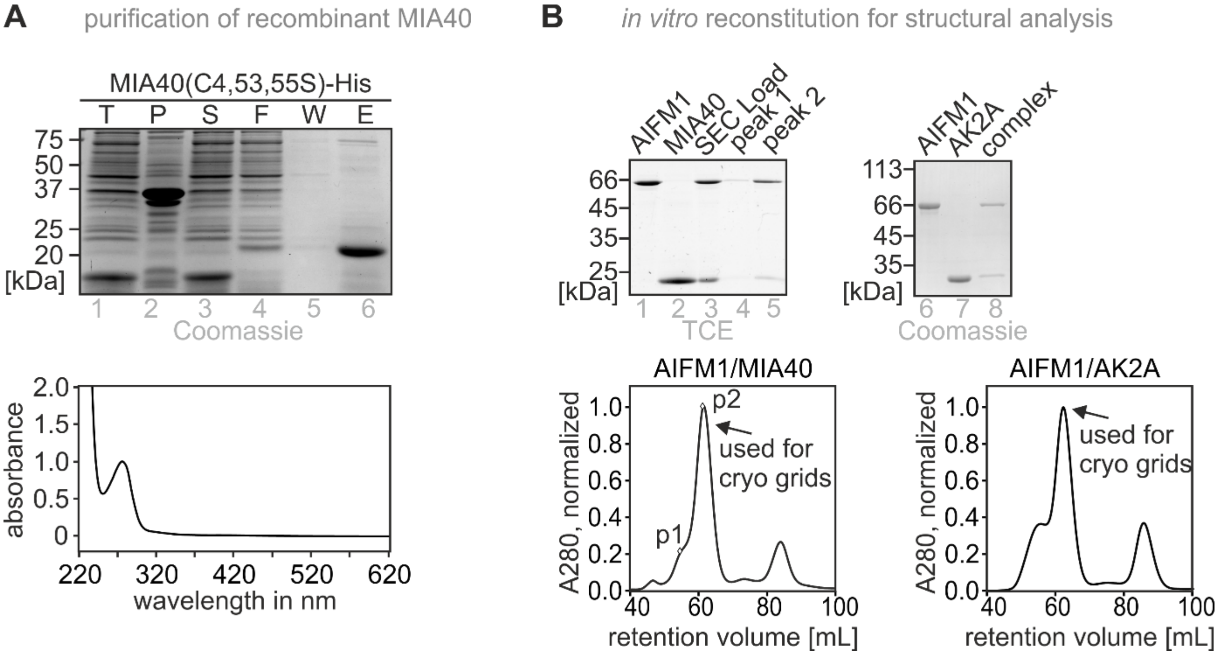

Figure S5: Protein preparation for cryo-EM related to Figure 3.

(A) Purification of the redox-inactive MIA40-C4,53,55S variant. T: total, P: pellet, S: supernatant, F: flow-through, W: wash, E: eluate

(B) *In vitro* reconstitution of the AIFM1-MIA40 and AIFM1-AK2A complexes and isolation of the complexes by gel filtration for cryo-EM.

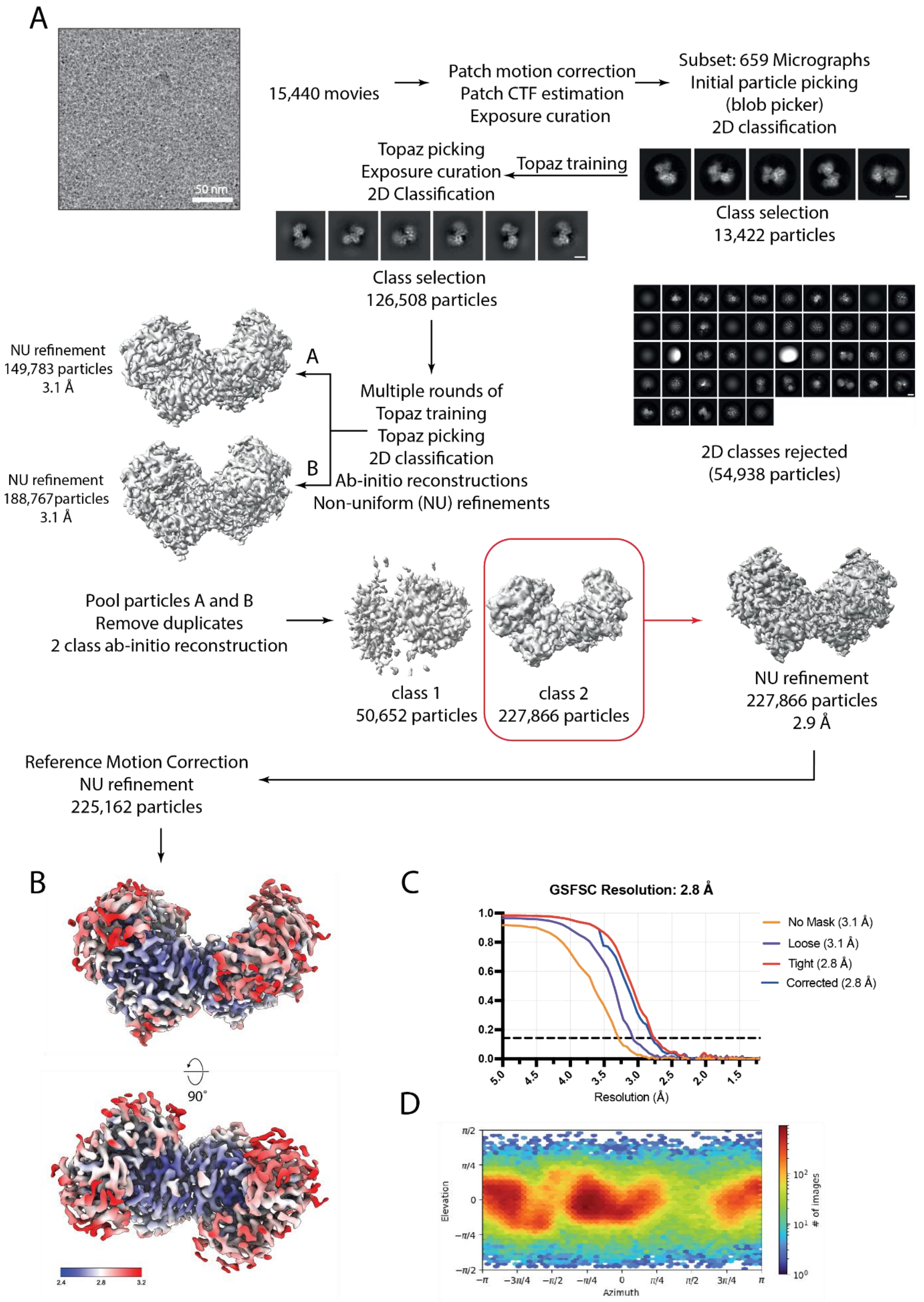

Figure S6: Cryo-EM data processing workflow of the AIFM1 dimer related to Figure 3.

(A) Processing workflow for the AIFM1 dimer. All data processing was performed in cryoSPARC [1]. The first model of the Topaz particle picker [2] was trained with a subset of particles identified by 2D

classification after using the blob picker. Several rounds of Topaz training with particles that gave rise to well-resolved 2D class averages were performed, successively increasing the number and quality of particle picks. Particle subsets were selected based on structural details visible in 2D class averages. *Ab-initio* reconstructions using multiple classes were performed to further remove particles not contributing to well-resolved 3D reconstructions. Similarly, before the last refinement, a new *ab-initio* reconstruction with two classes was performed, sorting out further particles that did not give rise to well-resolved reconstructions. Scale bars in 2D class averages are 5 nm.

(B) Final reconstruction colored according to local resolution, ranging from 2.4 Å (blue) to 3.2 Å (red).

(C) The global resolution determined by the FSC cut-off at 0.143 was 2.8 Å.

(D) Euler angle distribution plot, exported from cryoSPARC.

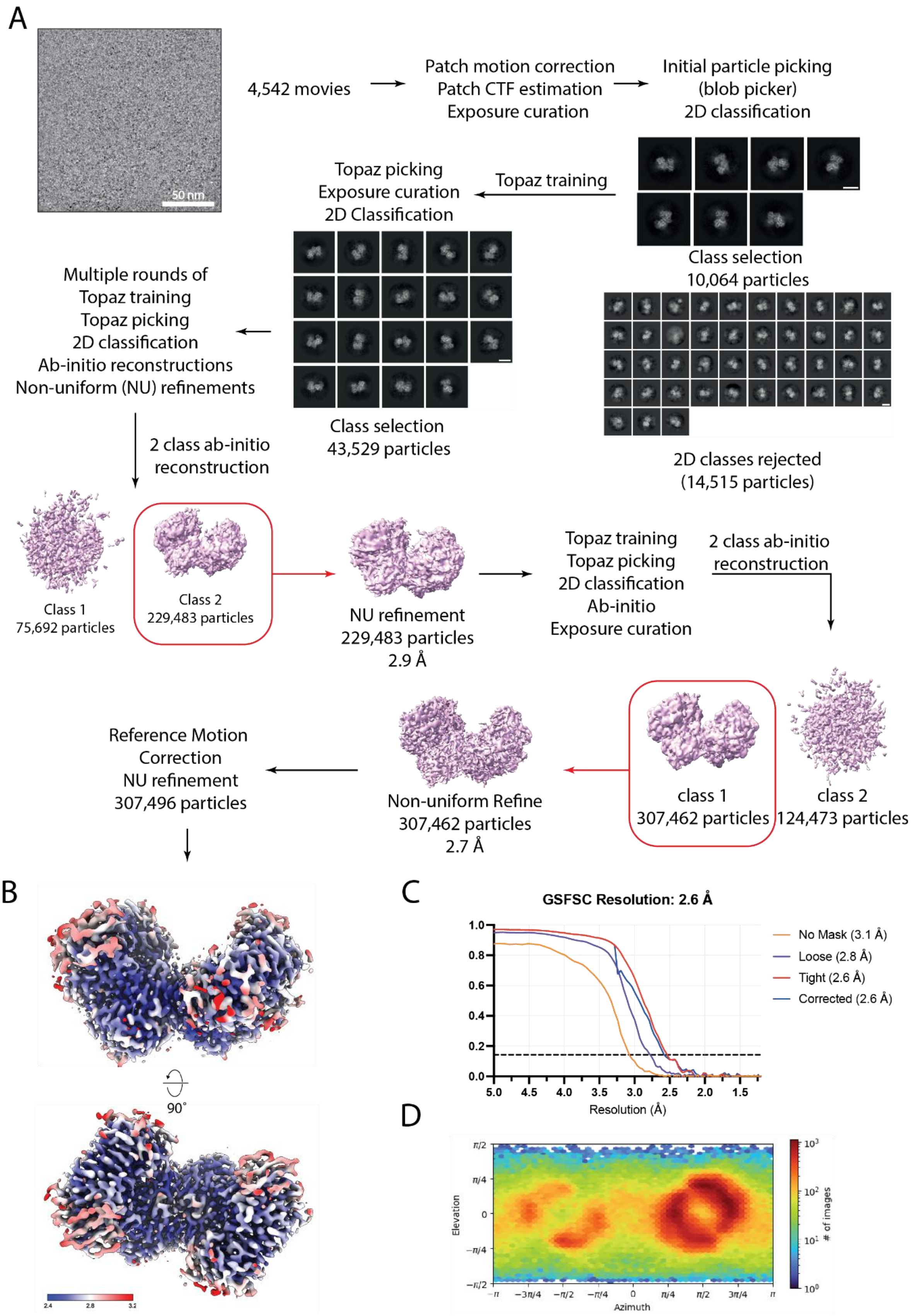

Figure S7: Cryo-EM data processing workflow of the AIFM1-AK2A complex related to Figure 3.

(A) Processing workflow for the AIFM1-AK2A complex. All data processing was performed in cryoSPARC [1]. The first model of the Topaz particle picker was trained with a subset of particles identified

by 2D classification after using the blob picker. Several rounds of Topaz training with particles that gave rise to well-resolved 2D class averages were performed, successively increasing the number and quality of particle picks. Particle subsets were selected on the basis of structural details visible in 2D class averages. *Ab-initio* reconstructions using multiple classes were performed to further remove particles not contributing to well-resolved 3D reconstructions. Similarly, before the last refinement, a new *ab-initio* reconstruction with two classes was performed, sorting out further particles that did not give rise to well-resolved reconstructions. Scale bars in 2D class averages are 5 nm.

(B) Final reconstruction colored according to local resolution, ranging from 2.4 Å (blue) to 3.2 Å (red).

(C) The global resolution determined by the FSC cut-off at 0.143 was 2.6 Å.

(D) Euler angle distribution plot, exported from cryoSPARC.

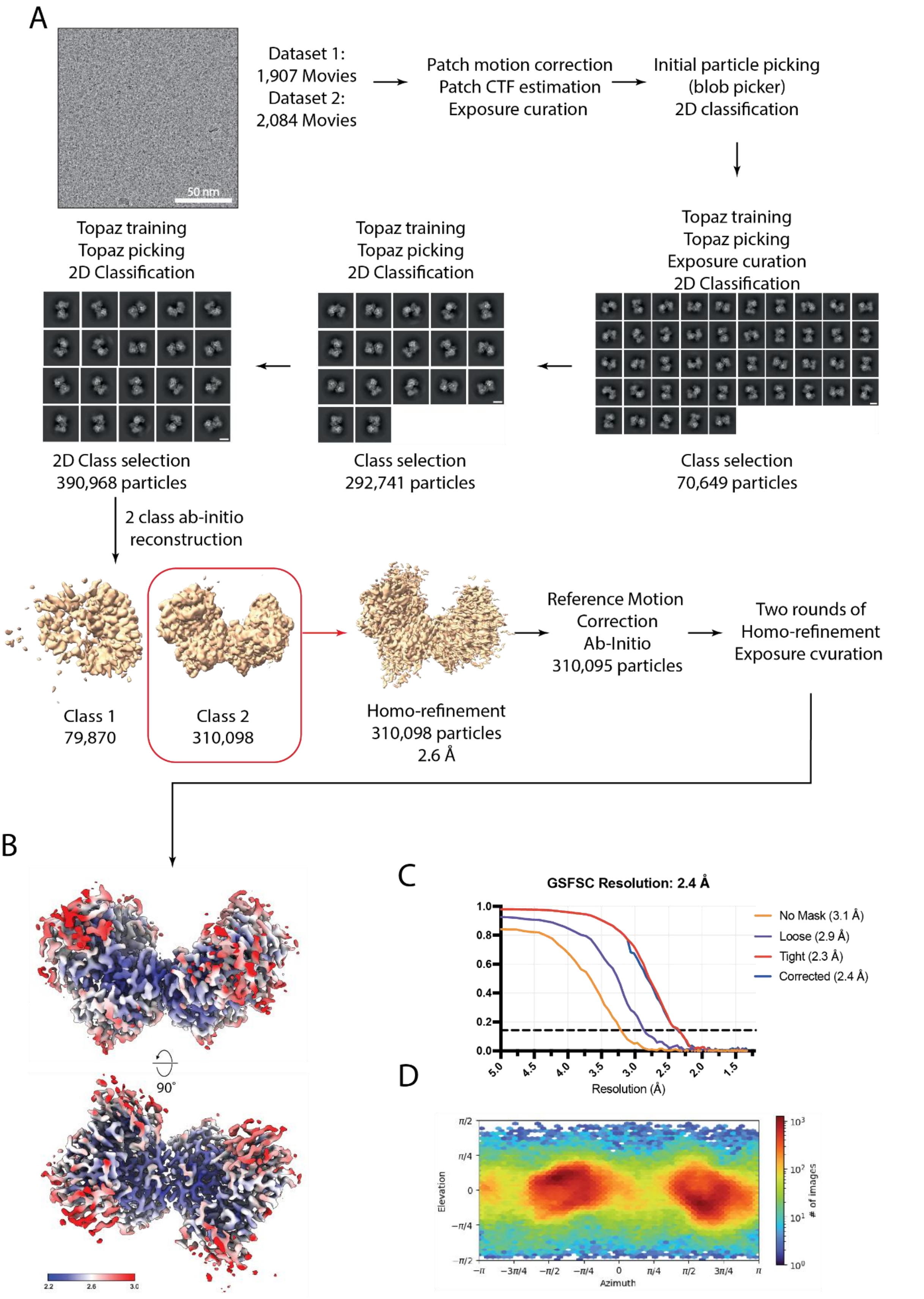

Figure S8: Cryo-EM data processing workflow of the AIFM1-MIA40 complex related to Figure 3.

(A) Processing workflow for the AIFM1-MIA40 complex. All data processing was performed in cryoSPARC [1]. The first model of the Topaz particle picker was trained with a subset of particles identified

by 2D classification after using the blob picker. Several rounds of Topaz training with particles that gave rise to well-resolved 2D class averages were performed, successively increasing the number and quality of particle picks. Particle subsets were selected based on structural details visible in 2D class averages. *Ab-initio* reconstructions using multiple classes were performed to further remove particles not contributing to well-resolved 3D reconstructions. Similarly, before the last refinement, a new *ab-initio* reconstruction with two classes was performed, sorting out further particles that did not give rise to well-resolved reconstructions. Scale bars in 2D class averages are 5 nm.

(B) Final reconstruction colored according to local resolution, ranging from 2.2 Å (blue) to 3.0 Å (red).

(C) The global resolution determined by the FSC cut-off at 0.143 was 2.4 Å.

(D) Euler angle distribution plot, exported from cryoSPARC.

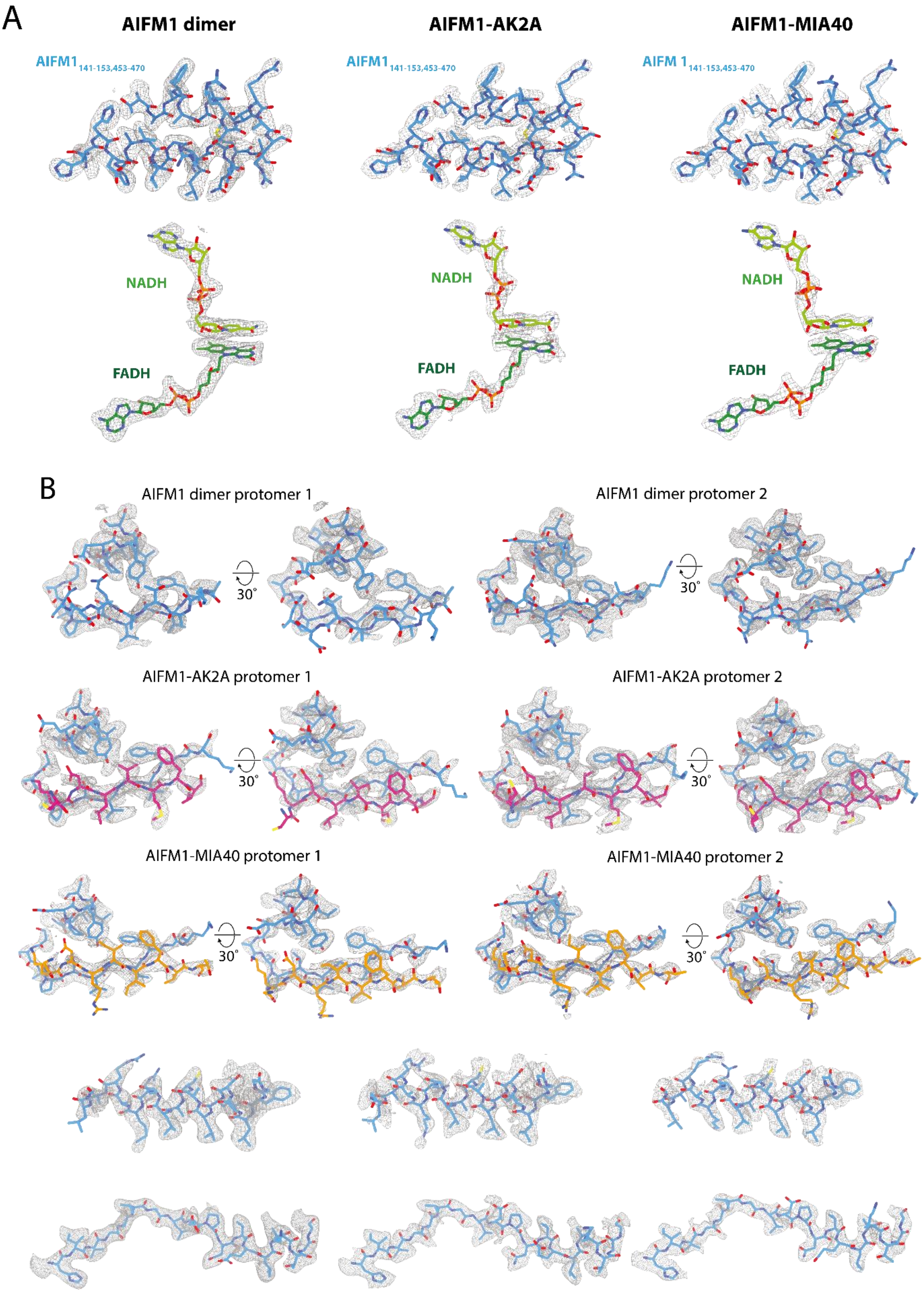

Figure S9: Cryo-EM map quality related to Figure 3.

(**A**) Exemplary cryo-EM densities (grey mesh) and atomic models obtained in this study for AIFM1 (aa 141-153,453-470), top panels, or the NAD and FAD cofactors (bottom panels), of all three structures shown here.

(B) Exemplary cryo-EM densities (grey mesh) and atomic models of the C-terminal domain of AIFM1 (aa 343-349 and 500-510, blue), AK2A (aa 232-239, blue), and MIA40 (aa 8-16, orange), shown for all three structures and both protomers of each dimer.

(C) Exemplary cryo-EM densities (grey mesh) and atomic models of AIFM1 aa 308-327, shown for the AIFM1 dimer (left), AIFM1-AK2A (middle) and AIFM1-MIA40 (right) models.

(D) Exemplary cryo-EM densities (grey mesh) and atomic models of AIFM1 aa 393-411, shown for the AIFM1 dimer (left), AIFM1-AK2A (middle) and AIFM1-MIA40 (right) models.

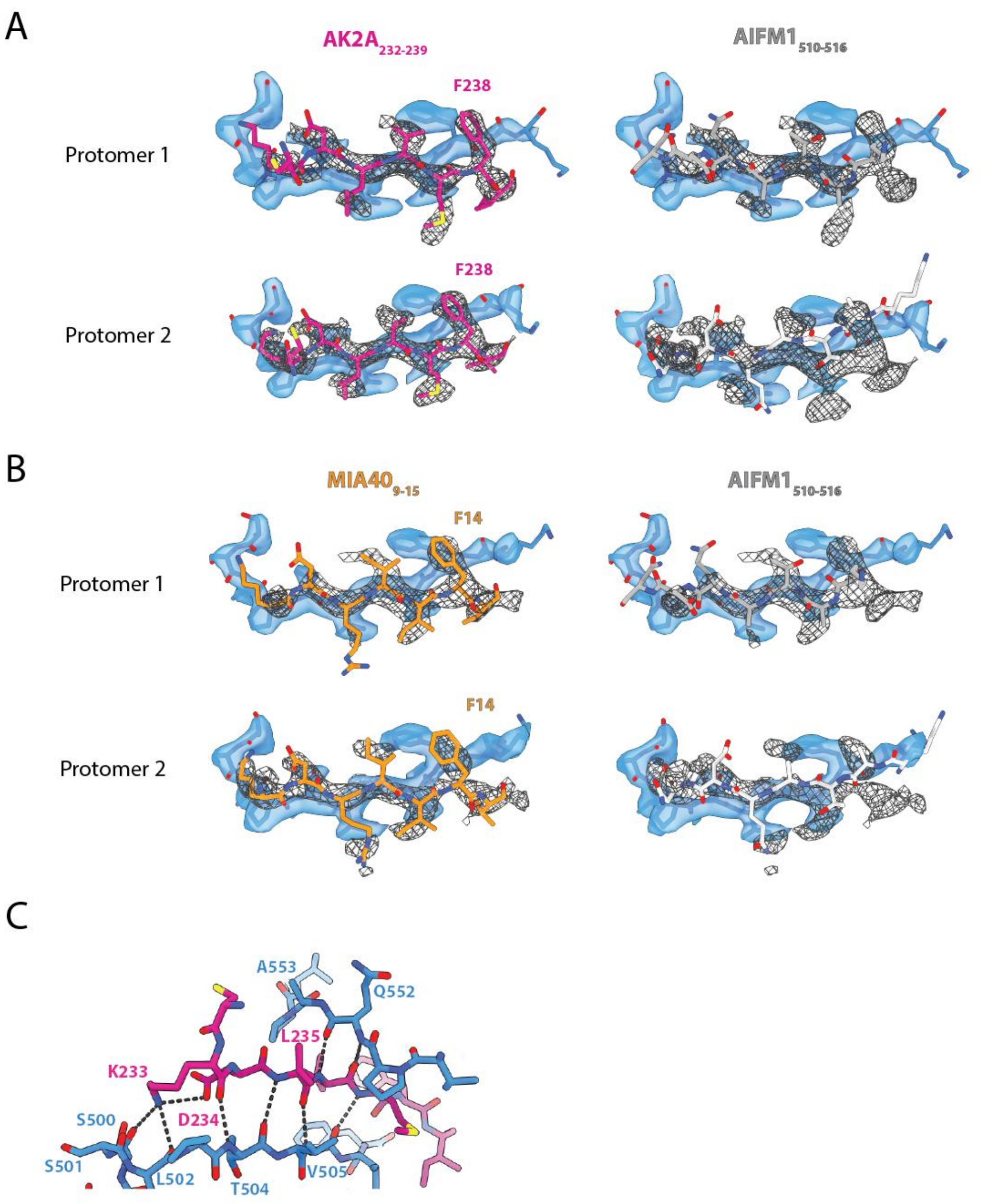

Figure S10: Details of AK2A and MIA40 interactions with AIFM1, unambiguous fit of AK2A and MIA40 related to Figure 3.

(A) Cryo-EM map of the AIFM1-AK2A complex and fitted model for AIFM1 (aa 500-510), (blue, transparent map) and AK2A (aa 232-239), (purple, grey mesh map, left panels) showing the fit with AK2A as opposed to AIFM1 (aa 510-516), (grey model, right panels).

(B) Cryo-EM map of the AIFM1-MIA40 complex and fitted model for AIFM1 (aa 500-510), (blue, transparent map) and MIA40 (aa 9-15), (orange, grey mesh map, left panels) showing the fit with MIA40 as opposed to AIFM1 (aa 510-516), (grey model, right panels).

(C) Details of the interactions of AK2A (purple) with the C-loop of AIFM1 (blue), and hydrogen bonds between the conserved K233 and D234 of AK2A (corresponding to K9 and D10 of MIA40) and AIFM1.

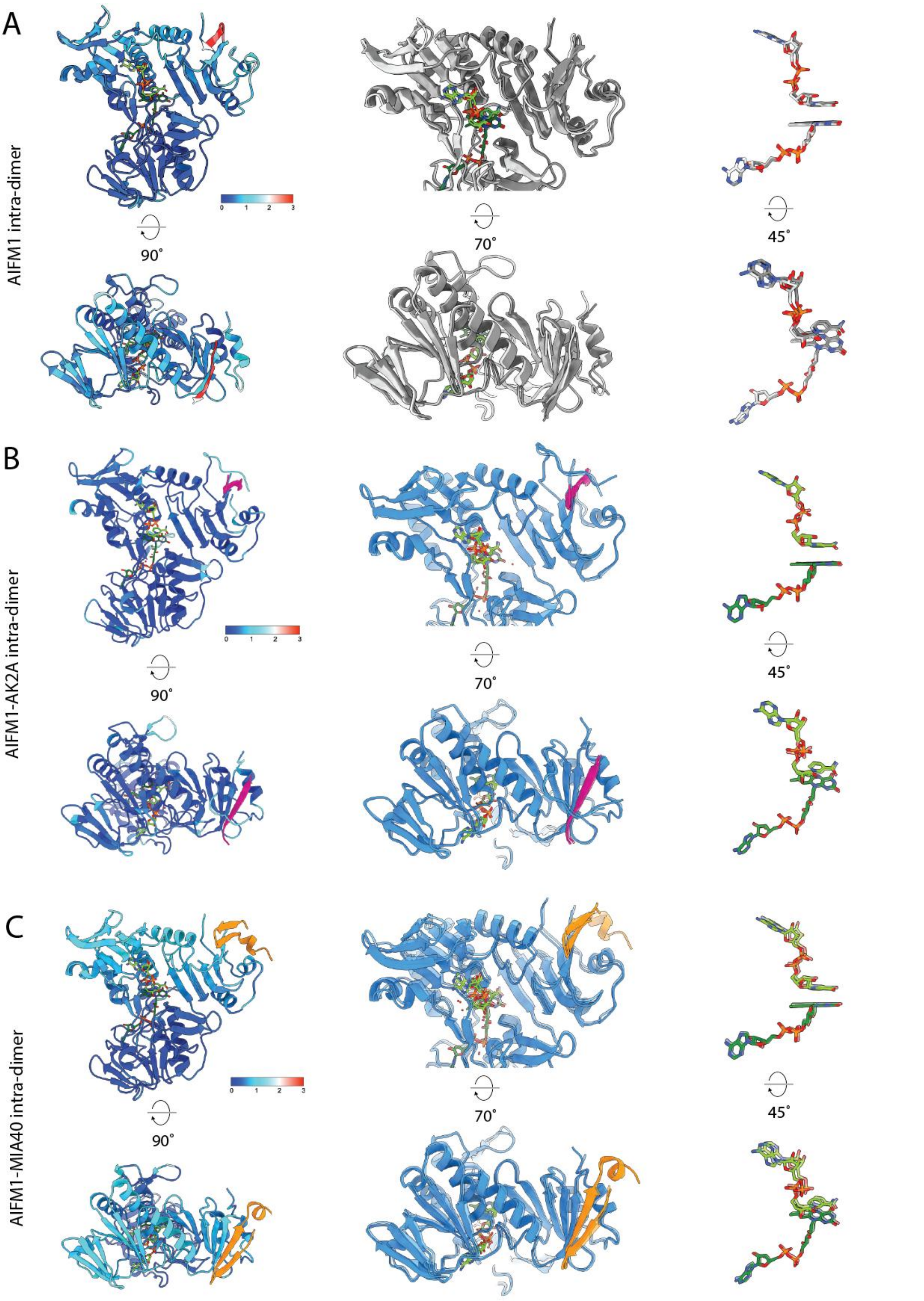

Figure S11: Structural variability between protomers within each AIFM1 dimer of each complex related to Figure 4.

AIFM1 monomers within each complex were superimposed using UCSF ChimeraX at N-terminal residues including the dimer interface (aa 232-257, 404-434 and 440-450). Left panels: models colored

according to the root-mean-square deviation (rmsd) of the Cα atoms. Color code: blue = 0 Å, cyan = 1 Å, white = 2 Å, red = 3 Å. Middle panels: enlarged view of an overlay of both monomer models after alignment, one of the models transparent, to highlight displacement. Right panels: enlarged view of the NAD and FAD cofactors of both monomers after alignment of the models to visualize the direction and extent of variability. **(A)** AIFM1 dimer. **(B)** AIFM1-AK2A complex. **(C)** AIFM1-MIA40 complex.

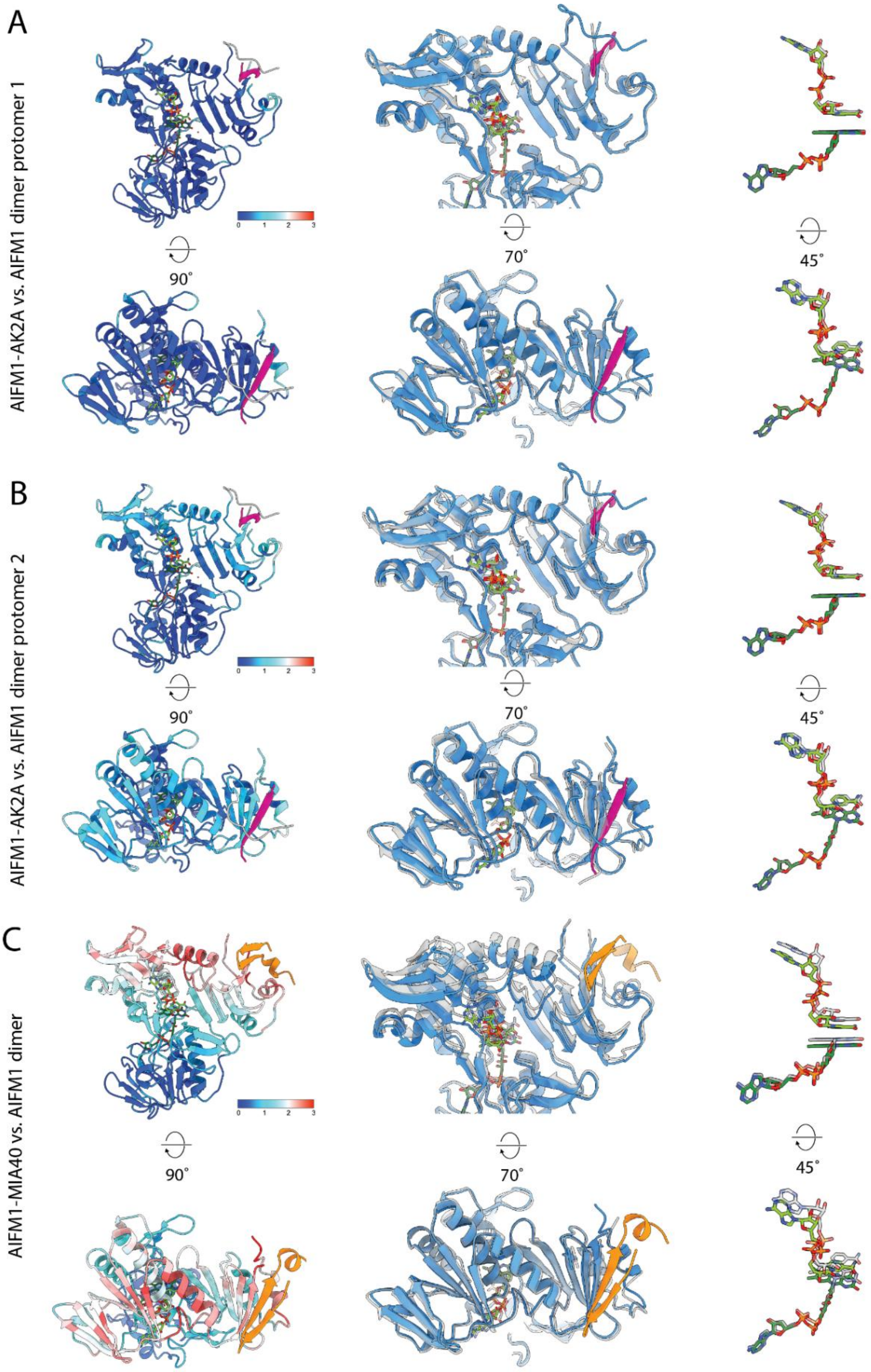

Figure S12: Structural variability between monomers of AIFM1-MIA40 and AIFM1-AK2A complexes as compared to the AIFM dimer related to Figure 4.

Monomers of the AIFM1-AK2A complex (**A, B**) or the AIFM1-MIA40 complex (**C**) were superimposed using UCSF ChimeraX at the N-terminal β-sheets (aa 128-165 and aa 212-261). Left panels: models colored according to the root-mean-square deviation (rmsd) of the Cα atoms. Color code: blue = 0 Å,

cyan = 1 Å, white = 2 Å, red = 3 Å. Middle panels: enlarged view of an overlay of both monomer models after alignment, one of the models transparent, to highlight displacement. Right panels: enlarged view of the NAD and FAD cofactors of both monomers after alignment of the models to visualize the direction and extent of variability. For AIFM1-AK2A, the alignment of one monomer to each of the AIFM1 dimer protomers is shown. For AIFM1-MIA40, only the monomer-to-monomer comparison with the strongest variability is shown.

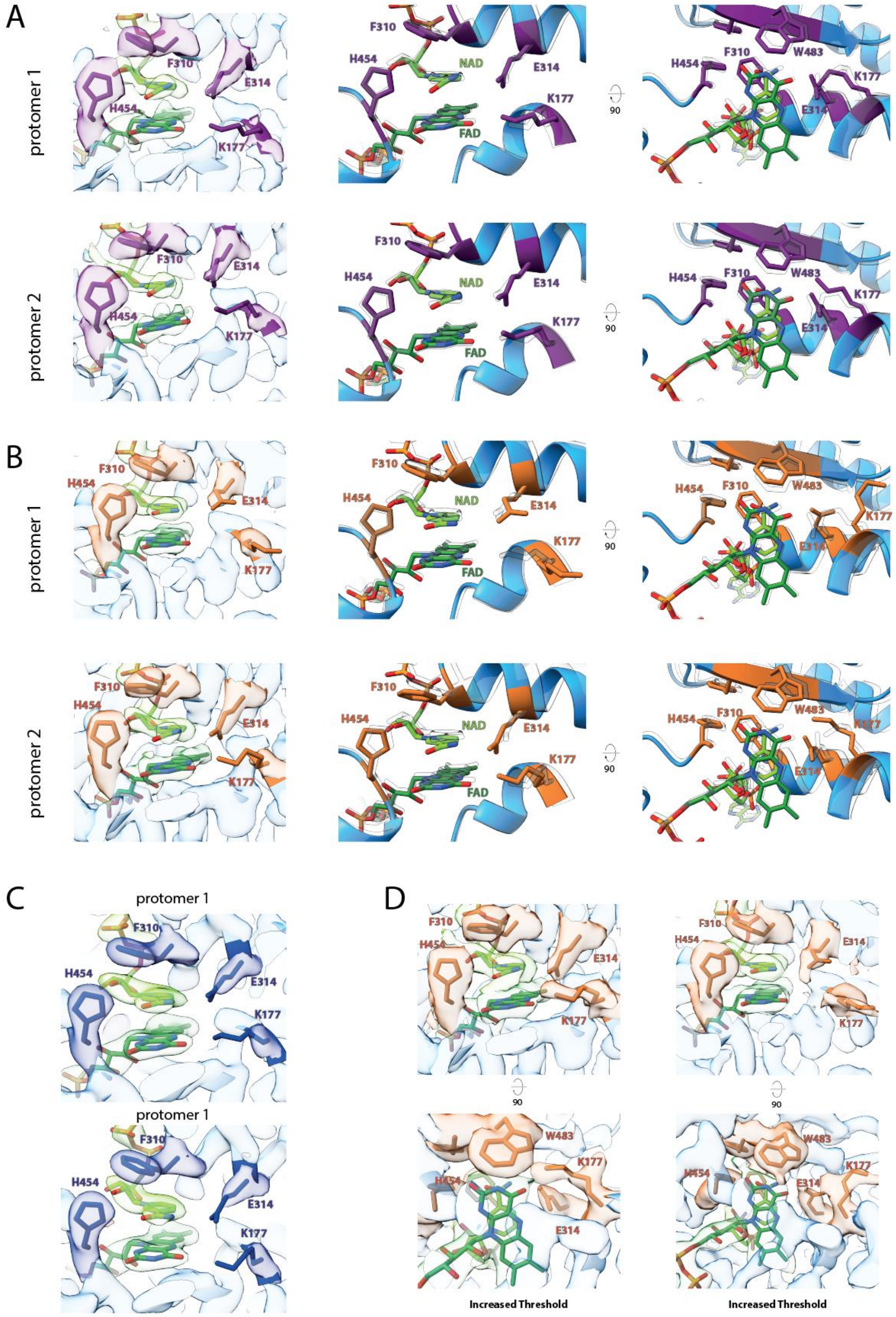

Figure S13: Structural details and conformational changes of the active site of AIFM1 related to Figure 4.

(A) Active site of the two protomers (top and bottom row) of AIFM1-AK2A. Left panel: Local cryo-EM density (semi-transparent) and fitted model, cofactor binding residues shown as stick representation. Middle and right panels: Enlarged views of the AIFM1 active site, residues stabilizing the cofactors shown as stick representation and in purple. The model of the AIFM1 dimer is shown transparent and as an overlay.

(B) Active site of the two protomers (top and bottom row) of MIA40-bound AIFM1. Left panel: Local cryo-EM density (semi-transparent) and fitted model, cofactor binding residues shown as stick representation. Middle and right panels: Enlarged views of the AIFM1 active site, residues stabilizing the cofactors shown as stick representation and in orange. The model of the AIFM1 dimer is shown transparent and as an overlay.

(C) Cryo-EM density of the active site of the AIFM1 dimer of both protomers (top and bottom panels) with the respected atomic model fitted.

(D) Cryo-EM density of the active site of AIFM1-MIA40 of both protomers (left and right panels) shown at increased threshold (bottom panels) to visualize weaker densities indicating flexibility.

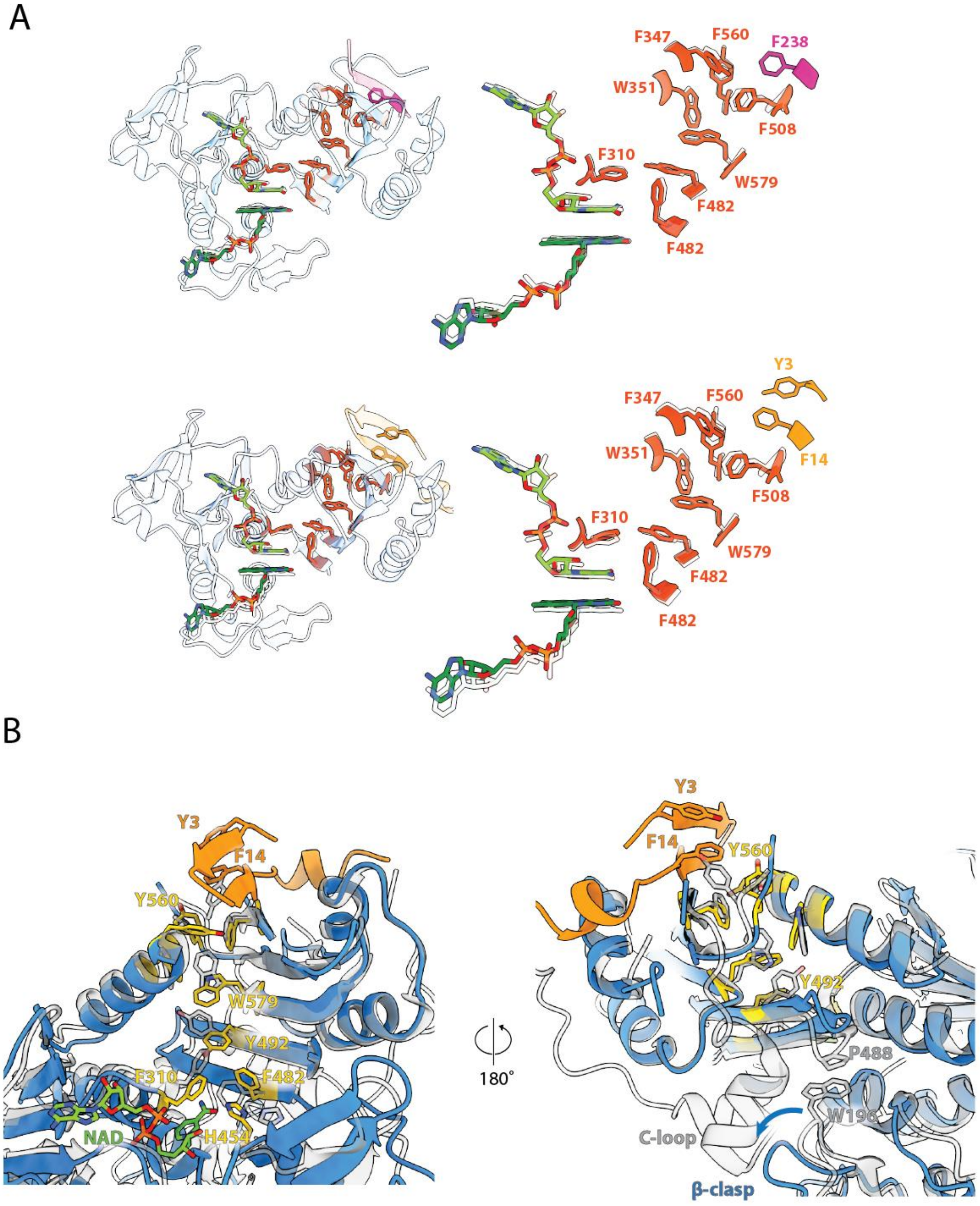

Figure S14: Aromatic tunnel of AIFM1 and conformational stabilization of the aromatic tunnel by AK2A and MIA40 binding related to Figure 4.

(A) Aromatic tunnel of AIFM1. Left panel: aromatic residues of AIFM1 (dark orange), AK2A (top, purple) and MIA40 (bottom, light orange) forming the aromatic tunnel within an AIFM1 monomer, linking the cofactor binding site and protein surface. Right panel: enlarged detail of amino acids and the NAD and FAD cofactors involved. Transparent model: overlay of the AIFM1 dimer lacking AK2A or MIA40 binding.

(B) Structural details of aromatic aa side chains forming the ‘aromatic tunnel’ and the conformational impact of MIA40 binding (orange). Aromatic tunnel residues and the NAD binding H454 are highlighted in yellow. The AIFM1 model in the monomeric, oxidized conformation (PDB 4BV6, [3]) is shown as a grey, transparent overlay.

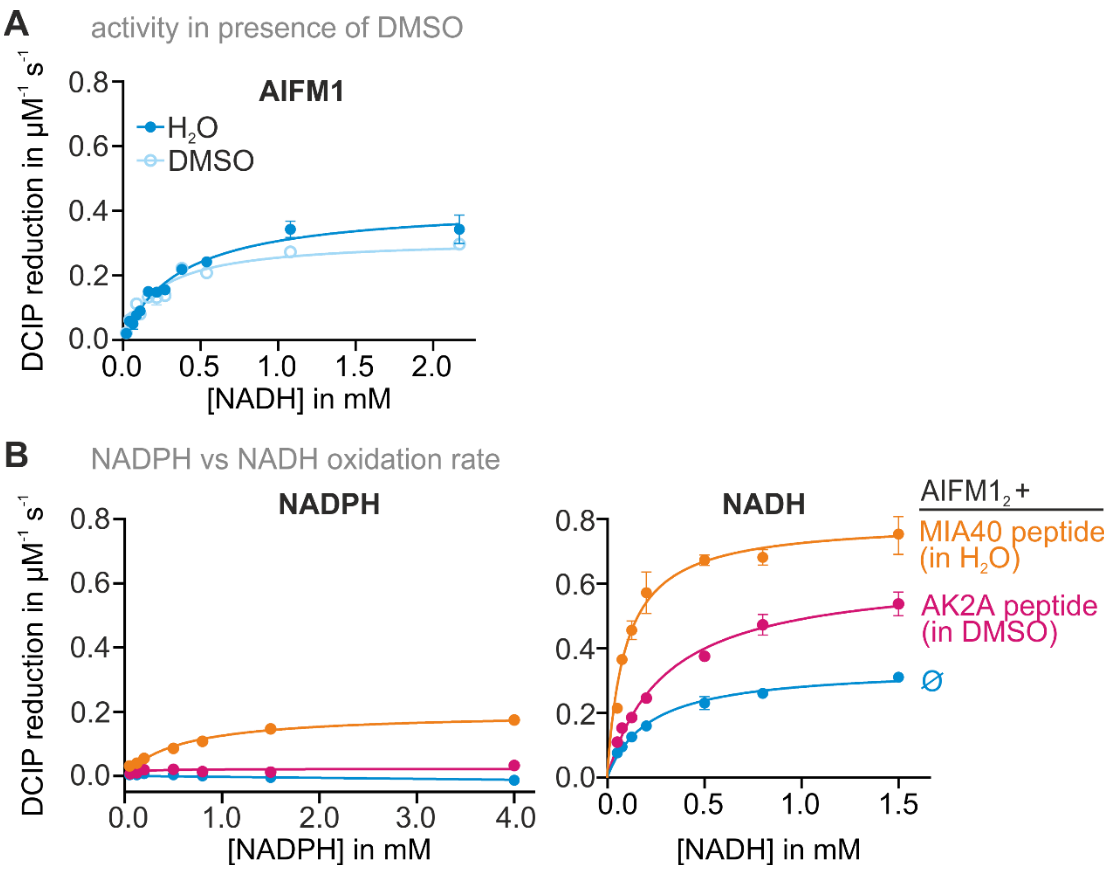

Figure S15: AIFM1 changes its activity upon binding of AK2 or MIA40 related to Figure 4.

(A) Activity of AIFM1 towards NADH and DCIP does not change in the presence of low amounts of DMSO.

(B) Addition of AK2 or MIA40 to AIFM1 increases its activity towards NADH and DCIP. AIFM1 activity towards NADPH is in this assay negligible. Only binding of MIA40 to AIFM1 results in a minor increase of AIFM1 activity. Please observe the differences in the X-axis of the two plots in this panel.

REFERENCES

[S1] Punjani, A., Rubinstein, J.L., Fleet, D.J., and Brubaker, M.A. (2017). cryoSPARC: algorithms for rapid unsupervised cryo-EM structure determination. Nat Methods *14*, 290-296. 10.1038/nmeth.4169.

[S2] Bepler, T., Morin, A., Rapp, M., Brasch, J., Shapiro, L., Noble, A.J., and Berger, B. (2019). Positive-unlabeled convolutional neural networks for particle picking in cryo-electron micrographs. Nat Methods *16*, 1153-1160. 10.1038/s41592-019-0575-8.

[S3] Ferreira, P., Villanueva, R., Martinez-Julvez, M., Herguedas, B., Marcuello, C., Fernandez-Silva, P., Cabon, L., Hermoso, J.A., Lostao, A., Susin, S.A., and Medina, M. (2014). Structural insights into the coenzyme mediated monomer-dimer transition of the pro-apoptotic apoptosis inducing factor. Biochemistry *53*, 4204-4215. 10.1021/bi500343r.
